# Country-Level Relationships of the Human Intake of N and P, Animal and Vegetable Food, and Alcoholic Beverages with Cancer and Life Expectancy

**DOI:** 10.3390/ijerph17197240

**Published:** 2020-10-03

**Authors:** Josep Penuelas, Tamás Krisztin, Michael Obersteiner, Florian Huber, Hannes Winner, Ivan A. Janssens, Philippe Ciais, Jordi Sardans

**Affiliations:** 1 CSIC, Global Ecology Unit CREAF-CSIC-UAB, 08193 Cerdanyola del Vallès, Spain; j.sardans@creaf.uab.cat; 2 CREAF, 08193 Cerdanyola del Vallès, Spain; 3 International Institute for Applied Systems Analysis (IIASA), Ecosystems Services and Management, Schlossplatz 1, A-2361 Laxenburg, Austria; krisztin@iiasa.ac.at (T.K.); michael.obersteiner@gmail.com (M.O.); 4 Paris Lodron University of Salzburg, Mönchsberg 2a, A-5020 Salzburg, Austria; florian.huber@sbg.ac.at (F.H.); hannes.winner@sbg.ac.at (H.W.); 5 Austrian Institute of Economic Research (WIFO), Arsenal Objekt 20, A-1030 Vienna, Austria; 6 Research Group Plants and Ecosystems (PLECO), Department of Biology, University of Antwerp, B-2610 Wilrijk, Belgium; ivan.janssens@uantwerpen.be; 7 Laboratoire des Sciences du Climat et de l’Environnement, IPSL, 91191 Gif-sur-Yvette, France; philippe.ciais@lsce.ipsl.fr

**Keywords:** food security, cancer, stoichiometry, protein intake, nitrogen, phosphorus, kilocalories

## Abstract

Background: The quantity, quality, and type (e.g., animal and vegetable) of human food have been correlated with human health, although with some contradictory or neutral results. We aimed to shed light on this association by using the integrated data at country level. Methods: We correlated elemental (nitrogen (N) and phosphorus (P)) compositions and stoichiometries (N:P ratios), molecular (proteins) and energetic traits (kilocalories) of food of animal (terrestrial or aquatic) and vegetable origin, and alcoholic beverages with cancer prevalence and mortality and life expectancy (LE) at birth at the country level. We used the official databases of United Nations (UN), Food and Agriculture Organization of the United Nations (FAO), Organization for Economic Co-operation and Development (OECD), World Bank, World Health Organization (WHO), U.S. Department of Agriculture, U.S. Department of Health, and Eurobarometer, while also considering other possibly involved variables such as income, mean age, or human development index of each country. Results: The per capita intakes of N, P, protein, and total intake from terrestrial animals, and especially alcohol were significantly and positively associated with prevalence and mortality from total, colon, lung, breast, and prostate cancers. In contrast, high per capita intakes of vegetable N, P, N:P, protein, and total plant intake exhibited negative relationships with cancer prevalence and mortality. However, a high LE at birth, especially in underdeveloped countries was more strongly correlated with a higher intake of food, independent of its animal or vegetable origin, than with other variables, such as higher income or the human development index. Conclusions: Our analyses, thus, yielded four generally consistent conclusions. First, the excessive intake of terrestrial animal food, especially the levels of protein, N, and P, is associated with higher prevalence of cancer, whereas equivalent intake from vegetables is associated with lower prevalence. Second, no consistent relationship was found for food N:P ratio and cancer prevalence. Third, the consumption of alcoholic beverages correlates with prevalence and mortality by malignant neoplasms. Fourth, in underdeveloped countries, reducing famine has a greater positive impact on health and LE than a healthier diet.

## 1. Introduction

The human diet and the intake and proportions of food from different sources have always been associated with human health [1,2]. Several epidemiologic studies in the last 20–30 years that followed cohorts and analyzed hospital databases have investigated the association of the food source (animal or vegetable, distinct types of meat, or cooking methods) and molecular constituents (e.g., proteins and fats) with the risks of various types of cancers (e.g., [3,4,5]). The results of these studies are not always consistent, preventing definitive conclusions and demonstrating the need of deeper analyses of data from epidemiological studies where the relationships between different diet sources and cancer risks can be weak and/or confounded by other accompanying variables [6,7,8]. However, there is a consensus on the fact that several types of food are associated with several types of cancer. Among food types, the most frequently cited by international bodies on cancer risk assessment such as the International Agency for Research on Cancer [9,10] include alcoholic beverages, coffee, tea, meat, and red meat.

Most studies have detected significant links between different diets and some types of cancers ([3,4,5,11,12], among several others). For example, most studies have found a positive link between a high intake of animal food and colon and colorectal [3,4,13], prostate [14], bladder [11], breast [15,16], and head/neck [12] cancers. The associated risk for meat is even higher when meat has been cooked at high temperature, since cooking meat at high temperatures can generate carcinogens such as heterocyclic amines [14,17]. These links of cancer with animal foods are especially strong for processed meat [3]. This strong association with processed meat has been attributed to substances such as nitrites added during industrial processing [17]. Some epidemiologic studies have been supported by laboratory studies linking the effect of carcinogens to their concentrations in red meat, such as the free and glycoside-bound forms of non-human sialic acid and N-glycolylneuraminic acid [18]. Some studies, however, have not found relationships between animal foods and some other types of cancers such as ovarian cancer (e.g., Schulz et al., 2007 [19]). Other reports have found a positive association between egg intake and breast cancer in women older than 55 years [20] and between the total intake of proteins (regardless of source) and the risk of prostate cancer [21]. The relationship between the intake of aquatic animals and cancer risk is less clear. Some studies have observed a reduced risk of the recurrence of prostate cancer if the intake of red meat was at least partially substituted by fish or poultry [4]. Some studies, though, have not found relationships between the level of intake of marine animals and some types of cancer, such as head/neck cancers (e.g., Perloy et al., 2017 [12]).

On the contrary, a higher intake of fresh, non-processed vegetables has been frequently associated with lower risks of some cancers such as breast [15,22] and colorectal [23,24] cancer and generally of cancers of the digestive tract [25]. Some in vitro studies have found that some compounds in some vegetables such as isothiocyanates (sulforaphane, glucoraphanin) and flavonoids (anthocyanidins, flavones, flavones) protect against the risk of carcinogenesis [23,26,27], consistent with these studies.

A positive association between the consumption of alcoholic beverages and the risk of certain cancers has also been widely established [28,29]. A meta-analysis review of epidemiological published reports in scientific literature showed that moderate consumption (up to two standard drinks per day) of alcoholic beverages increases the risk of oral cavity, esophagus, stomach, colon, liver, rectum, larynx, pancreas, breast, and ovary malignant neoplasms [30], while moderate-to-high alcoholic beverage consumption (more than four standard drinks per day) was associated with the risk of enhancement of prostate and pancreas cancers [30]. Several posterior studies have consistently provided similar results associated with oral cavity [31], lung [24], liver [32,33], esophagus [14,32], colorectal [14], breast [14,34,35], or larynx [31,36] cancers. However, a few studies did not find relationships between certain types of cancer and alcohol intake, such as Webb et al., (2004) [37] regarding ovarian cancer and alcohol intake relationships. Analyzing the relationships of the elemental composition of food, e.g., its nitrogen (N) and phosphorus (P) contents and their ratios (N:P), with indicators of human health may also identify associations between diet composition and human health, including cancer risk and longevity. Recent studies that have related ecological stoichiometry (mostly the N:P ratio) to human health have been promising. Rapidly growing tumors tend to have high ribosome contents and key oncogenes closely affiliated with the regulation of ribosome biogenesis and tumor development have physiological impacts on patient phosphate metabolism, consistent with the growth rate hypothesis (GRH) [38]. The GRH has been central in studies of ecological stoichiometry [39] and states that elevated rates of growth are linked to elevated demands for P for the synthesis of P-rich ribosomal RNA (rRNA) [39,40,41,42]. The principle is that organisms must increase their allocation of P to P-rich rRNA to meet the elevated demand for protein synthesis required for rapid growth. The N:P ratio and rate of growth are thus linked by the intimate connections between the allocation of P to ribosomes and the allocation of N to protein synthesis [43]; so, high growth rates of cells, tissues, organs, and organisms are thus related to low N:P ratios, especially when N and P are present in high cell concentrations. Elser et al., (2007) [44] observed that lung and colon tumors had significantly higher (approximately twofold) P and RNA contents and lower N:P ratios than paired normal tissue and that P in RNA contributed a significantly larger fraction of total biomass P in malignant relative to that in normal tissues, consistent with the GRH. Data for renal and hepatic tumors, however, did not support the GRH. Human health may thus depend on optimum stoichiometry by adequate life function, including the mechanism for N:P homeostasis maintenance and the intake of N and P.

Beyond the simple link between food composition and cancer risk, food composition can also affect other variables associated with human health and, thus, with life expectancy (LE) at birth in human populations. For example, some fruits and vegetables can help to prevent or be used to treat chronic human diseases [45]. Epidemiological and statistical studies have found that some diets, such as Mediterranean, Japanese, and vegetarian diets, are correlated with lower risks of several mortal illnesses and, thus, with average lifespan [12]. Both food quality and quantity (calorie intake] have also been correlated with human health and longevity [46,47], although some studies failed to find clear relationships (e.g., Shanley & Kirkwood, 2006 [48]). The intake of some nitrogenous molecules has been correlated with human health and the risk of important diseases [32], and diets rich in proteins have been associated with the risk of several digestive, renal, and vascular diseases [2]. Vegetables excessively fertilized with nitrates can accumulate high levels of these toxic nitrogenous chemicals, with several health risks to components of food webs, including humans [49]. We should thus expect a potential global impact on human health from the increasing intensification of N fertilization in recent decades [18,19,45]. For example, N fertilization in wheat crops increased at the global scale from approximately 10 kg N ha−1 y−1 in 1961 to 100 kg N ha−1 y−1 in 2015 [50]. High rates of N fertilization in wheat crops have been associated with high protein contents in wheat grains and flour [50], so we should expect higher N intakes during this period, which could thus have an impact on human health and, therefore, LE at the global scale. The amount of P in diets may also affect human health and LE, both for deficits and excesses [34,51,52]. Apart from cancer risk rise, alcohol consumption has been associated with higher incidences of other main causes of human death. Even though moderate alcohol has been associated with reduced heart failures in early adult-age in some studies [53], its continuous consumption is related with increased heart failures [39,48,54], and in general with a reduction of human life length [17,55].

The quantity, quality, and type (e.g., animal and vegetable) of human food and beverages have, thus, been correlated with cancer and LE, although mostly at the population level and with many uncertainties. We aimed to shed light on this association in two ways. First, by using the integrated data at the country level in the official databases of United Nations (UN), Food and Agriculture Organization of the United Nations (FAO), Organization for Economic Co-operation and Development (OECD), World Bank, World Health Organization (WHO), U.S. Department of Agriculture, U.S. Department of Health, and Eurobarometer. Second, by focusing the study not only on biochemical compounds (e.g., proteins, alcohol) and food origin (terrestrial animals, marine animals, or plants) but also on the relationship of elemental (N and P) compositions and stoichiometries (N:P ratios) with cancer prevalence and mortality and with LE. We hypothesized that higher N intake, lower N:P intake ratios, terrestrial animal food, and alcoholic beverages would be associated with cancer and shorter LE, whereas on the contrary, aquatic animals and vegetables would be associated with less cancer and longer LE.

## 2. Materials and Methods

### 2.1. Data Collection and Preparation

We gathered the data from the most relevant and important world databases with available information about food intake, food composition, and human health indexes at country level and global scale. Our dataset on cancer mortality stems from the WHO (World Health Organization) Mortality Database, which comprises national mortality and incidence statistics as reported by countries’ health organizations, classified according to International Classification of Diseases (ICD) guidelines. The database itself contains raw mortality numbers, with observations ranging from 1960 to 2017. The data are classified using ICD version 7 to 10, depending on the reporting country. The ICD classifications were harmonized using the cancer dictionary from the WHO’s International Agency for Research on Cancer. To obtain death rates per country, the WHO’s corresponding figures on population were used. In order to correct for age structure-specific differences amongst the countries, we utilized the world standard population with the corresponding weighing scheme of age groups [50].

The data on cancer prevalence stem from the OECD (Organization for Economic Co-operation and Development) and from the WHO’s CI5plus databases. Observations from the CI5plus database were selected, which contain a representative sample of the whole population. Standardized rates for the prevalence data were calculated using the same method as in the case of cancer mortality.

We calculated the annual per capita intake of proteins, Kcal, N, P, and N:P (mass basis) for all OECD countries as follows: ∑ annual intake of each food group (1) × mean N or P concentration for each food group (2). (1) Data from FAO (Food and Agriculture Organization of the United Nations), and (2) Data from INFOODS Food Composition Database for Biodiversity, USDA, and Danmarks Tekniske Universitet (DTU) Fodevareinstituttet. We estimated the N and P concentrations for each food group in the FAO databases using the databases in (2). These databases contained proteins, Kcal, N, and P concentrations for various food items. We grouped these food items into sets corresponding to the FAO food groups and calculated the corresponding average intake for each group. We used the average as the final value when data for N and/or P concentrations were provided by more than one database for the same food group. The per capita intakes of kilocalories and proteins for each country, year, and type of food were obtained directly from the FAO database.

The increases in annual per capita intakes of P, N, proteins, and kilocalories with regard to cancer mortality and LE for each country in the 2000s relative to the values in the 1960s were estimated for all countries for which information was available. We obtained data for LE, GDP (gross domestic product) per capita, and percentage of urban population for each country and year from the World Bank. We obtained HDI (human development index) for each country and year from the United Nations Development Program. We obtained the country’s population mean age (MA) for each country and year from WHO.

### 2.2. Statistical Analyses

#### Bayesian Models

We analyzed three different datasets, one for each response variable studied:

1. In the first analysis, we attempted to explain the average prevalence of total malignant neoplasms, as well as breast, cervix, colon, lung, and prostate cancers using our set of averaged indicators in the period 1998–2010 (N= 52).
2. In the second analysis, we attempted to explain the average deaths (per 100,000 population) during the period 1960–2010 due to malignant neoplasms, as well as breast, cervix, colon, lung, and prostate cancers using our set of averaged covariates in the period 1960–2010 (N= 85).
3. Finally, we regressed the average increase in life expectancy from 1960 to 2010 on our set of covariates measured in their averages over the period 1960–2010 (100). We also regressed the increase in life expectancy on the change of our set of covariates during this period.

Within each analysis, we used as explanatory variables the total set of available nutritional covariates, containing observations on N, P, N/P, kcal, proteins, and total kg consumption from terrestrial-animal, vegetables, terrestrial-animal/vegetables, aquatic-animal, and alcoholic sources. Additionally, we used GDP per capita, median age of population, as well as the human development index as control variables.

In order to draw inference from the impact of nutritional determinants on average cancer prevalence, mortality, and life expectancy, we employed a flexible Bayesian framework. The advantage of this framework was that it allowed us to (i) flexibly deal with the problems of severe collinearity between our explanatory variables, (ii) and to alleviate the problem of over-fitting. The latter was especially a concern, given the number of limited observations (ranging from 52 to 100), and the relatively high number of variables of interest (K=33 covariates).

To alleviate the collinearity within the explanatory variables, we clustered them based on their covariance matrix and using the hclust R package, together with the semi-automated algorithm from Kelley et al. (1996) [35], to arrive at five distinct clusters (Supplementary Table S1). From each cluster, we used principal components analysis to obtain the three eigenvectors associated with the highest eigenvalues. Within each cluster, these covered over 90% of variation within the covariates. The eigenvectors obtained this way were used as explanatory variables in our regression analysis. This dimension reduction of the explanatory variables greatly reduces collinearity, while still capturing the key variation within the observed variables. Additionally, the estimated impacts of the factors can be mapped back to the explanatory variables using the precalculated factor loadings.

This model can be easily estimated using maximum likelihood estimation. However, since one of the goals of this study was to analyze the driving forces that correlate with cancer prevalence rates across countries, we needed a more flexible approach that allows to assess uncertainty with respect to the underlying structural model. For this purpose, we used a form of Bayesian variable selection, labeled as the stochastic search variable selection (SSVS) prior (see [48,52]). The advantage of this approach is that the impact of covariates that are estimated a posteriori to have relatively low importance are shrunk toward zero, thus increasing the effective degrees of freedom, and allowing us to draw inference on the relative importance of covariates. We carried out model estimation using a Markov chain Monte Carlo (MCMC) algorithm, details of which, along with the prior specification, are provided in the Supplementary Materials.

### 2.3. Reduced Major-Axis Analyses, Generalized Linear Models, and Principal Component Analyses

We also used direct reduced major-axis analysis to simply visually assess the bivariate relationships among cancer mortality (for 1960–2010), cancer prevalence (1998–2010), and the change in LE between the 1960s and the 2000s in 100 countries with the corresponding values of each explanatory variable (per capita total intakes and intakes from terrestrial animal foods, aquatic animal foods and plant/vegetable foods, proteins, kcal, alcohol, N, P, and N:P ratio). To analyze the effect of other potentially influencing variables on these commented bivariate relationships, we also conducted generalized linear models to analyze these relationships and included in the models the corresponding per capita LE, GDP, HDI, and national MA as independent variables together with the mentioned variables of food intake. We used the gls function to fit a linear model using the generalized least squares with the R-package nlme [56]. We coupled these analyses with the function stepAIC to select in each case the best model (lower AIC) with the MASS package [55] from the saturated models with LE, GDP, HDI, national MA, and one-different food variable in each saturated model as independent variables. Finally, we also conducted PCAs with the data set of explanatory variables (Supplementary Table S1) and per capita country neoplasm prevalence and mortality and LE and increase of LE in the last decades.

## 3. Results

**Figure 1:**
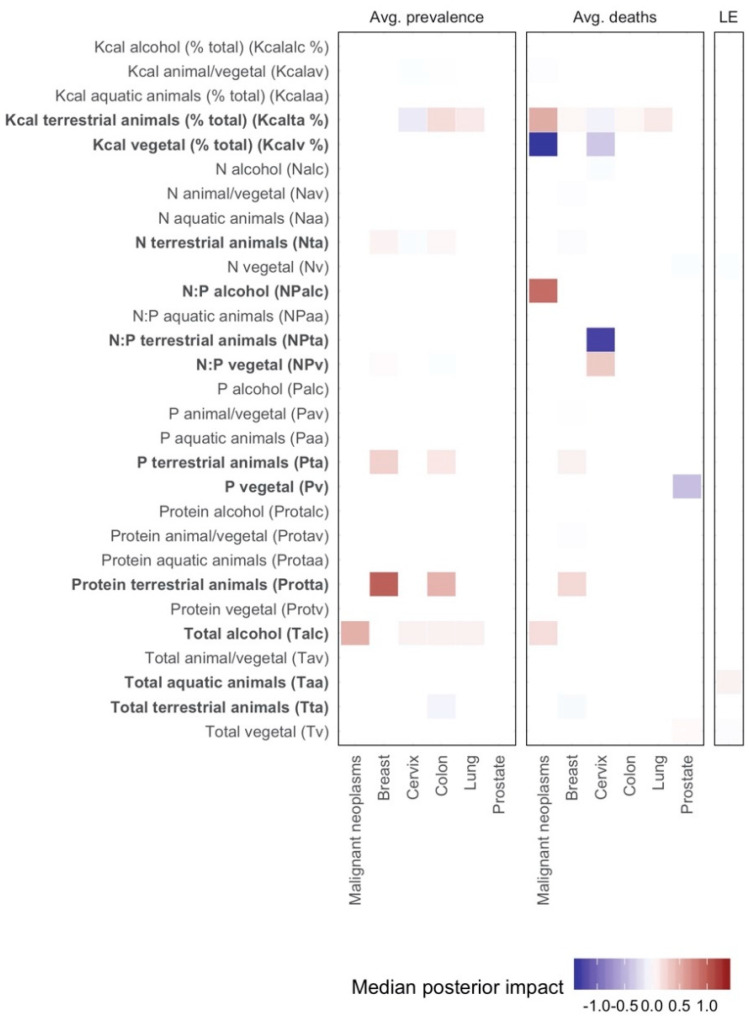
Median posterior coefficient estimates from the Bayesian econometric models. Each panel corresponds to datasets (for prevalence, deaths and LE—life expectancy; the shading indicates the magnitude of the posterior impact. Not significant estimates under 95% confidence intervals are shaded in white. Results are based on three eigenvectors associated with the highest eigenvalues of each cluster (Supplementary Table S1). Predictors discussed in the text are denoted in bold.

In order to analyze the impacts of nutrient consumption on cancer prevalence, mortality, and life expectancy, we used the flexible Bayesian regression framework for inference. Figure 1 contains the corresponding coefficient estimates for the main variables of interest (control variables were omitted for the sake of readability; detailed results are available in the Supplementary Materials) as explanatory variables of cancer prevalence, cancer mortality, and life expectancy.

**Figure 2:**
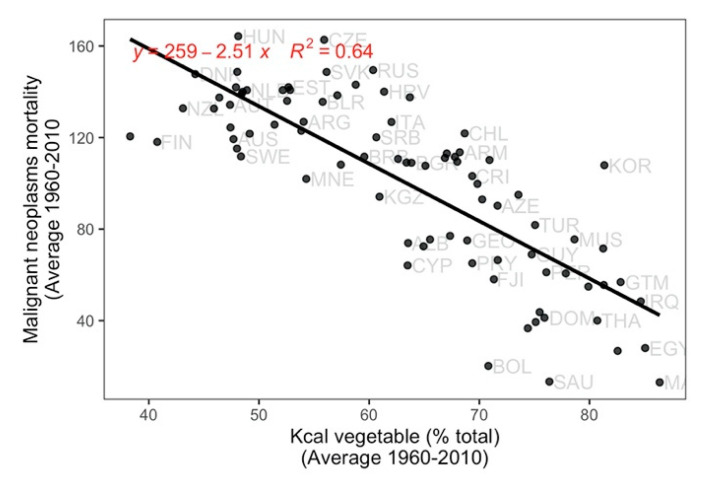
Relationship of the mortality (per 100,000 population) of malignant neoplasms (total number of cancers) with country annual mean percentage of total Kcal from vegetable sources. *p* < 0.001.

**Figure 3:**
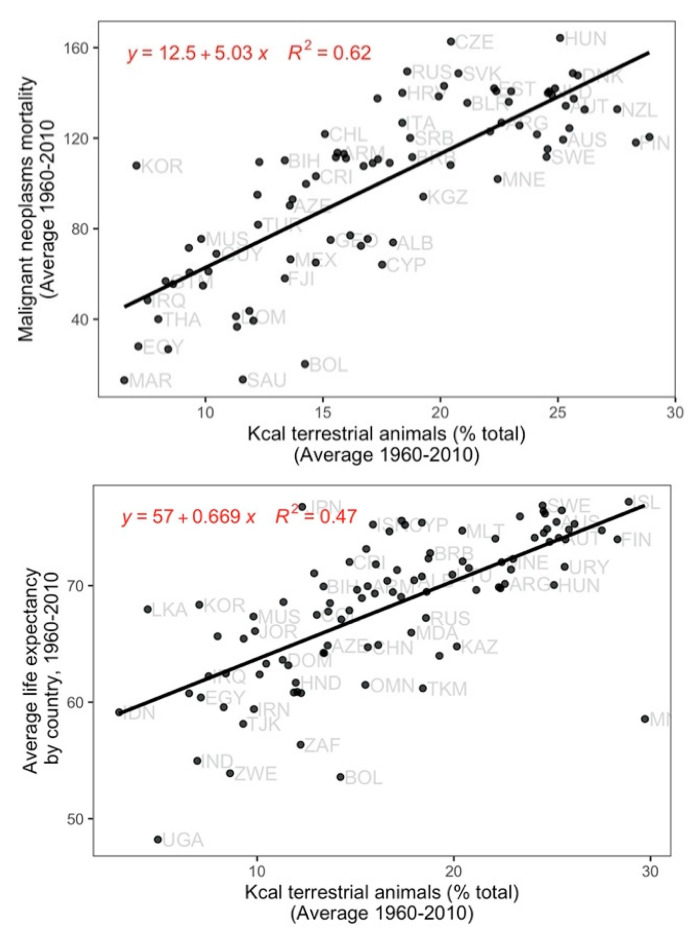
Relationship of the mortality (per 100,000 population) of malignant neoplasms (total number of cancers) and the average life expectancy per country from 1960 to 2010 with country annual mean percentage of total Kcal from terrestrial animal sources. *p* < 0.001.

In kilocaloric terms, a higher consumption from vegetable sources was associated with a significantly lower mortality from total malignant neoplasms, as well as from neoplasms of the cervix (Figure 1 and Figure 2). A higher percent of kilocaloric consumption from terrestrial animal sources was conversely associated with a higher prevalence of lung and prostate neoplasms, and deaths from total malignant neoplasms, as well as malignant neoplasms of the breast, colon, and lung (Figure 1 and Figure 3).

A higher N consumption from terrestrial animal sources was associated with a higher prevalence of malignant neoplasms of the breast, cervix, and colon (Supplementary Table S2). Similarly, higher P consumption from terrestrial animal source was associated with a higher breast cancer prevalence and mortality, as well as the prevalence of malignant neoplasms of the colon (Supplementary Table S2). This association and the others described here were separable from the associations with other colinear variables, for example, from higher animal % in intake, since we used a form of principal component regression analysis (through the clusters), which should be able to trace this out (tests such as the VIF (variation inflation factor) also indicated low residual multicollinearity among the variables described here). A higher P consumption from vegetable sources was associated with less deaths from prostate cancer. Regarding the ratio of N to P consumption, no significant relationship was found between N:P ratio and malignant neoplasm prevalence (Figure 1).

**Figure 4:**
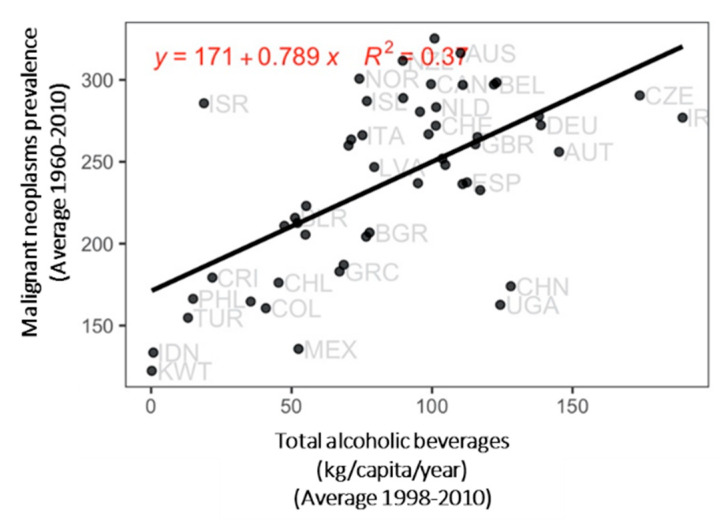
Relationship of the prevalence (per 100,000 population) of malignant neoplasms per country with the annual mean intake of alcoholic beverages. *p* < 0.001.

The protein intake from terrestrial animal sources was linked with higher prevalence of breast and colon cancers, as well as increased deaths from breast cancer (Supplementary Table S3). The total per capita alcohol consumption was linked to high incidence and mortality from malignant neoplasms, as well as high incidence of cervical, colon, and lung cancers but with a weaker relation (Figure 1 and Figure 4). A higher total consumption of aquatic animals was linked with a small, but significant, increase in life expectancy.

The generalized linear models confirmed the association between high prevalence of malignant neoplasms for 1998–2010 and the intakes of N, P, protein, and kilocalories from terrestrial animal sources, particularly for colon, lung, breast, and prostate neoplasms. The association was even stronger when considering the ratio between terrestrial animal/vegetable for total food, N, P, proteins, and kilocalories intakes (Supplementary Table S4). When potentially confounding variables (i.e., those that can also potentially influence population health, such as the national mean age of the population (MA), HDI, LE, and GDP) were also taken into account in the analyses, the best models continued to find positive associations with nutritional sources from intakes of terrestrial animal foods (Supplementary Table S5). The best models also found negative associations between vegetable foods and some malignant neoplasms (Supplementary Table S5).

High mortality from malignant neoplasms (the sum of all types) for 1960–2010 was associated with high national per capita total, N, P, protein, and kilocalories intake from food from terrestrial (not aquatic) animals (Supplementary Table S6). Nevertheless, this effect was not observed in relation to per capita intakes of total, N, P, protein, and kilocalories from aquatic animals for the same period (Supplementary Table S6). When the other variables that can also potentially influence population health, such as MA, HDI, LE, and GDP were also taken into account in the analyses, the best models continued to find positive associations of total, N, protein, and kilocalories intake from terrestrial animal foods with mortality from total, prostate, colon, breast, and lung malignant neoplasms (Supplementary Table S7). The N and P concentrations of terrestrial animal foods were positively related and the N and P concentrations of vegetable foods were negatively related with total neoplasm mortality in the period 1960–2010.

The national LE during the period 1960–2010 was positively associated with the national per capita total food intake and intakes of N, P, protein, and kilocalories mainly from terrestrial animals (Supplementary Table S8). The corresponding linear analyses including GDP, HDI, and MA as independent factors also influencing population health, maintained the positive relationships of terrestrial animal and vegetable food with LE (Figure 3, Supplementary Table S9).

**Figure 5:**
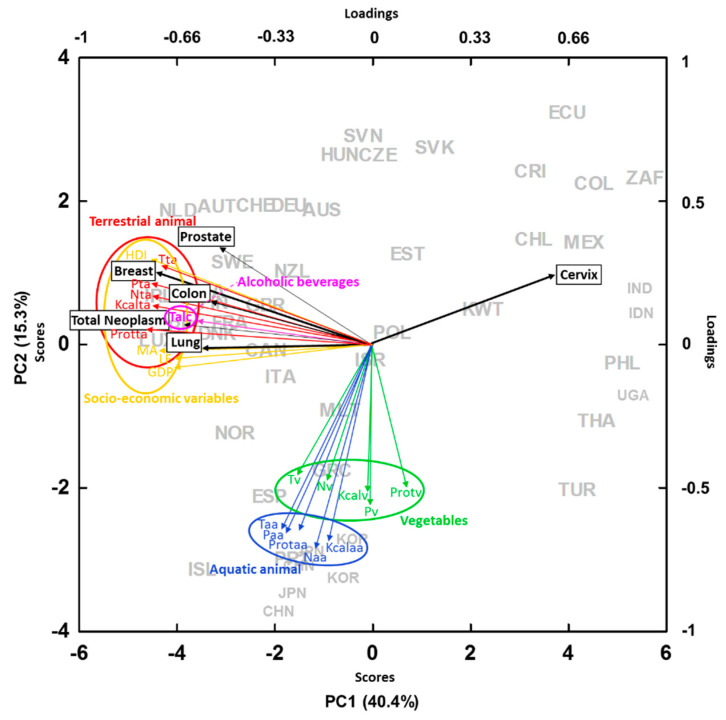
PCA (principal component analysis) with per capita country neoplasm prevalence (L = lung, C = colon, Ce = cervix, P = prostate, TN = total neoplasm, B = breast), different socioeconomic variables (LE = life expectance at birth, AM = age mean, HDI = human development index, GDP = gross domestic product per capita) and food sources (t = total, N = nitrogen intake, P = phosphorus intake, prot = protein and Kcal = kilocalories per capita intake) from terrestrial animal sources (ta) (red), vegetables (v) (green), and aquatic animal (aa) (blue) in the period 1990–2010. AUS = Australia, AUT = Austria, BEL = Belgic, CAN = Canada, CHE = Switzerland, CHL = Chile, CHN = China, COL = Colombia, CRI = Costa Rica, CZE = Czech Republic, DEU = Germany, DNK = Denmark, ECU = Ecuador, ESP = Spain, EST = Estonia, FIN = Finland, FRA = France, GBR = United Kingdom, GRC = Greece, HUN = Hungary, IDN = India, IND = Indonesia, IRL = Ireland, ISL = Iceland, ISR = Israel, ITA = Italy, JPN = Japan, KOR = Korea, KWT = Kuwait, LUX = Luxembourg, MEX = Mexico, MLT = Malta, NLD = Netherlands, NOR = Norway, NZL = New Zealand, PHL = Philippines, POL = Poland, PRT = Portugal, SVK = Slovakia, SVN = Slovenia, SWE = Sweden, THA = Thailand, TUR = Turkey, UGA = Uganda, USA = United States of America, ZAF = South Africa.

**Figure 6:**
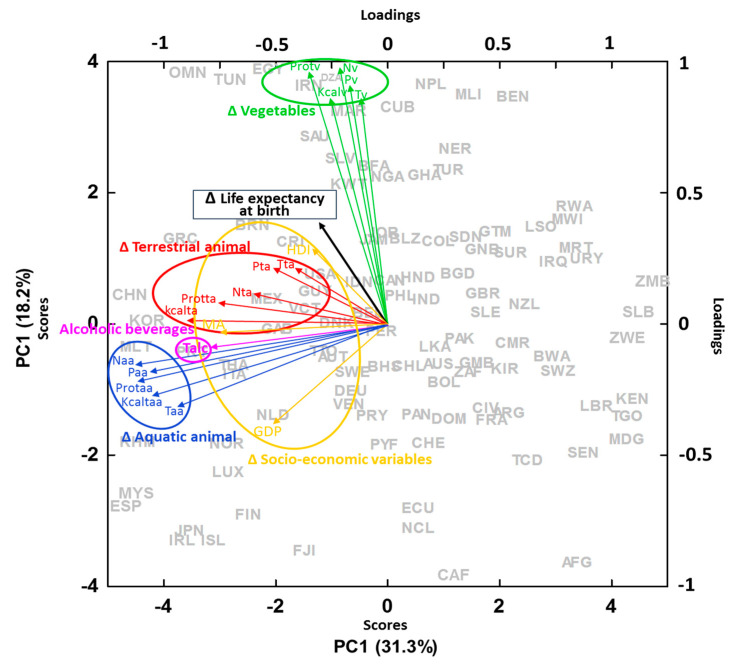
PCA with the increases of mean averages during the period 1960–2010 of country life expectance at birth (LE), different socioeconomic variables (AM = age mean, HDI = human development index, GDP = gross domestic product per capita) and food sources (t = total, N = nitrogen intake, P = phosphorus intake, prot = protein and Kcal = kilocalories per capita intake) from terrestrial animal sources (ta) (red), vegetables (v) (green), and aquatic animal (aa) (blue). AFG = Afghanistan, DZA = Algeria, ARG = Argentina, AUS = Australia, AUT = Austria, BHS = Bahamas, BGD = Bangladesh, BEL = Belgium, BLZ = Belize, BEN = Benin, BOL = Bolivia, BWA = Botswana, BRN = Brunei Darussalam, BFA = Burkina Faso, KHM = Cambodia, CMR = Cameroon, CAN = Canada, CAF = Central African Republic, TCD = Chad, CHL = Chile, CHN = China, COL = Colombia, CRI = Costa Rica, CIV = Cote Ivoire, CUB = Cuba, DNK = Denmark, DOM = Dominican Republic, ECU = Ecuador, EGY = Egypt, SLV = El Salvador, FJI = Fiji, FIN = Finland, FRA = France, PYF = French Polynesia, GAB = Gabon, GMB = Gambia, DEU = Germany, GHA = Ghana, GRC = Greece, GTM = Guatemala, GNB = Guinea-Bissau, GUY = Guyana, HND = Honduras, ISL = Iceland, IND = India, IDN = Indonesia, IRN = Iran, IRQ = Iraq, IRL = Ireland, ITA = Italy, JAM = Jamaica, JPN = Japan, JOR = Jordan, KEN = Kenya, KIR = Kiribati, KOR = Korea, KWT = Kuwait, LSO = Lesotho, LBR = Liberia, LUX = Luxembourg, MDG = Madagascar, MWI = Malawi, MYS = Malaysia, MLI = Mali, MLT = Malta, MRT = Mauritania, MEX = Mexico, MAR = Morocco, NPL = Nepal, NLD = Netherlands, NCL = New Caledonia, NZL = New Zealand, NER = Niger, NGA = Nigeria, NOR = Norway, OMN = Oman, PAK = Pakistan, PAN = Panama, PRY = Paraguay, PER = Peru, PHL = Philippines, PRT = Portugal, RWA = Rwanda, VCT = Saint Vincent and the Grenadines, SAU = Saudi Arabia, SEN = Senegal, SLE = Sierra Leone, SLB = Solomon Islands, ZAF = South Africa, ESP = Spain, LKA = Sri Lanka, SDN = Sudan, SUR = Suriname, SWZ = Swaziland, SWE = Sweden, CHE = Switzerland, THA = Thailand, TGO = Togo, TTO = Trinidad and Tobago, TUN = Tunisia, TUR = Turkey, UGA = Uganda, GBR = United Kingdom, USA = United States of America, URY = Uruguay, VEN = Venezuela (Bolivarian Republic of), ZMB = Zambia, ZWE = Zimbabwe.

The PCA analyses with the data bases of neoplasm prevalence, mortality, and life expectancy showed patterns similar to those of the Bayesian and generalized linear models. The higher the country socioeconomical development, the longer the LE (Supplementary Figure S1) but the higher the prevalence and mortality of malignant neoplasms (except cervix) (Figure 5). Food sources coming from terrestrial animals were associated with higher prevalence of malignant neoplasms, whereas those from aquatic animals or vegetable foods were not (Figure 5). When focusing on the change during the last decades, the increase in LE was strongly associated with an increase in vegetable and animal food intake together with the other socioeconomic indicators of development (Figure 6).

## 4. Discussion

Our Bayesian analysis at the country scale thus confirmed our hypotheses except the one on N:P ratio. When also accounting for the variance explained by the socioeconomic variables of development and richness, positive relationships were found between prevalence and mortality from total, colon, prostate, and respiratory cancers and terrestrial animal intake and alcoholic beverages. These relationships were especially strong for the intake of N and proteins from terrestrial animals. The best (and simpler) linear models also identified these same links, in addition to the links to national GDP, HDI, and MA. High per capita intakes of N, P, protein, and total intake from aquatic animals and especially from vegetables had instead negative relationships with cancer prevalence. No negative association was found between N:P ratio and neoplasm malignant prevalence. The different results for cervix cancer suggest that the environmental causes of this type of cancer can be very distinct from those underlying colon, respiratory, and prostate cancers. Cervix cancer may be associated with other factors affecting the quality of life not directly associated with the food traits we studied.

We expected a positive relationship between national LE at birth and total food intakes. The results also confirmed these expectations; food intakes notably accounted for LE in most models, even when including the shifts in GDP, HDI, and initial MA for each country. The data thus clearly indicated that the key variables at large scales accounting for cancer mortality are mostly related with the high intake of animal products mostly characteristic of the rich countries (Figure 5). These rich countries, on the other hand, have better life conditions that allow longer LE (Figure 6) and, thus, are also more susceptible to cancer. On the other hand, the general and substantial lengthening of LE at the global scale would generally be associated with and adequate amount of food intake, regardless of origin, either animal or vegetable. The global increase in LE from 1960 to 2000 was associated with the increase in vegetable and animal food intake (Figure 6). Thus, the multivariate analyses shown in Figure 5 and Figure 6 clearly show that richer countries have higher LE associated with a richer and more abundant food intake and with other variables associated with life quality than poorer countries. However, at the same time, richer countries have higher incidence of cancer due to their higher intake of food sources coming from terrestrial animals and associated with the older age of their population. In contrast, poor countries that have had an improvement of their economic conditions have increased their LE through an increase in the quantity of food sources from both animals and plants.

## 5. Conclusions

Our analyses thus yielded four generally consistent conclusions after conducting deep Bayesian analyses of country-level data where the relationships between different diet sources and cancer prevalence were analyzed separately from other accompanying variables such as the mean age, the GDP, or the development stage of each country. First, the excessive intake of terrestrial animal food, especially the levels of protein, N and P, in developed countries was associated with higher prevalence of cancer whereas equivalent intake from vegetables was associated with lower prevalence. Residual confounding factors associated with the lifestyle of countries eating more or less animal and vegetable food could be masking these relationships, but we took into account the GDP, the mean age, the life expectancy, and the HDI, strongly linked to the lifestyle of their citizens, and yet these relationships still held. Second, no consistent relationships were found for N:P food ratio, thus falsifying the growth rate hypothesis of enhancement of malignant neoplasm prevalence. Third, the consumption of alcoholic beverages strongly correlated with the prevalence and mortality of malignant neoplasms, confirming earlier research. Fourth, the fight against famine is the most important task for improving health and increasing LE in underdeveloped countries.

## Supplementary Materials

The following are available online at https://www.mdpi.com/1660-4601/17/19/7240/s1, Figure S1: PCA with mean averages of country expectancy at birth (LE) (black), different socioeconomic variables (MA = mean age, HDI = human development index, GDP = gross domestic product per capita) (yellow) and food sources (t = total, N = nitrogen intake, P = phosphorus intake, prot = protein, and Kcal = kilocalories per capita intake) from terrestrial animal sources (ta) (red), vegetables (v) (green), and aquatic animal (aa) (blue) in the period 1960–2010; Table S1: Clustering, acronyms, and information sources for each explanatory variable used in the models; Table S2: Posterior median impacts for average cancer prevalence, 1998–2010. Table S3: Posterior median impacts for average cancer mortality, 1960–2010. Table S4: Posterior median impacts for average life expectancy, 1960–2010. Table S5: Country bivariate relationships between national prevalence of malignant neoplasms of the colon, prostate, breast, cervix, and lung and various traits of annual per capita intake during the same period (period 1998–2010). The bold type indicates statistical significance (*p* < 0.01). Table S6: Best linear models accounting for prevalence (period 1998–2010) from malignant neoplasms (total (TN), colon (CN), cervix (CEN), breast (BN), prostate (PN), and lung (LN) neoplasms as functions of national per capita wealth (using GDP), the human development index (HDI), mean age of the population (MA), life expectancy at birth (LE), and mean per capita intake of food from different sources. Results are provided for standardized variables. Table S7: Relationships within countries between national total mortality from malignant neoplasms of the colon, prostate, breast, cervix, and lung (period 1960–2010) and various traits of annual per capita intake during the same period. The bold type indicates statistical significance (*p* < 0.01). Table S8: Best linear models accounting for mortality from malignant neoplasms (total (TN), colon (CN), cervix (CEN), breast (BN), prostate (PN), and lung (LN) neoplasms as functions of national per capita wealth (using GDP), the human development index (HDI), mean age of the population (MA), life expectancy at birth (LE), and mean per capita intake of food from different sources. Table S9: Country relationships between national life expectancy at birth (period 1960–2010) and various traits of annual per capita intake during the same period. The bold type indicates statistical significance (*p* < 0.01).

## Abbreviations

LE, life expectancy; WHO, World Health Organization; UN, United Nations; ICD, International Classification of Diseases; OECD, Organization for Economic Co-operation and Development; FAO, Food and Agriculture Organization of the United Nations; GDP, gross domestic product; HDI, human development index; MA, population mean age.
