# Supplementary material for: Country-Level Relationships of the Human Intake of N and P, Animal and Vegetable Food, and Alcoholic Beverages with Cancer and Life Expectancy
Source: Int J Environ Res Public Health. 2020 Oct 3;17(19):7240. doi: 10.3390/ijerph17197240 (PMC7579602; doi:10.3390/ijerph17197240)
Supplement: Supplementary file 1 [file ijerph-17-07240-s001.pdf]

Supplementary material: Country level relationships of the human intake of N and P, animal and vegetable food and alcoholic beverages with cancer and life expectancy

Table S1. Clustering, acronyms and information sources for each explanatory variable used in the models.

Cluster	Variable	Acronym	Resource
1	Log GDP per capita	GDP	World bank, 2019b
	Human development index	HDI	United Nations Development Programme, 2019
	Median age of population	MA	WHO, 2019
	Life expectancy at birth	LE	World bank, 2019a
	Total terrestrial animals	Tta	FAO, FAOSTAT 2019; Food composition databse for biodiversity 2017; USDA 2016; DTU Fodevareinstituttet 2016
	Kcal terrestrial animals (% total	Kcalta %	FAO, FAOSTAT 2019; Food composition databse for biodiversity 2017; USDA 2016; DTU Fodevareinstituttet 2016
	P terrestrial animals	Pta	FAO, FAOSTAT 2019; Food composition databse for biodiversity 2017; USDA 2016; DTU Fodevareinstituttet 2016
	Protein terrestrial animals	Protta	FAO, FAOSTAT 2019; Food composition databse for biodiversity 2017; USDA 2016; DTU Fodevareinstituttet 2016
	Total animal/vegetable	Tav	FAO, FAOSTAT 2019; Food composition databse for biodiversity 2017; USDA 2016; DTU Fodevareinstituttet 2016
	Kcal animal/vegetable	Kcalav	FAO, FAOSTAT 2019; Food composition databse for biodiversity 2017; USDA 2016; DTU Fodevareinstituttet 2016
	P animal/vegetable	Pav	FAO, FAOSTAT 2019; Food composition databse for biodiversity 2017; USDA 2016; DTU Fodevareinstituttet 2016
	Protein animal/vegetable	Protav	FAO, FAOSTAT 2019; Food composition databse for biodiversity 2017; USDA 2016; DTU Fodevareinstituttet 2016
	N terrestrial animals	Nta	FAO, FAOSTAT 2019; Food composition databse for biodiversity 2017; USDA 2016; DTU Fodevareinstituttet 2016
	N animal/vegetable	Nav	FAO, FAOSTAT 2019; Food composition databse for biodiversity 2017; USDA 2016; DTU Fodevareinstituttet 2016
2	Total alcohol	Talc	FAO, FAOSTAT 2019; Food composition databse for biodiversity 2017; USDA 2016; DTU Fodevareinstituttet 2016
	Kcal alcohol (% total	Kcalalc %	FAO, FAOSTAT 2019; Food composition databse for biodiversity 2017; USDA 2016; DTU Fodevareinstituttet 2016
	P alcohol	Palc	FAO, FAOSTAT 2019; Food composition databse for biodiversity 2017; USDA 2016; DTU Fodevareinstituttet 2016
	Protein alcohol	Protalc	FAO, FAOSTAT 2019; Food composition databse for biodiversity 2017; USDA 2016; DTU Fodevareinstituttet 2016
3	Total vegetable	Tv	FAO, FAOSTAT 2019; Food composition databse for biodiversity 2017; USDA 2016; DTU Fodevareinstituttet 2016
	P vegetable	Pv	FAO, FAOSTAT 2019; Food composition databse for biodiversity 2017; USDA 2016; DTU Fodevareinstituttet 2016
	Protein vegetable	Protv	FAO, FAOSTAT 2019; Food composition databse for biodiversity 2017; USDA 2016; DTU Fodevareinstituttet 2016
	N vegetable	Nv	FAO, FAOSTAT 2019; Food composition databse for biodiversity 2017; USDA 2016; DTU Fodevareinstituttet 2016
4	N:P alcohol	NPalc	FAO, FAOSTAT 2019; Food composition databse for biodiversity 2017; USDA 2016; DTU Fodevareinstituttet 2016
	N:P aquatic animals	NPaa	FAO, FAOSTAT 2019; Food composition databse for biodiversity 2017; USDA 2016; DTU Fodevareinstituttet 2016
	N:P vegetable	NPv	FAO, FAOSTAT 2019; Food composition databse for biodiversity 2017; USDA 2016; DTU Fodevareinstituttet 2016
	N:P terrestrial animals	NPta	FAO, FAOSTAT 2019; Food composition databse for biodiversity 2017; USDA 2016; DTU Fodevareinstituttet 2016
	N alcohol	Nalc	FAO, FAOSTAT 2019; Food composition databse for biodiversity 2017; USDA 2016; DTU Fodevareinstituttet 2016
	Kcal vegetable (% total	Kcalv %	FAO, FAOSTAT 2019; Food composition databse for biodiversity 2017; USDA 2016; DTU Fodevareinstituttet 2016
5	Total aquatic animals	Taa	FAO, FAOSTAT 2019; Food composition databse for biodiversity 2017; USDA 2016; DTU Fodevareinstituttet 2016
	Kcal aquatic animals (% total	kcalaa %	FAO, FAOSTAT 2019; Food composition databse for biodiversity 2017; USDA 2016; DTU Fodevareinstituttet 2016,
	P aquatic animals	Paa	FAO, FAOSTAT 2019; Food composition databse for biodiversity 2017; USDA 2016; DTU Fodevareinstituttet 2016
	Protein aquatic animals	Paa	FAO, FAOSTAT 2019; Food composition databse for biodiversity 2017; USDA 2016; DTU Fodevareinstituttet 2016
	N aquatic animals	Naa	FAO, FAOSTAT 2019; Food composition databse for biodiversity 2017; USDA 2016; DTU Fodevareinstituttet 2016

Empirical Bayesian Framework

In this section we briefly summarize the key features of the empirical framework used. We assume that a scalar response variable y_i, (that contains – depending on the model - the standardized prevalence of cancer, standardized cancer mortality and life expectancy at birth, respectively) measured for country $i = 1, \dots, N$ arises from the following model

$$y_i = X_i' R' \beta + \varepsilon_i,$$

where X_i is a K −dimensional vector of fundamental factors that include income and age structure determinants, as well as observations of total, kilocaloric, phosphorous and protein intake from vegetable, alcoholic, terrestrial/aquatic animal sources, as well as an intercept term. R is a K by P rotation matrix, containing the three eigenvectors associated with the highest eigenvalues for each of the 5 cluster of covariates. β is a vector of regression coefficients of dimension $P \times 1$ and $\varepsilon_i \sim N(0, \sigma^2)$ is a Gaussian shock with variance σ^2.

This model can be easily estimated using maximum likelihood estimation. However, since one of the goals of this study is to analyze the driving forces that determine cancer prevalence rates across countries, we need a more flexible approach that allows to a.) assess uncertainty with respect to the underlying structural model and b.) enables robust estimation if the number of observations is small relative to the number of covariates K. The Bayesian approach allows, through flexible prior specifications, to control for model uncertainty and this entails estimating large models with only a moderate number of observations.

To set the stage, we assume that each element of β, β_j, arises from a mixture of Gaussians distribution. This prior, labeled the stochastic search variable selection (SSVS) prior (see George & McCulloch, 1993; 1997), is given by:

$$\beta_j | \delta_j \sim N(0, \tau_1^2)\delta_j + N(0, \tau_0^2)(1 - \delta_j),$$

whereby $\tau_1^2 \gg \tau_0^2$ denote prior scaling parameters, where τ_0^2 is specified to be close to zero and δ_j denotes an indicator variable that follows a Bernoulli distribution with prior inclusion probability p_0. In the empirical application, $\tau_1^2 = 10^2$ and $\tau_0^2 = 10^{-4}$ while $p_0 = 1/2$. This specification implies that if $\delta_j = 1$, a Gaussian prior with a larger prior variance is used for β_j with little weight attached to the prior information (i.e. exclusion of the corresponding element in X_i). This component of the mixture distribution is commonly referred to as the 'slab' distribution. By contrast, if $\delta_j = 0$, the prior variance is close to zero and the corresponding element in β_j is pushed to zero. We refer to this component as the 'spike' distribution. The δ_j can be used to infer what covariates determine cancer prevalence rates across regimes.

The remaining priors are standard in the literature. On σ^2, we use an inverted Gamma prior specified to be weakly informative while we use a Gaussian prior with zero mean and a large prior variance on γ.

Model estimation is carried out using a Markov chain Monte Carlo (MCMC) algorithm. This algorithm cycles between full conditional posterior distributions, iteratively sampling β_j from a Gaussian posterior density, σ^2 from an inverse Gamma posterior distribution, the indicators δ_j from a Bernoulli distribution. The posterior moments of all quantities except γ take standard forms and are, for the sake of brevity, not repeated here.

Table S2. Posterior median impacts for average cancer prevalence, 1998-2010.

Variable	Malignant neoplasms			Breast			Cervix			Colon			Lung			Prostate		
	Median	Std. Dev.	Sign	Median	Std. Dev.	Sign	Median	Std. Dev.	Sign	Median	Std. Dev.	Sign	Median	Std. Dev.	Sign	Median	Std. Dev.	Sign
Human development index	0.020	0.011	0.916	0.004	0.004	0.883	**-0.005**	0.001	1.000	**0.008**	0.002	1.000	**0.005**	0.002	0.989	-0.004	0.004	0.876
Kcal alcohol (% total)	0.005	0.705	0.521	-0.007	1.484	0.527	0.020	0.931	0.575	0.007	0.442	0.528	0.004	0.376	0.519	-0.001	0.857	0.504
Kcal animal/vegetable	0.016	0.009	0.925	0.005	0.003	0.959	**-0.003**	0.001	0.999	**0.006**	0.001	1.000	0.003	0.002	0.967	-0.001	0.002	0.774
Kcal aquatic animals (% total)	0.002	0.885	0.508	-0.003	0.702	0.513	0.118	0.751	0.728	-0.004	0.467	0.520	-0.001	0.459	0.504	-0.002	1.013	0.509
Kcal terrestrial animals (% total)	0.513	0.288	0.926	0.111	0.094	0.870	**-0.138**	0.033	1.000	**0.214**	0.046	1.000	**0.128**	0.056	0.990	-0.106	0.106	0.870
Kcal vegetable (% total)	-0.020	0.402	0.569	-0.004	0.456	0.504	-0.101	0.195	0.706	0.008	0.248	0.513	-0.169	0.292	0.720	-0.051	0.516	0.569
Life expectance at birth	1.450	0.821	0.913	0.298	0.269	0.858	**-0.396**	0.093	1.000	**0.613**	0.131	1.000	**0.370**	0.160	0.990	-0.325	0.307	0.883
Log GDP per capita	0.189	0.107	0.918	0.060	0.031	0.964	**-0.040**	0.011	0.999	**0.070**	0.015	1.000	0.033	0.019	0.963	-0.007	0.030	0.732
Median age of population	0.943	0.553	0.901	-0.196	0.358	0.696	**-0.478**	0.117	1.000	**0.576**	0.173	0.999	**0.515**	0.212	0.992	-0.775	0.583	0.900
N alcohol	0.001	0.030	0.557	-0.002	0.026	0.591	0.005	0.021	0.894	0.004	0.010	0.777	-0.002	0.019	0.651	0.003	0.017	0.663
N animal/vegetable	-0.001	0.945	0.505	-0.003	0.875	0.511	-0.111	1.079	0.744	-0.023	0.934	0.583	0.000	0.735	0.500	-0.001	0.812	0.504
N aquatic animals	0.000	0.049	0.507	-0.001	0.040	0.538	0.008	0.048	0.761	-0.002	0.025	0.666	-0.001	0.025	0.602	0.002	0.054	0.637
N terrestrial animals	0.155	0.087	0.928	**0.068**	0.027	0.992	**-0.022**	0.010	0.982	**0.049**	0.013	1.000	0.014	0.016	0.801	0.018	0.030	0.738
N vegetable	-0.005	0.039	0.717	-0.023	0.017	0.919	-0.005	0.006	0.813	-0.004	0.008	0.670	0.003	0.010	0.635	-0.036	0.027	0.914
N:P alcohol	0.025	0.456	0.583	-0.066	0.312	0.587	0.194	0.110	0.963	0.178	0.160	0.873	-0.116	0.192	0.732	0.138	0.414	0.680
N:P aquatic animals	0.000	0.013	0.545	0.000	0.013	0.527	-0.002	0.010	0.763	0.000	0.005	0.555	-0.001	0.009	0.606	-0.001	0.008	0.579
N:P terrestrial animals	0.001	0.854	0.506	0.006	0.785	0.523	-0.024	0.660	0.584	0.003	0.283	0.508	0.003	0.564	0.513	0.001	0.422	0.502
N:P vegetable	0.030	0.017	0.913	**0.016**	0.006	0.995	-0.002	0.002	0.866	**0.008**	0.003	0.996	0.000	0.004	0.549	0.007	0.008	0.797
P alcohol	0.008	0.959	0.533	-0.004	0.978	0.518	0.009	0.656	0.534	-0.001	0.750	0.505	0.000	0.830	0.500	0.000	0.994	0.502
P animal/vegetable	-0.001	0.888	0.503	-0.003	0.648	0.514	-0.091	0.934	0.721	-0.012	0.859	0.545	0.000	0.769	0.501	-0.002	0.799	0.507
P aquatic animals	0.002	0.560	0.510	-0.005	0.415	0.531	0.073	0.254	0.746	-0.018	0.304	0.619	-0.011	0.297	0.579	0.018	0.656	0.614
P terrestrial animals	0.545	0.300	0.959	**0.288**	0.108	0.995	-0.051	0.039	0.900	**0.149**	0.053	0.997	0.012	0.066	0.576	0.135	0.143	0.833
P vegetable	-0.093	0.635	0.695	-0.295	0.373	0.792	-0.187	0.162	0.879	-0.015	0.210	0.532	0.161	0.260	0.760	-0.592	0.455	0.912
Protein alcohol	0.002	0.085	0.569	-0.001	0.145	0.520	0.002	0.092	0.575	0.001	0.059	0.545	0.001	0.058	0.529	0.000	0.096	0.500
Protein animal/vegetable	-0.001	0.970	0.504	-0.002	0.919	0.509	-0.103	1.014	0.723	-0.016	0.977	0.560	0.001	0.820	0.504	-0.002	0.897	0.508
Protein aquatic animals	0.000	1.042	0.503	-0.008	1.184	0.531	0.160	2.307	0.764	-0.009	0.357	0.534	-0.006	0.373	0.524	0.007	0.823	0.527
Protein terrestrial animals	1.784	1.016	0.909	**0.984**	0.380	0.995	-0.133	0.138	0.835	**0.471**	0.187	0.994	0.010	0.230	0.518	0.475	0.526	0.799
Protein vegetable	-0.095	0.566	0.705	-0.393	0.350	0.924	0.024	0.157	0.563	-0.087	0.207	0.679	-0.050	0.255	0.589	-0.487	0.411	0.890
Total alcohol	**0.485**	0.138	1.000	0.020	0.062	0.631	**0.075**	0.025	0.984	**0.083**	0.031	0.994	**0.084**	0.037	0.990	0.019	0.085	0.591
Total animal/vegetable	0.000	0.439	0.503	-0.001	0.310	0.510	-0.047	0.459	0.725	-0.007	0.421	0.556	-0.001	0.379	0.507	0.000	0.396	0.501
Total aquatic animals	0.008	0.368	0.510	-0.008	0.209	0.530	-0.050	0.311	0.673	-0.093	0.099	0.936	-0.052	0.103	0.778	0.084	0.232	0.725
Total terrestrial animals	-0.156	0.179	0.730	-0.043	0.050	0.807	0.014	0.020	0.783	**-0.071**	0.025	0.997	-0.032	0.031	0.859	0.081	0.066	0.912
Total vegetable	0.003	0.093	0.515	0.014	0.043	0.628	0.002	0.016	0.560	-0.007	0.022	0.624	0.012	0.027	0.681	-0.004	0.064	0.525

Note: Estimates in bold are statistically significant with a 95% confidence interval. "Sign" denotes the posterior sign certainty of a covariate in the model.
The coefficients in the Bayesian models were interpreted using the sign certainty of each covariate. If the sign certainty is above 97.5 the coefficient is interpreted as significant..
The "median" column contains the estimates of the posterior coefficient, which describes the median increase in the dependent variable (e.g. cancer prevalence) in response to a one-unit increase in the explanatory variable (e.g. N/P intake).
The "Std. Deb." column contains the corresponding posterior standard deviations for the coefficients.

Table S3. Posterior median impacts for average cancer mortality, 1960-2010.

Variable	Malignant neoplasms			Breast			Cervix			Colon			Lung			Prostate		
	Median	Std. Dev.	Sign	Median	Std. Dev.	Sign	Median	Std. Dev.	Sign	Median	Std. Dev.	Sign	Median	Std. Dev.	Sign	Median	Std. Dev.	Sign
Human development index	**0.015**	0.005	0.998	**0.003**	0.001	0.998	**-0.003**	0.001	0.998	**0.002**	0.001	0.999	**0.003**	0.001	0.988	-0.001	0.001	0.827
Kcal alcohol (% total)	0.007	1.264	0.527	0.000	0.304	0.501	0.051	0.989	0.649	0.831	1.135	0.807	0.048	1.976	0.644	0.013	0.555	0.546
Kcal animal/vegetable	**0.009**	0.003	0.999	**0.001**	0.001	0.997	**-0.002**	0.000	1.000	**0.001**	0.000	0.999	**0.002**	0.001	0.999	-0.001	0.001	0.859
Kcal aquatic animals (% total)	-0.005	0.547	0.528	-0.002	0.524	0.507	0.016	0.401	0.569	0.005	0.306	0.522	-0.010	0.432	0.548	0.012	0.477	0.559
Kcal terrestrial animals (% total)	**0.516**	0.127	1.000	**0.053**	0.024	0.987	**-0.084**	0.022	1.000	**0.053**	0.017	0.999	**0.137**	0.036	1.000	-0.031	0.028	0.867
Kcal vegetable (% total)	**-1.352**	0.695	0.982	-0.241	0.130	0.966	**-0.361**	0.122	0.998	-0.187	0.095	0.974	-0.372	0.198	0.970	-0.298	0.158	0.968
Life expectance at birth	**1.178**	0.328	1.000	**0.171**	0.060	0.996	**-0.194**	0.056	1.000	**0.142**	0.043	0.999	**0.287**	0.091	0.999	-0.078	0.073	0.863
Log GDP per capita	0.092	0.056	0.949	**0.030**	0.011	0.997	-0.017	0.010	0.948	**0.018**	0.008	0.988	0.014	0.016	0.807	-0.008	0.013	0.748
Median age of population	**2.030**	0.491	1.000	0.146	0.093	0.938	**-0.322**	0.088	1.000	**0.181**	0.067	0.996	**0.569**	0.142	1.000	-0.113	0.111	0.846
N alcohol	0.007	0.044	0.873	-0.018	0.011	0.934	**-0.021**	0.010	0.976	-0.005	0.008	0.768	-0.001	0.016	0.596	-0.001	0.010	0.589
N animal/vegetable	0.004	0.021	0.573	**0.009**	0.004	0.988	-0.002	0.004	0.653	0.004	0.003	0.915	-0.003	0.006	0.703	-0.002	0.005	0.630
N aquatic animals	-0.006	0.025	0.886	-0.002	0.023	0.670	0.002	0.021	0.640	-0.001	0.014	0.558	-0.003	0.021	0.744	0.002	0.025	0.672
N terrestrial animals	0.017	0.052	0.631	**0.024**	0.010	0.990	-0.005	0.010	0.701	0.012	0.008	0.927	-0.006	0.016	0.659	-0.004	0.012	0.644
N vegetable	0.022	0.021	0.861	-0.004	0.004	0.842	-0.005	0.004	0.893	0.003	0.003	0.823	0.009	0.006	0.943	**-0.011**	0.005	0.989
N:P alcohol	**0.906**	0.268	0.999	-0.056	0.051	0.873	0.016	0.052	0.622	-0.009	0.045	0.582	0.069	0.088	0.776	-0.005	0.063	0.533
N:P aquatic animals	-0.006	0.074	0.742	0.028	0.019	0.908	0.034	0.017	0.969	0.008	0.013	0.736	0.002	0.027	0.563	0.000	0.018	0.511
N:P terrestrial animals	-0.032	2.537	0.618	-1.033	0.651	0.937	**-1.270**	0.583	0.982	-0.339	0.433	0.791	-0.129	0.916	0.742	-0.089	0.604	0.679
N:P vegetable	-0.023	0.645	0.746	0.262	0.166	0.936	**0.320**	0.148	0.981	0.085	0.110	0.786	0.028	0.233	0.700	0.020	0.154	0.653
P alcohol	0.004	0.184	0.587	0.001	0.133	0.514	0.006	0.157	0.615	0.080	0.147	0.813	0.008	0.202	0.636	0.001	0.136	0.533
P animal/vegetable	-0.002	0.008	0.610	**0.003**	0.002	0.981	0.000	0.001	0.509	0.001	0.001	0.860	-0.002	0.002	0.821	0.000	0.002	0.587
P aquatic animals	-0.052	0.419	0.774	-0.015	0.421	0.586	0.005	0.230	0.527	-0.009	0.226	0.555	-0.019	0.298	0.612	0.009	0.262	0.558
P terrestrial animals	-0.041	0.190	0.581	**0.080**	0.037	0.982	-0.005	0.036	0.557	0.032	0.029	0.867	-0.052	0.059	0.806	-0.011	0.044	0.598
P vegetable	0.365	0.591	0.832	0.051	0.132	0.656	-0.020	0.123	0.565	0.099	0.095	0.854	0.210	0.200	0.862	**-0.414**	0.157	0.996
Protein alcohol	0.005	1.110	0.517	0.004	0.954	0.516	0.023	0.934	0.579	0.104	0.787	0.759	0.035	0.700	0.607	0.003	0.927	0.508
Protein animal/vegetable	0.003	0.021	0.559	**0.010**	0.004	0.988	-0.001	0.004	0.643	0.004	0.003	0.912	-0.004	0.007	0.715	-0.002	0.005	0.627
Protein aquatic animals	-0.023	0.624	0.595	-0.023	0.505	0.591	0.024	0.650	0.589	0.002	0.381	0.509	-0.018	0.602	0.571	0.023	0.792	0.587
Protein terrestrial animals	-0.244	0.587	0.660	**0.239**	0.116	0.979	0.013	0.113	0.544	0.089	0.090	0.843	-0.191	0.183	0.845	-0.025	0.135	0.574
Protein vegetable	0.213	0.516	0.669	-0.141	0.108	0.902	-0.101	0.099	0.848	-0.014	0.077	0.573	0.065	0.160	0.656	0.050	0.126	0.659
Total alcohol	**0.207**	0.087	0.991	0.007	0.018	0.647	0.025	0.022	0.864	0.037	0.021	0.943	0.041	0.038	0.835	0.037	0.022	0.955
Total animal/vegetable	0.001	0.001	0.723	-0.001	0.000	0.975	0.000	0.000	0.664	0.000	0.000	0.813	0.001	0.000	0.872	0.000	0.000	0.544
Total aquatic animals	-0.241	0.174	0.965	-0.036	0.117	0.833	0.030	0.095	0.790	-0.030	0.071	0.843	-0.093	0.106	0.951	0.044	0.114	0.840
Total terrestrial animals	-0.010	0.082	0.552	**-0.034**	0.017	0.977	-0.021	0.016	0.915	-0.017	0.012	0.930	0.008	0.025	0.633	-0.001	0.019	0.520
Total vegetable	-0.032	0.054	0.741	0.005	0.011	0.672	0.011	0.011	0.847	-0.008	0.008	0.823	-0.006	0.017	0.644	**0.033**	0.014	0.994

Note: **E**stimates in bold are statistically significant with a 95% confidence interval. "Sign" denotes the posterior sign certainty of a covariate in the model.

Table S4. Posterior median impacts for average life expectancy, 1960-2010.

Variable	Life Expectancy		
	Median	Std. Dev.	P!=0
Kcal alcohol (% total)	1.834	0.635	0.970
Kcal animal/vegetable	0.001	0.002	0.633
Kcal aquatic animals (% total)	0.006	0.226	0.527
Kcal terrestrial animals (% total)	0.031	0.097	0.622
Kcal vegetable (% total)	-0.063	0.072	0.812
Log GDP per capita	0.004	0.009	0.680
N alcohol	-0.003	0.007	0.687
N animal/vegetable	0.003	0.007	0.666
N aquatic animals	0.002	0.009	0.738
N terrestrial animals	0.005	0.008	0.749
N vegetable	**-0.007**	0.003	0.988
N:P alcohol	-0.043	0.042	0.866
N:P aquatic animals	0.001	0.003	0.575
N:P terrestrial animals	-0.143	0.430	0.660
N:P vegetable	0.033	0.100	0.664
P alcohol	-12.141	4.116	0.973
P animal/vegetable	0.002	0.004	0.638
P aquatic animals	0.017	0.154	0.612
P terrestrial animals	0.014	0.028	0.704
P vegetable	-0.133	0.092	0.924
Protein alcohol	1.262	0.427	0.974
Protein animal/vegetable	0.003	0.006	0.687
Protein aquatic animals	0.019	0.245	0.573
Protein terrestrial animals	0.046	0.084	0.725
Protein vegetable	-0.057	0.080	0.767
Total alcohol	0.158	0.058	0.949
Total animal/vegetable	0.001	0.003	0.582
Total aquatic animals	**0.079**	0.051	0.979
Total terrestrial animals	0.005	0.012	0.648
Total vegetable	**0.022**	0.008	0.996

Note: Estimates in bold are statistically significant with a 95% confidence interval. "Sign" denotes the posterior sign certainty of a covariate in the model.

Table S5. Country bivariate relationships between national prevalence of malignant neoplasms of the colon, prostate, breast, cervix and lung and various traits of annual per capita intake during the same period (period 1998-2010). The bold type indicates statistical significance (*P*<0.01)

**Per capita national food intake (mean for 1990-2009)**	**National annual prevalence from malignant neoplasms (100000 inhabitants y^{-1}) (mean for 1990-2009)**					
	**Total**	**Breast**	**Cervix**	**Prostate**	**Colon**	**Lung**
Total vegetable intake (Tv)	*R*=0.32<br>*P*=0.031	*R*=0.16<br>*P*=0.28	*R*=-0.27<br>*P*=0.072	*R*=-0.032<br>*P*=0.83	*R*=0.080<br>*P*=0.60	*R*=0.39<br>*P*=0.007
Total N intake from vegetables (Nv)	*R*=0.16<br>*P*=0.29	*R*=0.050<br>*P*=0.74	*R*=-0.37<br>*P*=0.011	*R*=-0.20<br>*P*=0.18	*R*=0.022<br>*P*=0.88	*R*=0.28<br>*P*=0.057
Total P intake from vegetables (Pv)	*R*=0.068<br>*P*=0.66	*R*=0.094<br>*P*=0.54	*R*=-0.23<br>*P*=0.12	*R*=-0.35<br>*P*=0.017	*R*=-0.087<br>*P*=0.56	*R*=0.21<br>*P*=0.16
Total kilocalories from vegetables (Kcalv)	*R*=-0.22<br>*P*=0.15	*R*=-0.23<br>*P*=0.15	*R*=-0.17<br>*P*=0.27	*R*=-0.26<br>*P*=0.079	*R*=-0.18<br>*P*=0.23	*R*=-0.098<br>*P*=0.52
Total protein intake from vegetables (Protv)	*R*=-0.2<br>*P*=0.16	*R*=-0.24<br>*P*=0.11	*R*=-0.12<br>*P*=0.43	*R*=-0.32<br>*P*=0.031	*R*=-0.24<br>*P*=0.11	*R*=-0.092<br>*P*=0.54
Total intake of terrestrial animals (Tta)	***R*=0.66**<br>***P*<0.0001**	***R*=0.79**<br>***P*<0.0001**	***R*=-0.63**<br>***P*<0.0001**	***R*=0.70**<br>***P*<0.0001**	***R*=0.55**<br>***P*<0.0001**	***R*=0.57**<br>***P*<0.0001**

Total N intake from terrestrial animals (Nta)	**R=0.70 P<0.0001**	**R=0.81 P<0.0001**	**R=-0.66 P<0.0001**	**R=0.66 P<0.0001**	**R=0.63 P<0.0001**	**R=0.63 P<0.0001**
Total P intake from terrestrial animals (Pta)	**R=0.67 P<0.0001**	**R=0.80 P<0.0001**	**R=-0.65 P<0.0001**	**R=0.69 P<0.0001**	**R=0.57 P<0.0001**	**R=0.59 P<0.0001**
Total kilocalories from terrestrial animals (Kcalta)	**R=0.73 P<0.0001**	**R=0.82 P<0.0001**	**R=-0.61 P<0.0001**	**R=0.66 P<0.0001**	**R=0.65 P<0.0001**	**R=0.66 P<0.0001**
Total protein intake from terrestrial animals (Protta)	**R=0.68 P<0.0001**	**R=0.79 P<0.0001**	**R=-0.69 P<0.0001**	**R=0.61 P<0.0001**	**R=0.64 P<0.0001**	**R=0.61 P<0.0001**
Total intake of alcoholic beverages (Talc)	**R=0.65 P<0.0001**	**R=0.49 P<0.0001**	R=-0.069 P=0.65	**R=0.42 P=0.004**	**R=0.58 P<0.0001**	**R=0.58 P<0.0001**
Total N intake from alcoholic beverages (Nalc)	R=0.010 P=0.95	R=-0.24 P=0.11	**R=0.63 P<0.0001**	R=-0.11 P=0.46	R=-0.15 P=0.32	R=-0.14 P=0.35
Total P intake from alcoholic beverages (Palc)	**R=0.58 P<0.0001**	R=0.37 P=0.011	R=0.10 P=0.50	R=0.34 P=0.021	R=0.48 P=0.001	R=0.47 P=0.001
Total kilocalories from alcoholic beverages (Kcalalc)	R=0.40 P=0.005	R=0.24 P=0.10	R=-0.032 P=0.84	R=0.22 P=0.15	R=0.44 P=0.002	R=0.42 P=0.003
Total protein intake from alcoholic beverages (Protalc)	**R=0.55 P<0.0001**	R=0.37 P=0.011	R=-0.063 P=0.68	R=0.27 P=0.067	**R=0.56 P<0.0001**	**R=0.52 P<0.0001**
Total intake of aquatic animals (Taa)	R=0.26 P=0.087	R=0.23 P=0.13	R=-0.28 P=0.056	R=0.14 P=0.34	R=0.24 P=0.11	R=0.18 P=0.22
Total N intake from aquatic animals (Naa)	R=0.24 P=0.12	R=0.11 P=0.49	R=-0.039 P=0.80	R=0.077 P=0.61	R=0.16 P=0.30	R=0.11 P=0.47
Total P intake from aquatic animals (Paa)	R=0.25 P=0.097	R=0.22 P=0.14	R=-0.28 P=0.061	R=0.15 P=0.34	R=0.23 P=0.12	R=0.17 P=0.25
Total kilocalories from aquatic animals (Kcalaa)	R=-0.0095 P=0.95	R=-0.070 P=0.65	R=-0.20 P=0.18	R=-0.15 P=0.30	R=0.042 P=0.78	R=0.025 P=0.87
Total protein intake from aquatic animals (Protaa)	R=0.20 P=0.17	R=0.15 P=0.30	R=-0.23 P=0.12	R=0.084 P=0.58	R=0.21 P=0.16	R=0.15 P=0.32
Ratio of intake of animal/vegetable foods (Tav)	**R=0.56 P<0.0001**	**R=0.74 P<0.0001**	**R=-0.54 P<0.0001**	**R=0.74 P<0.0001**	**R=0.53 P<0.0001**	**R=0.41 P=0.005**
Ratio of N intake from animal/vegetable foods (Nav)	**R=0.61 P<0.0001**	**R=0.77 P<0.0001**	**R=-0.52 P<0.0001**	**R=0.75 P<0.0001**	**R=0.62 P<0.0001**	**R=0.47 P=0.001**
Ratio of P intake from animal/vegetable foods (Pav)	**R=0.58 P<0.0001**	**R=0.77 P<0.0001**	**R=-0.54 P<0.0001**	**R=0.77 P<0.0001**	**R=0.56 P<0.0001**	**R=0.43 P=0.003**
Ratio of protein intake from animal/vegetable foods (Protav)	**R=0.69 P<0.0001**	**R=0.78 P<0.0001**	**R=-0.58 P<0.0001**	**R=0.67 P<0.0001**	**R=0.66 P<0.0001**	**R=0.56 P<0.0001**
Ratio of kilocalorie intake from animal/vegetable foods (Kcalav)	**R=0.72 P<0.0001**	**R=0.82 P<0.0001**	**R=-0.50 P<0.0001**	**R=0.71 P<0.0001**	**R=0.67 P<0.0001**	**R=0.60 P<0.0001**
Ratio of intake of terrestrial animal/vegetable foods (Ttav)	R=0.17 P=0.26	R=0.19 P=0.21	R=-0.23 P=0.12	R=0.14 P=0.34	R=0.20 P=0.19	R=0.098 P=0.52
Ratio of N intake from terrestrial animal/vegetable foods (Ntav)	**R=0.60 P<0.0001**	**R=0.77 P<0.0001**	**R=-0.53 P<0.0001**	**R=0.75 P<0.0001**	**R=0.61 P<0.0001**	**R=0.47 P<0.0001**
Ratio of P intake from terrestrial animal/vegetable foods (Ptav)	**R=0.57 P<0.0001**	**R=0.76 P<0.0001**	**R=-0.52 P<0.0001**	**R=0.77 P<0.0001**	**R=0.54 P<0.0001**	**R=0.43 P=0.003**
Ratio of protein intake from terrestrial animal/vegetable foods (Prottav)	**R=0.68 P<0.0001**	**R=0.79 P<0.0001**	**R=-0.57 P<0.0001**	**R=0.68 P<0.0001**	**R=0.65 P<0.0001**	**R=0.56 P<0.0001**
Ratio of kilocalorie intake from terrestrial animal/vegetable foods (Kcaltav)	**R=0.72 P<0.0001**	**R=0.82 P<0.0001**	**R=-0.50 P<0.0001**	**R=0.71 P<0.0001**	**R=0.67 P<0.0001**	**R=0.59 P<0.0001**
N:P ratio of vegetable foods (NPv)	R=0.27 P=0.074	R=0.42 P=0.004	R=-0.42 P=0.004	R=0.39 P=0.007	R=0.32 P=0.028	R=0.23 P=0.12
N:P ratio of terrestrial animal foods (NPta)	R=-0.022 P=0.89	R=-0.15 P=0.306	R=0.030 P=0.84	R=-0.33 P=0.023	R=0.11 P=0.45	R=0.099 P=0.51
N:P ratio of aquatic animal foods (NPaa)	R=-0.11 P=0.48	R=-0.28 P=0.057	**R=0.71 P<0.0001**	R=-0.12 P=0.44	R=-0.16 P=0.29	R=-0.21 P=0.17

Table S6. Best linear models accounting for prevalence (period 1998-2010) from malignant neoplasms (total (TN), colon (CN), cervix (CEN), breast (BN), prostate (PN) and lung (LN) neoplasms as functions of national per capita wealth (using GDP), the human development index (HDI), mean age of the population (MA), Life Expectance at Birth (LE) and mean per capita intake of food from different sources. Results are provided for standardized variables.

Total prevalence of neoplasms for 1998-2010						
**Model**	**Statistical results of the model**	**Independent factor statistics**				
Model: TN.stan ~ HDI.stan + GDP.stan + LE.stan + AM.stan + **ttav.stan** + HDI.stan:GDP.stan + HDI.stan:LE.stan + GDP.stan:LE.stan + LE.stan:AM.stan + **AM.stan:ttav.stan**	R^2=0.72		Value	Std.Error	t-value	p-value
	P<0.00001	(intercept)	-0.1251285	0.1490982	-0.839236	0.4070
		HDI.stan	0.1618176	0.1602311	1.009.902	0.3195
		GDP.stan	0.3010823	0.1739377	1.730.978	0.0923
		LE.stan	0.2616746	0.2203495	1.187.543	0.2430
		AM.stan	0.3825523	0.1723857	2.219.165	0.0331
		**ttav.stan**	0.0347696	0.1084630	0.320567	0.7504
		HDI.stan:GDP.stan	0.6236354	0.2551696	2.444.003	0.0197
		HDI.stan:LE.stan	-0.3888262	0.1826878	-2.128.365	0.0404
		GDP.stan:LE.stan	-0.8664076	0.3118974	-2.777.861	0.0087
		LE.stan:AM.stan	0.9163548	0.2340509	3.915.194	0.0004
		**AM.stan:ttav.stan**	-0.4561334	0.1270703	-3.589.615	0.0010
Model:;TN.stan;~;GDP.stan;+;LE.stan;+;AM.stan;+;**Ntav.stan**;+;GDP.stan:LE.stan ;+;**GDP.stan:Ntav**.stan;+;LE.stan:AM.stan	R^2=0.66		Value	Std.Error	t-value	p-value
	P<0.00001	(intercept)	-0.2587012	0.1597618	-1.619.294	0.1137
		GDP.stan	0.1691061	0.1935359	0.873771	0.3877
		LE.stan	0.2674327	0.2261697	1.182.443	0.2444
		AM.stan	0.4742853	0.1677242	2.827.769	0.0074
		**Ntav.stan**	0.2851863	0.1489376	1.914.803	0.0631
		GDP.stan:LE.stan	-0.7006257	0.3058116	-2.291.037	0.0276
		**GDP.stan:Ntav.stan**	0.4356713	0.1600018	2.722.915	0.0097
		LE.stan:AM.stan	0.5369160	0.1548809	3.466.638	0.0013
Model:;TN.stan;~;GDP.stan;+;LE.stan;+;AM.stan;+;**Ptav.stan**;+;GDP.stan:LE.stan; +;**GDP.stan:Ptav.stan**;+;LE.stan:AM.stan	R^2=0.66		Value	Std.Error	t-value	p-value
	P<0.00001	(intercept)	-0.2329379	0.1534296	-1.518.207	0.1372
		GDP.stan	0.1894675	0.1895170	0.999739	0.3238
		LE.stan	0.2518151	0.2233287	1.127.554	0.2666
		AM.stan	0.4899288	0.1603541	3.055.293	0.0041
		**Ptav.stan**	0.2304006	0.1389042	1.658.701	0.1054
		GDP.stan:LE.stan	-0.7884585	0.3086890	-2.554.217	0.0148
		**GDP.stan:Ptav.stan**	0.4418255	0.1512324	2.921.500	0.0058
		LE.stan:AM.stan	0.5706709	0.1556246	3.666.972	0.0007

	R^2=0.71		Value	Std.Error	t-value	p-value
Model:;TN.stan;~;GDP.stan;+;LE.stan;+;AM.stan;+;**kcaltav.stan**;+;GDP.stan:LE.stan;+;**GDP.stan:kcaltav.stan**;+;LE.stan:AM.stan	$P<0.00001$	(intercept)	-0.1891234	0.1445357	-1.308.490	0.1986
		GDP.stan	0.1292479	0.1713599	0.754248	0.4553
		LE.stan	0.2257732	0.2057282	1.097.434	0.2794
		AM.stan	0.3882930	0.1775703	2.186.700	0.0350
		**kcaltav.stan**	0.4512881	0.1378277	3.274.292	0.0023
		GDP.stan:LE.stan	-0.7516556	0.2881297	-2.608.741	0.0129
		**GDP.stan:kcaltav.stan**	0.3263815	0.1575799	2.071.213	0.0452
		LE.stan:AM.stan	0.5838546	0.1454616	4.013.805	0.0003
	R^2=0.67		Value	Std.Error	t-value	p-value
Model:;TN.stan;~;GDP.stan;+;LE.stan;+;AM.stan;+;**prottav.stan**;+;GDP.stan:LE.stan;+;**GDP.stan:prottav.stan**;+;LE.stan:AM.stan	$P<0.00001$	(intercept)	-0.2254940	0.1586121	-1.421.670	0.1633
		GDP.stan	0.0684781	0.1979821	0.345880	0.7313
		LE.stan	0.2153611	0.2220915	0.969695	0.3383
		AM.stan	0.4404340	0.1663310	2.647.937	0.0117
		**prottav.stan**	0.4482353	0.1551924	2.888.256	0.0064
		GDP.stan:LE.stan	-0.6728305	0.3154461	-2.132.949	0.0394
		**GDP.stan:prottav.stan**	0.3253571	0.1618873	2.009.775	0.0516
		LE.stan:AM.stan	0.5454050	0.1587099	3.436.490	0.0014
	R^2=0.74		Value	Std.Error	t-value	p-value
Model:;BN.stan;~;HDI.stan;+;LE.stan;+;AM.stan;+;**ttav.stan**;+;HDI.stan:AM.stan;+;**HDI.stan:ttav.stan**;+;LE.stan:AM.stan;+;**LE.stan:ttav.stan;+;AM.stan:ttav.stan**	$P<0.00001$	(intercept)	-0.0877111	0.1058173	-0.828892	0.4126
		HDI.stan	0.9422807	0.1790094	5.263.861	0.0000
		LE.stan	0.3089427	0.1865327	1.656.239	0.1064
		AM.stan	0.0768891	0.1408651	0.545835	0.5885
		**ttav.stan**	-0.0546195	0.1344603	-0.406213	0.6870
		HDI.stan:AM.stan	-0.6171155	0.1643484	-3.754.923	0.0006
		**HDI.stan:ttav.stan**	14.865.263	0.4291565	3.463.833	0.0014
		LE.stan:AM.stan	0.7521249	0.1440494	5.221.300	0.0000
		**LE.stan:ttav.stan**	-0.8540175	0.2778746	-3.073.392	0.0040
		**AM.stan:ttav.stan**	-0.5891346	0.1676165	-3.514.776	0.0012
	R^2=0.80		Value	Std.Error	t-value	p-value
Model:;BN.stan;~;HDI.stan;+;LE.stan;+;AM.stan;+;**Ntav.stan**;+;HDI.stan:AM.stan;+;LE.stan:AM.stan;+;**AM.stan:Ntav.stan**	$P<0.00001$	(intercept)	-0.3142008	0.10178501	-3.086.907	0.0038
		HDI.stan	0.6082818	0.14075967	4.321.421	0.0001
		LE.stan	0.4124102	0.14940824	2.760.291	0.0088
		AM.stan	-0.0409536	0.12781136	-0.320423	0.7504
		**Ntav.stan**	0.4833802	0.11081844	4.361.912	0.0001
		HDI.stan:AM.stan	-0.5611261	0.13290886	-4.221.886	0.0001
		LE.stan:AM.stan	0.4053079	0.09996436	4.054.524	0.0002

		**AM.stan:Ntav.stan**	0.5198068	0.12880696	4.035.549	0.0003
Model:;BN.stan;~;HDI.stan;+;LE.stan;+;AM.stan;+;**Ptav.stan**;+;HDI.stan:AM.stan;+;**AM.stan:Ptav.stan**;+;LE.stan:AM.stan	R^2=0.80		Value	Std.Error	t-value	p-value
	P<0.00001	(intercept)	-0.2752186	0.09643197	-2.854.018	0.0070
		HDI.stan	0.5654125	0.13305217	4.249.555	0.0001
		LE.stan	0.3978351	0.14843876	2.680.129	0.0108
		AM.stan	0.0002984	0.12232622	0.002439	0.9981
		**Ptav.stan**	0.4617149	0.10201485	4.525.958	0.0001
		HDI.stan:AM.stan	-0.5073758	0.12309826	-4.121.714	0.0002
		**AM.stan:Ptav.stan**	0.4844317	0.11922413	4.063.202	0.0002
		LE.stan:AM.stan	0.3794567	0.09821910	3.863.370	0.0004
Model:;BN.stan;~;HDI.stan;+;LE.stan;+;AM.stan;+;**prottav.stan**;+;HDI.stan:AM.stan;+;**HDI.stan:prottav.stan**;+;LE.stan:AM.stan	R^2=0.77		Value	Std.Error	t-value	p-value
	P<0.00001	(intercept)	-0.2402186	0.1043541	-2.301.957	0.0269
		HDI.stan	0.6600737	0.1636963	4.032.308	0.0003
		LE.stan	0.2057114	0.1582000	1.300.325	0.2013
		AM.stan	-0.0147851	0.1321208	-0.111906	0.9115
		**prottav.stan**	0.3986242	0.1296467	3.074.695	0.0039
		HDI.stan:AM.stan	-0.5585089	0.1484598	-3.762.020	0.0006
		**HDI.stan:prottav.stan**	0.5353708	0.1645221	3.254.096	0.0024
		LE.stan:AM.stan	0.3144231	0.1070947	2.935.936	0.0056
;Model:;BN.stan;~;HDI.stan;+;GDP.stan;+;LE.stan;+;AM.stan;+;**kcaltav.stan**;+;HDI.stan:AM.stan;+;**GDP.stan:kcaltav.stan**;+;**LE.stan:kcaltav.stan**;+;**AM.stan:kcaltav.stan**	R^2=0.85		Value	Std.Error	t-value	p-value
	P<0.00001	(intercept)	-0.2756845	0.1095591	-2.516.307	0.0165
		HDI.stan	0.4195091	0.1175924	3.567.484	0.0010
		GDP.stan	0.2706731	0.1191543	2.271.618	0.0292
		LE.stan	0.4972958	0.1511546	3.289.981	0.0022
		AM.stan	-0.0487013	0.1324371	-0.367731	0.7152
		**kcaltav.stan**	0.4207296	0.1133369	3.712.204	0.0007
		HDI.stan:AM.stan	-0.4489550	0.1037337	-4.327.957	0.0001
		**GDP.stan:kcaltav.stan**	-0.4606323	0.1389655	-3.314.724	0.0021
		**LE.stan:kcaltav.stan**	0.4612517	0.1142084	4.038.686	0.0003
		**AM.stan:kcaltav.stan**	0.8262793	0.1815085	4.552.291	0.0001
;Model:;cervix.stan;~;HDI.stan;+;GDP.stan;+;LE.stan;+;**Ntav.stan**;+;HDI.stan:LE.stan;+;GDP.stan:LE.stan;+;**LE.stan:Ntav.stan**	R^2=0.84		Value	Std.Error	t-value	p-value
	P<0.00001	(intercept)	-0.3002406	0.09866942	-3.042.894	0.0042
		HDI.stan	-0.2752375	0.10797930	-2.548.984	0.0150
		GDP.stan	-0.5068968	0.12601723	-4.022.440	0.0003
		LE.stan	-0.0749195	0.15490034	-0.483662	0.6314
		**Ntav.stan**	0.2635880	0.10659793	2.472.730	0.0180

		HDI.stan:LE.stan	0.3804165	0.10002814	3.803.095	0.0005
		GDP.stan:LE.stan	0.6538295	0.19416365	3.367.414	0.0017
		**LE.stan:Ntav.stan**	-0.4840453	0.15383968	-3.146.427	0.0032
	R^2=0.84		Value	Std.Error	t-value	p-value
	P<0.00001	(intercept)	-0.1575695	0.1164389	-1.353.237	0.1847
		HDI.stan	-0.4733239	0.1543448	-3.066.665	0.0042
		GDP.stan	-0.3009803	0.1396090	-2.155.881	0.0380
		LE.stan	-0.2652341	0.1665256	-1.592.753	0.1202
Model:;cervix.stan;~;HDI.stan;+;GDP.stan;+;LE.stan;+;AM.stan;+;**Ptav.stan**;+;HDI.stan:LE.stan;+;HDI.stan:AM.stan;+;**HDI.stan:Ptav.stan**;+;GDP.stan:LE.stan;+;LE.stan:AM.stan		AM.stan	0.1476106	0.1229281	1.200.788	0.2379
		**Ptav.stan**	0.1988214	0.1050885	1.891.942	0.0668
		HDI.stan:LE.stan	0.3259991	0.1760197	1.852.061	0.0725
		HDI.stan:AM.stan	0.3385528	0.1607237	2.106.427	0.0424
		**HDI.stan:Ptav.stan**	-0.4676457	0.1504760	-3.107.777	0.0037
		GDP.stan:LE.stan	0.5089003	0.2039784	2.494.873	0.0175
		LE.stan:AM.stan	-0.3332563	0.1781053	-1.871.120	0.0697
	R^2=0.83		Value	Std.Error	t-value	p-value
	P<0.00001	(intercept)	-0.2618187	0.09918226	-2.639.773	0.0120
		HDI.stan	-0.2302104	0.10452354	-2.202.474	0.0338
		GDP.stan	-0.5118058	0.13115898	-3.902.179	0.0004
Model:;cervix.stan;~;HDI.stan;+;GDP.stan;+;LE.stan;+;**prottav.stan**;+;HDI.stan:LE.stan;+;GDP.stan:LE.stan;+;**LE.stan:prottav.stan**		LE.stan	-0.2285240	0.15820446	-1.444.485	0.1568
		**prottav.stan**	0.2967192	0.12245334	2.423.121	0.0203
		HDI.stan:LE.stan	0.3565570	0.09725301	3.666.282	0.0007
		GDP.stan:LE.stan	0.7557401	0.21610403	3.497.112	0.0012
		**LE.stan:prottav.stan**	-0.5804262	0.18312134	-3.169.626	0.0030
	R^2=0.83		Value	Std.Error	t-value	p-value
	P<0.00001	(intercept)	-0.0580418	0.1322898	-0.438747	0.6635
		HDI.stan	-0.5328013	0.1756033	-3.034.118	0.0045
		GDP.stan	-0.2924362	0.1316979	-2.220.508	0.0328
		LE.stan	-0.4964741	0.1668885	-2.974.885	0.0052
Model:;cervix.stan;~;HDI.stan;+;GDP.stan;+;LE.stan;+;AM.stan;+;**kcaltav.stan**;+;HDI.stan:LE.stan;+;**HDI.stan:kcaltav.stan**;+;GDP.stan:AM.stan;+;LE.stan:AM.stan		AM.stan	0.1994711	0.1484046	1.344.103	0.1873
		**kcaltav.stan**	0.3470911	0.1221047	2.842.569	0.0073
		HDI.stan:LE.stan	0.6579857	0.1831836	3.591.946	0.0010
		**HDI.stan:kcaltav.stan**	-0.5669015	0.1914562	-2.960.999	0.0054
		GDP.stan:AM.stan	0.4107791	0.1523566	2.696.169	0.0106
		LE.stan:AM.stan	-0.3363666	0.1804664	-1.863.874	0.0705
	R^2=0.64		Value	Std.Error	t-value	p-value

Model:;PN.stan;~;HDI.stan;+;GDP.stan;+;LE.stan;+;AM.stan;+;**ttav.stan**;+;HDI.stan:AM.stan;+;**HDI.stan:ttav.stan;+;GDP.stan:ttav.stan**;+;LE.stan:AM.stan;+;LE.stan:ttav.stan;+;**AM.stan:ttav.stan**	*P*<0.00001	(intercept)	-0.2226798	0.1456135	-1.529.252	0.1355
		HDI.stan	11.621.214	0.2414880	4.812.336	0.0000
		GDP.stan	0.1427893	0.1729947	0.825397	0.4149
		LE.stan	0.0614843	0.2352612	0.261345	0.7954
		AM.stan	-0.1588032	0.1802173	-0.881176	0.3844
		**ttav.stan**	-0.2259622	0.1686780	-1.339.607	0.1893
		HDI.stan:AM.stan	-0.7252059	0.2119163	-3.422.133	0.0016
		**HDI.stan:ttav.stan**	14.716.111	0.6148816	2.393.324	0.0224
		**GDP.stan:ttav.stan**	0.5315080	0.2972749	1.787.934	0.0827
		LE.stan:AM.stan	0.8396516	0.2230773	3.763.950	0.0006
		**LE.stan:ttav.stan**	-11.919.337	0.4045735	-2.946.149	0.0058
		**AM.stan:ttav.stan**	-0.4639907	0.2130512	-2.177.837	0.0365
Model:;PN.stan;~;HDI.stan;+;GDP.stan;+;LE.stan;+;AM.stan;+;**Ntav.stan**;+;HDI.stan:GDP.stan;+;HDI.stan:LE.stan;+;HDI.stan:AM.stan	R^2=0.72		Value	Std.Error	t-value	p-value
	P<0.00001	(intercept)	-0.2598320	0.1368095	-1.899.225	0.0654
		HDI.stan	0.3599831	0.1380922	2.606.832	0.0131
		GDP.stan	-0.3209879	0.1977814	-1.622.943	0.1131
		LE.stan	0.0820881	0.1744252	0.470621	0.6407
		AM.stan	-0.1074352	0.1659189	-0.647516	0.5213
		**Ntav.stan**	0.8457068	0.1394282	6.065.537	0.0000
		HDI.stan:GDP.stan	0.8705592	0.3232918	2.692.797	0.0106
		HDI.stan:LE.stan	0.5525969	0.1666507	3.315.899	0.0021
		HDI.stan:AM.stan	-0.9370450	0.2764120	-3.390.030	0.0017
Model:;PN.stan;~;HDI.stan;+;GDP.stan;+;LE.stan;+;AM.stan;+;**Ptav.stan**;+;HDI.stan:GDP.stan;+;HDI.stan:LE.stan;+;HDI.stan:AM.stan	R^2=0.75		Value	Std.Error	t-value	p-value
	P<0.00001	(intercept)	-0.2087323	0.1301812	-1.603.397	0.1174
		HDI.stan	0.3804819	0.1313600	2.896.483	0.0063
		GDP.stan	-0.2991483	0.1876446	-1.594.228	0.1194
		LE.stan	0.0408219	0.1669437	0.244525	0.8082
		AM.stan	-0.0570666	0.1579246	-0.361353	0.7199
		**Ptav.stan**	0.8290821	0.1259203	6.584.180	0.0000
		HDI.stan:GDP.stan	0.7094303	0.3071208	2.309.939	0.0266
		HDI.stan:LE.stan	0.5878519	0.1603610	3.665.804	0.0008
		HDI.stan:AM.stan	-0.9128397	0.2643600	-3.453.018	0.0014
Model:;PN.stan;~;HDI.stan;+;LE.stan;+;**prottav.stan**;+;**LE.stan:prottav.stan**	R^2=0.55		Value	Std.Error	t-value	p-value
	P<0.00001	(intercept)	-0.1692783	0.1334147	-1.268.813	0.2117
		HDI.stan	0.3033815	0.1446926	2.096.731	0.0422
		LE.stan	0.1383317	0.2225896	0.621465	0.5377
		**prottav.stan**	0.5509343	0.1574834	3.498.364	0.0011

		**LE.stan:prottav.stan**	0.2698676	0.1333680	2.023.481	0.0496
	R^2=0.67		Value	Std.Error	t-value	p-value
	P<0.00001	(intercept)	-0.2420906	0.1640377	-1.475.823	0.1485
		HDI.stan	0.5464055	0.1902629	2.871.844	0.0067
		GDP.stan	-0.2343182	0.2184664	-1.072.559	0.2904
		LE.stan	0.0591964	0.1891334	0.312987	0.7560
Model:;PN.stan;~;HDI.stan;+;GDP.stan;+;LE.stan;+;AM.stan;**+;kcaltav.stan**;+;HDI.stan:LE.stan;+;HDI.stan:AM.stan;+;GDP.stan:AM.stan		AM.stan	-0.1579067	0.1931901	-0.817365	0.4190
		**kcaltav.stan**	0.8143203	0.1589870	5.121.929	0.0000
		HDI.stan:LE.stan	0.6486190	0.2027379	3.199.298	0.0028
		HDI.stan:AM.stan	-0.8862007	0.2872351	-3.085.280	0.0038
		GDP.stan:AM.stan	0.5522894	0.2629355	2.100.475	0.0426
	R^2=0.72		Value	Std.Error	t-value	p-value
	P<0.00001	(intercept)	-0.0918088	0.1177077	-0.779973	0.4404
		HDI.stan	0.3193803	0.1579221	2.022.391	0.0504
		LE.stan	0.2928968	0.1738038	1.685.215	0.1004
		AM.stan	0.4384025	0.1476019	2.970.168	0.0052
;Model:;CN.stan;~;HDI.stan;+;LE.stan;+;AM.stan;+;**Ntav.stan**;+;HDI.stan:LE.stan;+;HDI.stan:AM.stan;+;**HDI.stan:Ntav.stan**;+;**AM.stan:Ntav.stan**		**Ntav.stan**	0.1685375	0.1398972	1.204.724	0.2360
		HDI.stan:LE.stan	0.5950863	0.1573722	3.781.394	0.0006
		HDI.stan:AM.stan	-0.6269269	0.2109114	-2.972.465	0.0052
		**HDI.stan:Ntav.stan**	-0.6166210	0.2925390	-2.107.825	0.0419
		**AM.stan:Ntav.stan**	0.7006359	0.2548419	2.749.296	0.0092
	R^2=0.72		Value	Std.Error	t-value	p-value
	P<0.00001	(intercept)	-0.0319212	0.1137691	-0.280579	0.7806
		HDI.stan	0.3292686	0.1462342	2.251.653	0.0304
		LE.stan	0.2606878	0.1715191	1.519.876	0.1370
		AM.stan	0.4572727	0.1425986	3.206.713	0.0028
Model:;CN.stan;~;HDI.stan;+;LE.stan;+;AM.stan;+;**Ptav.sta**n;+;HDI.stan:LE.stan;+;HDI.stan:AM.stan;+;HDI.stan:Ptav.stan;+;**AM.stan:Ptav.stan**		**Ptav.stan**	0.1112400	0.1274104	0.873084	0.3882
		HDI.stan:LE.stan	0.5883029	0.1539173	3.822.201	0.0005
		HDI.stan:AM.stan	-0.5835045	0.2005051	-2.910.173	0.0061
		**HDI.stan:Ptav.stan**	-0.5836144	0.2379337	-2.452.845	0.0190
		**AM.stan:Ptav.stan**	0.5807744	0.2014921	2.882.368	0.0065
	R^2=0.80		Value	Std.Error	t-value	p-value
Model:;CN.stan;~;HDI.stan;+;GDP.stan;+;LE.stan;+;AM.stan;+;**prottav.stan**;+;HDI.stan:LE.stan;+;HDI.stan:AM.stan;+;GDP.stan:LE.stan;+;GDP.stan:AM.stan;+;**LE.stan:prottav.stan**;+;**AM.stan:prottav.stan**	*P*<0.00001	(intercept)	-0.2014213	0.1433484	-1.405.117	0.1691
		HDI.stan	0.2575596	0.1635854	1.574.465	0.1246
		GDP.stan	-0.4998168	0.1846713	-2.706.521	0.0106
		LE.stan	0.4333568	0.1898279	2.282.894	0.0288

		AM.stan	0.2885890	0.1626367	1.774.440	0.0849
		**prottav.stan**	0.5458596	0.1526162	3.576.682	0.0011
		HDI.stan:LE.stan	0.6726797	0.2273727	2.958.490	0.0056
		HDI.stan:AM.stan	-0.6977315	0.2704661	-2.579.738	0.0144
		GDP.stan:LE.stan	13.588.176	0.3256786	4.172.265	0.0002
		GDP.stan:AM.stan	-0.5149015	0.2582874	-1.993.522	0.0543
		**LE.stan:prottav.stan**	-11.345.341	0.2957170	-3.836.553	0.0005
		**AM.stan:prottav.stan**	0.7265534	0.2019432	3.597.811	0.0010
Model:;CN.stan;~;HDI.stan;+;GDP.stan;+;LE.stan;+;AM.stan;+;**kcaltav.stan**;+;HDI.stan:LE.stan;+;HDI.stan:AM.stan;+;GDP.stan:LE.stan;+;**LE.stan:kcaltav.stan**;+;**AM.stan:kcaltav.stan**	R^2=0.76		Value	Std.Error	t-value	p-value
	P<0.00001	(intercept)	-0.2524435	0.1545591	-1.633.313	0.1114
		HDI.stan	0.3849125	0.1517361	2.536.723	0.0158
		GDP.stan	-0.3832923	0.1599362	-2.396.532	0.0220
		LE.stan	0.4408634	0.2044040	2.156.824	0.0380
		AM.stan	0.3944401	0.1614953	2.442.424	0.0198
		**kcaltav.stan**	0.3220014	0.1522439	2.115.036	0.0416
		HDI.stan:LE.stan	0.7560045	0.2114644	3.575.092	0.0010
		HDI.stan:AM.stan	-0.9080198	0.2329524	-3.897.876	0.0004
		GDP.stan:LE.stan	0.5925259	0.2512393	2.358.412	0.0241
		**LE.stan:kcaltav.stan**	-0.4922407	0.2252609	-2.185.203	0.0357
		**AM.stan:kcaltav.stan**	0.3817109	0.2059104	1.853.772	0.0722
Model:;LN.stan;~;LE.stan;+;AM.stan;+;**ttav.stan**;+;LE.stan:AM.stan;+;**AM.stan:ttav.stan**	R^2=0.60		Value	Std.Error	t-value	p-value
	P<0.00001	(intercept)	-0.0429017	0.1219354	-0.351840	0.7268
		LE.stan	0.3299830	0.2069558	1.594.461	0.1187
		AM.stan	0.5716728	0.1435001	3.983.780	0.0003
		**ttav.stan**	-0.0141092	0.1144473	-0.123281	0.9025
		LE.stan:AM.stan	0.2412797	0.1030433	2.341.537	0.0243
		**AM.stan:ttav.stan**	-0.4681988	0.1387203	-3.375.127	0.0017
Model:;LN.stan;~;GDP.stan;+;AM.stan;+;**Ntav.stan**;+;**GDP.stan:Ntav.stan**	R^2=0.51		Value	Std.Error	t-value	p-value
	P<0.00001	(intercept)	-0.1992387	0.1501329	-1.327.082	0.1918
		GDP.stan	0.0335504	0.1797484	0.186652	0.8529
		AM.stan	0.7558064	0.1857885	4.068.101	0.0002
		**Ntav.stan**	0.0092224	0.1668998	0.055257	0.9562
		**GDP.stan:Ntav.stan**	0.3043890	0.1592234	1.911.710	0.0629
Model:;LN.stan;~;GDP.stan;+;AM.stan;+;**Ptav.stan**;+;**GDP.stan:Ptav.stan**	R^2=0.52		Value	Std.Error	t-value	p-value
	P<0.00001	(intercept)	-0.1944588	0.1434368	-1.355.711	0.1826
		GDP.stan	0.0354593	0.1784232	0.198737	0.8435

		AM.stan	0.7524256	0.1763267	4.267.226	0.0001
		**Ptav.stan**	-0.0146539	0.1551624	-0.094443	0.9252
		**GDP.stan:Ptav.stan**	0.3009661	0.1471401	2.045.439	0.0473
Model:;TN.stan;~;HDI.stan;+;LE.stan;+;AM.stan;+;**Pv.stan**;+;**HDI.stan:Pv.stan**;+;LE.stan:AM.stan;+;**LE.stan:Pv.stan**	R^2=0.62		Value	Std.Error	t-value	p-value
	P<0.00001	(intercept)	-0.1726961	0.1244562	-13.876.053	0.1733
		HDI.stan	0.3434849	0.2307818	14.883.539	0.1449
		LE.stan	0.1840054	0.2322272	0.7923508	0.4331
		AM.stan	0.5696944	0.2032535	28.028.767	0.0079
		**Pv.stan**	0.1728938	0.1130608	15.292.105	0.1345
		**HDI.stan:Pv.stan**	0.3890749	0.1760260	22.103.270	0.0332
		LE.stan:AM.stan	0.3200429	0.1020984	31.346.536	0.0033
		LE.stan:Pv.stan	-0.2840708	0.1370080	-20.733.884	0.0450
Model:;TN.stan;~;HDI.stan;+;GDP.stan;+;LE.stan;+;AM.stan;+;**talc.stan**;+;HDI.stan:LE.stan;+;GDP.stan:LE.stan;+;GDP.stan:AM.stan	R^2=0.69		Value	Std.Error	t-value	p-value
	P<0.00001	(intercept)	-0.0087749	0.1408914	-0.0622811	0.9507
		HDI.stan	0.2626665	0.1939099	13.545.802	0.1838
		GDP.stan	0.3437241	0.1596754	21.526.432	0.0379
		LE.stan	-0.4257843	0.3029715	-14.053.612	0.1683
		AM.stan	0.4956701	0.2245228	22.076.607	0.0335
		**talc.stan**	0.3153031	0.1288161	24.476.984	0.0192
		HDI.stan:LE.stan	0.3566360	0.1269405	28.094.731	0.0079
		GDP.stan:LE.stan	-10.559.996	0.4119497	-25.634.191	0.0146
		GDP.stan:AM.stan	0.6006372	0.2172967	27.641.343	0.0088
Model:;TN.stan;~;HDI.stan;+;GDP.stan;+;AM.stan;+;**Nalc.stan**;+;HDI.stan:GDP.stan;+;**HDI.stan:Nalc.stan**;+;GDP.stan:AM.stan;+;**AM.stan:Nalc.stan**	R^2=0.70		Value	Std.Error	t-value	p-value
	P<0.00001	(intercept)	0.1257152	0.1633835	0.769448	0.4465
		HDI.stan	0.0334642	0.2207947	0.151562	0.8804
		GDP.stan	0.4999174	0.1717198	2.911.240	0.0061
		AM.stan	0.2517090	0.1775083	1.418.013	0.1646
		**Nalc.stan**	-0.3168481	0.2979352	-1.063.480	0.2945
		HDI.stan:GDP.stan	-0.5399972	0.2479735	-2.177.641	0.0359
		**HDI.stan:Nalc.stan**	0.9373007	0.3183356	2.944.379	0.0056
		GDP.stan:AM.stan	0.4500553	0.1849053	2.433.977	0.0199
		**AM.stan:Nalc.stan**	-14.520.710	0.4128528	-3.517.164	0.0012
Model:;TN.stan;~;HDI.stan;+;GDP.stan;+;LE.stan;+;AM.stan;+;**Palc.stan**;+;HDI.stan:LE.stan;+;GDP.stan:LE.stan;+;GDP.stan:AM.stan;+;**LE.stan:Palc.stan**	R^2=0.70		Value	Std.Error	t-value	p-value
	P<0.00001	(intercept)	1.471.615	599.036	2.456.637	0.0190
		HDI.stan	0.25521	0.19707	1.295.027	0.2036
		GDP.stan	0.31293	0.16275	1.922.745	0.0625

		LE.stan	688.917	300.389	2.293.414	0.0278
		AM.stan	0.52284	0.21955	2.381.410	0.0227
		**Palc.stan**	10.004.988	4.055.329	2.467.121	0.0185
		HDI.stan:LE.stan	0.52903	0.13882	3.810.855	0.0005
		GDP.stan:LE.stan	-115.514	0.42678	-2.706.671	0.0103
		GDP.stan:AM.stan	0.54699	0.22291	2.453.851	0.0191
		**LE.stan:Palc.stan**	4.902.897	1.985.895	2.468.861	0.0184
Model:;TN.stan;~;HDI.stan;+;GDP.stan;+;LE.stan;+;AM.stan;+;**protalc.stan**;+;HDI.stan:LE.stan;+;GDP.stan:LE.stan;+;GDP.stan:AM.stan	R^2=0.69		Value	Std.Error	t-value	p-value
	P<0.00001	(intercept)	-0.0443767	0.1402081	-0.3165061	0.7534
		HDI.stan	0.2546112	0.1949351	13.061.328	0.1996
		GDP.stan	0.3545179	0.1598913	22.172.427	0.0328
		LE.stan	-0.3910775	0.3065793	-12.756.162	0.2100
		AM.stan	0.5968826	0.2088030	28.585.915	0.0069
		**protalc.stan**	0.2514047	0.1060450	23.707.364	0.0231
		HDI.stan:LE.stan	0.3922017	0.1261366	31.093.417	0.0036
		GDP.stan:LE.stan	-10.184.625	0.4144967	-24.571.062	0.0188
		GDP.stan:AM.stan	0.5764793	0.2194676	26.267.171	0.0125
;Model:;TN.stan;~;HDI.stan;+;GDP.stan;+;LE.stan;+;AM.stan;+;**tta.stan**;+;HDI.stan:GDP.stan;+;HDI.stan:LE.stan;+;GDP.stan:LE.stan;+;GDP.stan:AM.stan;+;**GDP.stan:tta.stan**	R^2=0.76		Value	Std.Error	t-value	p-value
	P<0.00001	(intercept)	-0.0088341	0.1372317	-0.064374	0.9490
		HDI.stan	0.0170264	0.1941432	0.087700	0.9306
		GDP.stan	0.4203889	0.1655126	2.539.921	0.0157
		LE.stan	-0.5646536	0.2785988	-2.026.763	0.0504
		AM.stan	0.6946883	0.1828759	3.798.687	0.0006
		**tta.stan**	0.3100016	0.1468818	2.110.552	0.0420
		HDI.stan:GDP.stan	-0.7659767	0.2856727	-2.681.308	0.0111
		HDI.stan:LE.stan	0.6978806	0.1510117	4.621.369	0.0001
		GDP.stan:LE.stan	-14.859.851	0.3982501	-3.731.287	0.0007
		GDP.stan:AM.stan	0.5911323	0.2349588	2.515.898	0.0166
		**GDP.stan:tta.stan**	0.7601095	0.2099671	3.620.137	0.0009
Model:;TN.stan;~;HDI.stan;+;GDP.stan;+;LE.stan;+;AM.stan;**+;Nta.stan**;+;HDI.stan:LE.stan;+;**HDI.stan:Nta.stan**;+;GDP.stan:LE.stan;+;**GDP.stan:Nta.stan**;+;**AM.stan:Nta.stan**	R^2=0.75		Value	Std.Error	t-value	p-value
	P<0.00001	(intercept)	-0.3096884	0.1462783	-2.117.118	0.0414
		HDI.stan	0.0883264	0.2071352	0.426419	0.6724
		GDP.stan	0.0494419	0.1497299	0.330207	0.7432
		LE.stan	-0.1339215	0.2309280	-0.579927	0.5657
		AM.stan	0.6504805	0.1721578	3.778.396	0.0006
		**Nta.stan**	0.4595220	0.1870705	2.456.411	0.0191
		HDI.stan:LE.stan	0.5981070	0.1699721	3.518.854	0.0012

		**HDI.stan:Nta.stan**	-0.6431651	0.2658163	-2.419.585	0.0209
		GDP.stan:LE.stan	-10.249.135	0.3349395	-3.059.996	0.0042
		**GDP.stan:Nta.stan**	0.8197962	0.2862955	2.863.462	0.0070
		**AM.stan:Nta.stan**	0.5166563	0.2157864	2.394.295	0.0221
Model:;TN.stan;~;HDI.stan;+;AM.stan;+;**protta.stan**;+;**AM.stan:protta.stan**	R^2=0.68		Value	Std.Error	t-value	p-value
	P<0.00001	(intercept)	-0.4340377	0.1251215	-3.468.928	0.0012
		HDI.stan	0.4682492	0.1905631	2.457.187	0.0183
		AM.stan	0.4516206	0.1531910	2.948.089	0.0053
		**protta.stan**	0.4097078	0.1529684	2.678.383	0.0106
		**AM.stan:protta.stan**	0.6026662	0.1245013	4.840.642	0.0000
Model:;TN.stan;~;HDI.stan;+;GDP.stan;+;LE.stan;+;AM.stan;+;**kcalta.stan**;+;GDP .stan:LE.stan;+;**GDP.stan:kcalta.stan**;+;LE.stan:AM.stan	R^2=0.75		Value	Std.Error	t-value	p-value
	P<0.00001	(intercept)	-0.2988209	0.1274535	-2.344.548	0.0245
		HDI.stan	0.3679150	0.1786864	2.058.998	0.0466
		GDP.stan	-0.0934315	0.1365049	-0.684455	0.4980
		LE.stan	-0.2930709	0.2260104	-1.296.714	0.2028
		AM.stan	0.4664012	0.1642234	2.840.042	0.0073
		**kcalta.stan**	0.5911560	0.1575308	3.752.637	0.0006
		GDP.stan:LE.stan	-14.822.694	0.3920515	-3.780.802	0.0006
		**GDP.stan:kcalta.stan**	0.7937754	0.1933211	4.105.994	0.0002
		LE.stan:AM.stan	0.8507638	0.1855227	4.585.766	0.0001
Model:;TN.stan;~;HDI.stan;+;GDP.stan;+;LE.stan;+;AM.stan;+;**NPta.stan**;+;**HDI. stan:NPta.stan**;+;GDP.stan:LE.stan;+;**GDP.stan:NPta.stan**;+;LE.stan:AM.stan	R^2=0.71		Value	Std.Error	t-value	p-value
	P<0.00001	(intercept)	0.0954610	0.1451056	0.657872	0.5148
		HDI.stan	-0.1533265	0.1406970	-1.089.764	0.2831
		GDP.stan	0.4941934	0.1795733	2.752.042	0.0092
		LE.stan	0.0210316	0.2314517	0.090868	0.9281
		AM.stan	0.4443755	0.1504386	2.953.867	0.0055
		**NPta.stan**	-0.2435601	0.1381048	-1.763.589	0.0863
		**HDI.stan:NPta.stan**	0.3639807	0.1690506	2.153.088	0.0381
		GDP.stan:LE.stan	-0.8718873	0.2873298	-3.034.447	0.0045
		**GDP.stan:NPta.stan**	-0.7114855	0.1738819	-4.091.775	0.0002
		LE.stan:AM.stan	0.5062188	0.1580504	3.202.896	0.0028
Model:;TN.stan;~;HDI.stan;+;GDP.stan;+;AM.stan;+;**NPv.stan**;+;HDI.stan:AM.st an;+;**HDI.stan:NPv.stan**;+;**AM.stan:NPv.stan**	R^2=0.66		Value	Std.Error	t-value	p-value
	P<0.00001	(intercept)	-0.1838391	0.1085795	-1.693.129	0.0986
		HDI.stan	0.3767360	0.1671639	2.253.693	0.0301
		GDP.stan	0.2736534	0.1371604	1.995.133	0.0532
		AM.stan	0.3796241	0.1670409	2.272.642	0.0288

		**NPv.stan**	0.0509714	0.1510369	0.337477	0.7376
		HDI.stan:AM.stan	0.2643842	0.1245912	2.122.014	0.0404
		**HDI.stan:NPv.stan**	-0.3811778	0.1541767	-2.472.343	0.0180
		**AM.stan:NPv.stan**	0.4080029	0.1107395	3.684.349	0.0007
Model:;TN.stan;~;HDI.stan;+;GDP.stan;+;LE.stan;+;AM.stan;+;**NPaa.stan**;+;HDI.stan:GDP.stan;+;HDI.stan:LE.stan;+;**GDP.stan:NPaa.stan**;+;LE.stan:AM.stan;+;**LE.stan:NPaa.stan**	R^2=0.69		Value	Std.Error	t-value	p-value
	P<0.00001	(intercept)	-0.0909849	0.1578284	-0.576479	0.5680
		HDI.stan	0.7028236	0.2259647	3.110.325	0.0037
		GDP.stan	0.1689380	0.1822205	0.927108	0.3602
		LE.stan	-0.0645215	0.2418138	-0.266823	0.7912
		AM.stan	0.3381325	0.2023650	1.670.904	0.1037
		**NPaa.stan**	0.1286905	0.3114276	0.413228	0.6820
		HDI.stan:GDP.stan	0.5515216	0.2558366	2.155.758	0.0381
		HDI.stan:LE.stan	-12.012.790	0.3332820	-3.604.393	0.0010
		**GDP.stan:NPaa.stan**	0.8280650	0.3817247	2.169.273	0.0369
		LE.stan:AM.stan	0.5629368	0.2092666	2.690.046	0.0109
		**LE.stan:NPaa.stan**	-0.6004659	0.1717662	-3.495.832	0.0013
Model:;TN.stan;~;HDI.stan;+;GDP.stan;+;LE.stan;+;AM.stan;+;**talc.stan**;+;HDI.stan:GDP.stan;+;HDI.stan:LE.stan;+;GDP.stan:LE.stan;+;LE.stan:AM.stan	R^2=0.69		Value	Std.Error	t-value	p-value
	P<0.00001	(intercept)	-0.1912138	0.1513420	-12.634.550	0.2145
		HDI.stan	0.1693504	0.1650191	10.262.474	0.3116
		GDP.stan	0.1140716	0.1875956	0.6080717	0.5470
		LE.stan	0.2961572	0.2217718	13.354.141	0.1901
		AM.stan	0.1497799	0.2014325	0.7435736	0.4620
		**talc.stan**	0.4601977	0.1459742	31.525.952	0.0033
		HDI.stan:GDP.stan	0.6334175	0.2620633	24.170.402	0.0208
		HDI.stan:LE.stan	-0.5264514	0.1972909	-26.684.010	0.0114
		GDP.stan:LE.stan	-0.6040813	0.3098898	-19.493.420	0.0591
		LE.stan:AM.stan	0.7338074	0.2344640	31.297.232	0.0035
;Model:;TN.stan;~;GDP.stan;+;LE.stan;+;AM.stan;+;**Nalc.stan**;+;**LE.stan:Nalc.stan**;+;**AM.stan:Nalc.stan;**	R^2=0.65		Value	Std.Error	t-value	p-value
	P<0.00001	(intercept)	0.0306153	0.1080352	0.2833827	0.7784
		GDP.stan	0.3165679	0.1312852	24.112.993	0.0207
		LE.stan	0.2474425	0.1594018	15.523.191	0.1287
		AM.stan	0.1753441	0.1841168	0.9523530	0.3468
		**Nalc.stan**	-0.4741530	0.2740941	-17.298.915	0.0916
		**LE.stan:Nalc.stan**	0.8677387	0.3465344	25.040.474	0.0166
		**AM.stan:Nalc.stan**	-14.516.707	0.4619253	-31.426.523	0.0032
	R^2=0.70		Value	Std.Error	t-value	p-value

Model:;TN.stan;~;HDI.stan;+;GDP.stan;+;LE.stan;+;AM.stan;+;**Palc.stan**;+;HDI.stan:GDP.stan;+;HDI.stan:LE.stan;+;GDP.stan:LE.stan;+;GDP.stan:AM.stan;+;**GDP.stan:Palc.stan**;+;LE.stan:AM.stan	*P*<0.00001	(intercept)	1.307.158	4.415.996	2.960.051	0.0056
		HDI.stan	0.10608	0.174484	0.607980	0.5472
		GDP.stan	1.046.228	3.399.165	3.077.898	0.0041
		LE.stan	0.36758	0.229001	1.605.124	0.1177
		AM.stan	0.18975	0.204184	0.929316	0.3593
		**Palc.stan**	8.959.766	30.034.35	2.983.173	0.0052
		HDI.stan:GDP.stan	104.323	0.375611	2.777.411	0.0089
		HDI.stan:LE.stan	-0.60971	0.218123	-2.795.242	0.0085
		GDP.stan:LE.stan	-0.85741	0.335274	-2.557.342	0.0152
		GDP.stan:AM.stan	-0.63046	0.287530	-2.192.669	0.0353
		**GDP.stan:Palc.stan**	6.960.223	23.321.701	2.984.441	0.0052
		LE.stan:AM.stan	108.931	0.279023	3.904.011	0.0004
Model:;TN.stan;~;HDI.stan;+;GDP.stan;+;LE.stan;+;AM.stan;+;**protalc.stan**;+;HDI.stan:GDP.stan;+;HDI.stan:LE.stan;+;GDP.stan:LE.stan;+;GDP.stan:AM.stan;+;LE.stan:AM.stan	R^2=0.71		Value	Std.Error	t-value	p-value
	P<0.00001	(intercept)	-0.1343485	0.1587349	-0.846370	0.4031
		HDI.stan	0.0341214	0.1715274	0.198927	0.8435
		GDP.stan	0.2158203	0.1856200	1.162.700	0.2528
		LE.stan	0.3895522	0.2251742	1.730.003	0.0924
		AM.stan	0.1805712	0.1985746	0.909337	0.3694
		**protalc.stan**	0.3594175	0.1146341	3.135.345	0.0035
		HDI.stan:GDP.stan	10.455.487	0.3676646	2.843.757	0.0074
		HDI.stan:LE.stan	-0.6152800	0.2127551	-2.891.963	0.0065
		GDP.stan:LE.stan	-0.7152840	0.3095956	-2.310.381	0.0269
		GDP.stan:AM.stan	-0.4766039	0.2702874	-1.763.323	0.0866
		LE.stan:AM.stan	0.9510542	0.2536961	3.748.793	0.0006
Model:;TN.stan;~;HDI.stan;+;GDP.stan;+;LE.stan;+;AM.stan;+;**kcalalc.stan**;+;HDI.stan:AM.stan;+;**HDI.stan:kcalalc.stan**;+;GDP.stan:LE.stan;+;**AM.stan:kcalalc.stan**	R^2=0.70		Value	Std.Error	t-value	p-value
	P<0.00001	(intercept)	0.1468394	0.1673987	0.877183	0.3862
		HDI.stan	0.0076994	0.1483023	0.051917	0.9589
		GDP.stan	0.4837027	0.1788011	2.705.256	0.0104
		LE.stan	-0.0811128	0.2415607	-0.335786	0.7390
		AM.stan	0.2556515	0.1854599	1.378.474	0.1766
		**kcalalc.stan**	0.1014136	0.1163877	0.871343	0.3893
		HDI.stan:AM.stan	0.3473452	0.1244323	2.791.440	0.0083
		**HDI.stan:kcalalc.sta**	0.4140128	0.1122312	3.688.929	0.0007
		GDP.stan:LE.stan	-0.4404255	0.2318479	-1.899.631	0.0655
		**AM.stan:kcalalc.sta**	-0.4819452	0.1350946	-3.567.465	0.0010
Model:;TN.stan;~;HDI.stan;+;GDP.stan;+;LE.stan;+;AM.stan;+;**NPalc.stan**;+;**HDI.stan:NPalc.stan**;+;**GDP.stan:NPalc.stan**;+;**LE.stan:NPalc.stan**	R^2=0.73		Value	Std.Error	t-value	p-value
	P<0.00001	(intercept)	-0.2018697	0.09695023	-2.082.199	0.0443

		HDI.stan	0.4178581	0.18505673	2.258.000	0.0299
		GDP.stan	0.0657857	0.13633678	0.482523	0.6323
		LE.stan	-0.0183204	0.15182012	-0.120672	0.9046
		AM.stan	0.4057654	0.14288970	2.839.711	0.0073
		**NPalc.stan**	-0.3610672	0.15012925	-2.405.043	0.0213
		**HDI.stan:NPalc.sta**	0.5131412	0.23034208	2.227.735	0.0321
		**GDP.stan:NPalc.st**	-0.6110483	0.18530788	-3.297.476	0.0022
		**LE.stan:NPalc.st**	-0.4259436	0.17852447	-2.385.911	0.0223
;Model:;BN.stan;~;HDI.stan;+;GDP.stan;+;LE.stan;+;**Nv.stan**;+;**HDI.stan:Nv.stan** ;+;**LE.stan:Nv.stan;**	R^2=0.63		Value	Std.Error	t-value	p-value
	P<0.00001	(intercept)	-0.0735610	0.1008385	-0.7294930	0.4701
		HDI.stan	0.4968862	0.1797573	27.642.061	0.0087
		GDP.stan	0.3605434	0.1396012	25.826.665	0.0137
		LE.stan	0.1327713	0.1455497	0.9122060	0.3673
		**Nv.stan**	-0.0812380	0.1069831	-0.7593539	0.4522
		**HDI.stan:Nv.stan**	-0.3507796	0.1712995	-20.477.564	0.0474
		**LE.stan:Nv.stan**	0.3361158	0.1379009	24.373.720	0.0195
Model:;BN.stan;~;HDI.stan;+;GDP.stan;+;LE.stan;+;AM.stan;+;**protv.stan**;+;**HDI.stan:protv.stan**;+;GDP.stan:AM.stan;+;LE.stan:AM.stan;+;**LE.stan:protv.stan**	R^2=0.73		Value	Std.Error	t-value	p-value
	P<0.00001	(intercept)	-0.1622056	0.1569595	-1.033.423	0.3083
		HDI.stan	0.8989791	0.2368199	3.796.045	0.0005
		GDP.stan	0.3765105	0.1647624	2.285.172	0.0283
		LE.stan	0.2397159	0.1923025	1.246.556	0.2206
		AM.stan	-0.4074006	0.2101167	-1.938.926	0.0604
		**protv.stan**	-0.3359412	0.1155374	-2.907.640	0.0062
		**HDI.stan:protv.stan**	-0.9503315	0.2791275	-3.404.651	0.0016
		GDP.stan:AM.stan	-0.3894532	0.1832669	-2.125.060	0.0405
		LE.stan:AM.stan	0.3960850	0.1159745	3.415.278	0.0016
		**LE.stan:protv.stan**	0.9852134	0.2870192	3.432.570	0.0015
Model:;BN.stan;~;HDI.stan;+;GDP.stan;+;LE.stan;+;AM.stan;+;**kcalv.stan**;+;**HDI.stan:kcalv.stan**;+;GDP.stan:AM.stan;+;LE.stan:AM.stan;+;**LE.stan:kcalv.stan**	R^2=0.73		Value	Std.Error	t-value	p-value
	P<0.00001	(intercept)	-0.1622056	0.1569595	-1.033.423	0.3083
		HDI.stan	0.8989791	0.2368199	3.796.045	0.0005
		GDP.stan	0.3765105	0.1647624	2.285.172	0.0283
		LE.stan	0.2397159	0.1923025	1.246.556	0.2206
		AM.stan	-0.4074006	0.2101167	-1.938.926	0.0604
		**kcalv.stan**	-0.3359412	0.1155374	-2.907.640	0.0062
		**HDI.stan:kcalv.stan**	-0.9503315	0.2791275	-3.404.651	0.0016
		GDP.stan:AM.stan	-0.3894532	0.1832669	-2.125.060	0.0405
		LE.stan:AM.stan	0.3960850	0.1159745	3.415.278	0.0016

		**LE.stan:kcalv.stan**	0.9852134	0.2870192	3.432.570	0.0015
Model:;BN.stan;~;HDI.stan;+;LE.stan;+;AM.stan;+;**tta.stan**;+;HDI.stan:AM.stan;+;**LE.stan:tta.stan**;+;**AM.stan:tta.stan**	R^2=0.80		Value	Std.Error	t-value	p-value
	P<0.00001	(intercept)	-0.3043266	0.1070733	-2.842.225	0.0072
		HDI.stan	0.4205731	0.1216918	3.456.052	0.0014
		LE.stan	0.3912352	0.1564008	2.501.491	0.0168
		AM.stan	0.1302610	0.1220477	1.067.296	0.2926
		**tta.stan**	0.4644492	0.1156594	4.015.661	0.0003
		HDI.stan:AM.stan	-0.2701606	0.1013042	-2.666.826	0.0112
		**LE.stan:tta.stan**	0.4020586	0.1163507	3.455.576	0.0014
		**AM.stan:tta.stan**	0.3010990	0.1485443	2.026.998	0.0497
;Model:;BN.stan;~;HDI.stan;+;LE.stan;+;AM.stan;+;**Nta.stan**;+;HDI.stan:AM.stan;+;**LE.stan:Nta.stan**	R^2=0.78		Value	Std.Error	t-value	p-value
	P<0.00001	(intercept)	-0.2187708	0.09940731	-2.200.751	0.0337
		HDI.stan	0.4061749	0.12725955	3.191.705	0.0028
		LE.stan	0.3409383	0.16177871	2.107.436	0.0416
		AM.stan	0.0405452	0.13298483	0.304886	0.7621
		**Nta.stan**	0.5218327	0.13879415	3.759.760	0.0006
		HDI.stan:AM.stan	-0.2090310	0.10377531	-2.014.266	0.0509
		**LE.stan:Nta.stan**	0.5053126	0.11902620	4.245.390	0.0001
Model:;BN.stan;~;HDI.stan;+;LE.stan;+;AM.stan;+;**Pta.stan**;+;HDI.stan:AM.stan;+;**LE.stan:Pta.stan**;+;**AM.stan:Pta.stan**;	R^2=0.80		Value	Std.Error	t-value	p-value
	P<0.00001	(intercept)	-0.3129421	0.1089812	-2.871.524	0.0066
		HDI.stan	0.4450452	0.1259553	3.533.359	0.0011
		LE.stan	0.3650116	0.1567541	2.328.561	0.0253
		AM.stan	0.1261869	0.1229951	1.025.951	0.3114
		**Pta.stan**	0.4748275	0.1205129	3.940.054	0.0003
		HDI.stan:AM.stan	-0.2779247	0.1049980	-2.646.951	0.0118
		**LE.stan:Pta.stan**	0.4000343	0.1204574	3.320.961	0.0020
		**AM.stan:Pta.stan**	0.3031983	0.1567218	1.934.627	0.0605
Model:;BN.stan;~;HDI.stan;+;LE.stan;+;**protta.stan**;+;HDI.stan:LE.stan;+;**LE.stan:protta.stan**	R^2=0.77		Value	Std.Error	t-value	p-value
	P<0.00001	(intercept)	-0.2840030	0.09995895	-2.841.197	0.0070
		HDI.stan	0.4385102	0.11595833	3.781.618	0.0005
		LE.stan	0.2965846	0.16262911	1.823.687	0.0757
		**protta.stan**	0.5615235	0.11895653	4.720.410	0.0000
		HDI.stan:LE.stan	-0.3633259	0.11129732	-3.264.462	0.0023
		**LE.stan:protta.stan**	0.7104590	0.15216856	4.668.895	0.0000
	R^2=0.80		Value	Std.Error	t-value	p-value

Model:;BN.stan;~;HDI.stan;+;LE.stan;+;AM.stan;+;**kcalta.stan**;+;HDI.stan:AM.stan;+;LE.stan:AM.stan;+;**AM.stan:kcalta.stan**	*P*<0.00001	(intercept)	-0.3860173	0.12534947	-3.079.529	0.0038
		HDI.stan	0.6463021	0.14983755	4.313.352	0.0001
		LE.stan	0.3601339	0.14911196	2.415.191	0.0206
		AM.stan	-0.0145958	0.14255438	-0.102388	0.9190
		**kcalta.stan**	0.5018529	0.13572074	3.697.687	0.0007
		HDI.stan:AM.stan	-0.5273681	0.13683811	-3.853.956	0.0004
		LE.stan:AM.stan	0.3709064	0.09868249	3.758.584	0.0006
		**AM.stan:kcalta.stan**	0.5615189	0.17244123	3.256.292	0.0024
Model:;BN.stan;~;HDI.stan;+;LE.stan;+;AM.stan;+;**taa.stan**;+;HDI.stan:AM.stan;+;**HDI.stan:taa.stan**;+;LE.stan:AM.stan;+;**LE.stan:taa.stan**;+;**AM.stan:taa.stan**	R^2=0.73		Value	Std.Error	t-value	p-value
	P<0.00001	(intercept)	0.0076647	0.1144025	0.066998	0.9470
		HDI.stan	0.8776686	0.1797733	4.882.086	0.0000
		LE.stan	0.2425487	0.2079539	1.166.358	0.2511
		AM.stan	0.1326678	0.1466121	0.904889	0.3715
		**taa.stan**	-0.0629559	0.1295468	-0.485971	0.6299
		HDI.stan:AM.stan	-0.8245783	0.2088653	-3.947.895	0.0004
		**HDI.stan:taa.stan**	15.217.122	0.4692146	3.243.105	0.0026
		LE.stan:AM.stan	0.8810057	0.1713943	5.140.227	0.0000
		**LE.stan:taa.stan**	-0.9360347	0.3137199	-2.983.664	0.0051
		**AM.stan:taa.stan**	-0.5703245	0.1740793	-3.276.234	0.0023
Model:;BN.stan;~;HDI.stan;+;GDP.stan;+;LE.stan;+;AM.stan;+;**Naa.stan**;+;HDI.stan:AM.stan;+;**HDI.stan:Naa.stan**;+;LE.stan:AM.stan;+;**LE.stan:Naa.stan**;+;**AM.stan:Naa.stan**	R^2=0.76		Value	Std.Error	t-value	p-value
	P<0.00001	(intercept)	0.0962096	0.1233604	0.779907	0.4407
		HDI.stan	0.6918704	0.2036244	3.397.777	0.0017
		GDP.stan	0.3370851	0.1340523	2.514.579	0.0167
		LE.stan	0.0620624	0.2246838	0.276221	0.7840
		AM.stan	0.0123469	0.1548471	0.079736	0.9369
		**Naa.stan**	-0.1498399	0.1039576	-1.441.356	0.1584
		HDI.stan:AM.stan	-0.6258338	0.2094831	-2.987.515	0.0051
		**HDI.stan:Naa.stan**	0.9292053	0.3380719	2.748.543	0.0094
		LE.stan:AM.stan	0.5140024	0.1560492	3.293.849	0.0023
		**LE.stan:Naa.stan**	-0.5241751	0.2682922	-1.953.747	0.0588
		**AM.stan:Naa.stan**	-0.4559249	0.1595360	-2.857.817	0.0071
Model:;BN.stan;~;HDI.stan;+;LE.stan;+;AM.stan;+;**Paa.stan**;+;HDI.stan:AM.stan;+;**HDI.stan:Paa.stan**;+;LE.stan:AM.stan;+;**LE.stan:Paa.stan**;+;**AM.stan:Paa.stan**	R^2=0.73		Value	Std.Error	t-value	p-value
	P<0.00001	(intercept)	0.0035184	0.1141689	0.030817	0.9756
		HDI.stan	0.8717552	0.1796656	4.852.100	0.0000
		LE.stan	0.2593194	0.2054279	1.262.337	0.2149
		AM.stan	0.1268458	0.1467762	0.864212	0.3932
		**Paa.stan**	-0.0738111	0.1282781	-0.575399	0.5686

		HDI.stan:AM.stan	-0.8156843	0.2079277	-3.922.923	0.0004
		**HDI.stan:Paa.stan**	14.846.031	0.4638775	3.200.421	0.0029
		LE.stan:AM.stan	0.8755818	0.1715815	5.103.008	0.0000
		**LE.stan:Paa.stan**	-0.9050388	0.3076493	-2.941.787	0.0057
		**AM.stan:Paa.stan**	-0.5765269	0.1751448	-3.291.715	0.0022
Model:;BN.stan;~;HDI.stan;+;GDP.stan;+;LE.stan;+;AM.stan;+;**protaa.stan**;+;HDI.stan:AM.stan;+;**GDP.stan:protaa.stan**;+;LE.stan:AM.stan;+;**LE.stan:protaa.stan**;+;**AM.stan:protaa.stan**	R^2=0.75		Value	Std.Error	t-value	p-value
	P<0.00001	(intercept)	-0.0682508	0.1153171	-0.591853	0.5578
		HDI.stan	0.6836501	0.1889055	3.619.006	0.0009
		GDP.stan	0.2559767	0.1390329	1.841.123	0.0741
		LE.stan	0.2639779	0.2039078	1.294.594	0.2039
		AM.stan	0.0056960	0.1504235	0.037866	0.9700
		**protaa.stan**	-0.0176855	0.1312001	-0.134798	0.8935
		HDI.stan:AM.stan	-0.4845053	0.1739740	-2.784.929	0.0086
		**GDP.stan:protaa.stan**	0.3904400	0.1815081	2.151.089	0.0385
		LE.stan:AM.stan	0.6398451	0.1780083	3.594.468	0.0010
		**LE.stan:protaa.stan**	-0.5257954	0.2875723	-1.828.394	0.0760
		**AM.stan:protaa.stan**	-0.2914648	0.1501517	-1.941.135	0.0603
Model:;BN.stan;~;HDI.stan;+;GDP.stan;+;LE.stan;+;AM.stan;+;**kcalaa.stan**;+;HDI.stan:AM.stan;+;**HDI.stan:kcalaa.stan**;+;**GDP.stan:kcalaa.stan**;+;LE.stan:AM.stan;+;**LE.stan:kcalaa.stan**;+;**AM.stan:kcalaa.stan**	R^2=0.79		Value	Std.Error	t-value	p-value
	P<0.00001	(intercept)	-0.0012683	0.1248364	-0.010159	0.9920
		HDI.stan	0.6594064	0.1920008	3.434.394	0.0016
		GDP.stan	0.3657299	0.1354625	2.699.861	0.0107
		LE.stan	0.0204075	0.1982415	0.102943	0.9186
		AM.stan	0.0112369	0.1501096	0.074858	0.9408
		**kcalaa.stan**	-0.0051516	0.1741421	-0.029583	0.9766
		HDI.stan:AM.stan	-0.4875513	0.1610280	-3.027.742	0.0047
		**HDI.stan:kcalaa.stan**	0.4685158	0.2110063	2.220.388	0.0332
		**GDP.stan:kcalaa.stan**	0.4886388	0.2300680	2.123.888	0.0410
		LE.stan:AM.stan	0.5828101	0.1703411	3.421.431	0.0016
		**LE.stan:kcalaa.stan**	-10.697.179	0.3947250	-2.710.033	0.0105
		**AM.stan:kcalaa.stan**	-0.4450530	0.2204103	-2.019.202	0.0514
;Model:;BN.stan;~;HDI.stan;+;GDP.stan;+;AM.stan;+;**Nalc.stan**;+;**HDI.stan:Nalc.stan**;+;**AM.stan:Nalc.stan**	R^2=0.71		Value	Std.Error	t-value	p-value
	P<0.00001	(intercept)	-0.0495927	0.09157622	-0.541546	0.5912
		HDI.stan	0.4261709	0.12860588	3.313.775	0.0020
		GDP.stan	0.3903009	0.12048934	3.239.298	0.0025
		AM.stan	0.1002407	0.14413087	0.695484	0.4909
		**Nalc.stan**	-0.7175038	0.26398855	-2.717.935	0.0097
		**HDI.stan:Nalc.stan**	0.4954608	0.23272887	2.128.918	0.0396

		**AM.stan:Nalc.stan**	-0.8094343	0.22473315	-3.601.757	0.0009
Model:;BN.stan;~;HDI.stan;+;GDP.stan;+;LE.stan;+;AM.stan;+;**Palc.stan**;+;**HDI.stan:Palc.stan**;+;GDP.stan:AM.stan;+;LE.stan:AM.stan;+;**LE.stan:Palc.stan**	R^2=0.67		Value	Std.Error	t-value	p-value
	P<0.00001	(intercept)	-270.128	579.722	-0.465961	0.6441
		HDI.stan	3.632.429	1.232.601	2.946.963	0.0056
		GDP.stan	0.59905	0.18009	3.326.463	0.0020
		LE.stan	-2.547.666	976.057	-2.610.160	0.0131
		AM.stan	0.00745	0.19858	0.037539	0.9703
		**Palc.stan**	-1.801.057	3.931.003	-0.458167	0.6496
		**HDI.stan:Palc.stan**	24.364.418	8.323.134	2.927.313	0.0059
		GDP.stan:AM.stan	-0.52380	0.20745	-2.524.863	0.0161
		LE.stan:AM.stan	0.31980	0.11828	2.703.801	0.0104
		**LE.stan:Palc.stan**	-17.364.391	6.562.034	-2.646.191	0.0120
;Model:;BN.stan;~;HDI.stan;+;GDP.stan;+;LE.stan;+;AM.stan;+;**protalc.stan**;+;HDI.stan:LE.stan;+;HDI.stan:AM.stan;+;**HDI.stan:protalc.stan**;+;**LE.stan:protalc.stan;**	R^2=0.70		Value	Std.Error	t-value	p-value
	P<0.00001	(intercept)	-0.1762243	0.1280699	-1.376.000	0.1773
		HDI.stan	0.7719180	0.1858703	4.152.993	0.0002
		GDP.stan	0.3245758	0.1404674	2.310.685	0.0267
		LE.stan	-0.0629292	0.1987636	-0.316603	0.7534
		AM.stan	0.0538919	0.1703295	0.316398	0.7535
		**protalc.stan**	-0.0587920	0.1099405	-0.534762	0.5961
		HDI.stan:LE.stan	0.3941368	0.1890745	2.084.558	0.0443
		HDI.stan:AM.stan	-0.3946063	0.2031037	-1.942.880	0.0599
		**HDI.stan:protalc.stan**	0.7220894	0.2016996	3.580.025	0.0010
		**LE.stan:protalc.stan**	-0.5511425	0.2084666	-2.643.792	0.0121
Model:;BN.stan;~;HDI.stan;+;GDP.stan;+;LE.stan;+;AM.stan;+;**kcalb.stan**;+;HDI.stan:LE.stan;+;HDI.stan:AM.stan;+;**HDI.stan:kcalb.stan**;+;**LE.stan:kcalb.stan**	R^2=0.72		Value	Std.Error	t-value	p-value
	P<0.00001	(intercept)	-0.1228039	0.1119681	-1.096.776	0.2800
		HDI.stan	0.6343603	0.1522403	4.166.837	0.0002
		GDP.stan	0.3427611	0.1350950	2.537.185	0.0157
		LE.stan	-0.0054934	0.1821658	-0.030156	0.9761
		AM.stan	0.1514818	0.1691287	0.895660	0.3764
		**kcalb.stan**	-0.1026727	0.1121536	-0.915465	0.3660
		HDI.stan:LE.stan	0.3979314	0.1853438	2.146.991	0.0386
		HDI.stan:AM.stan	-0.3862007	0.2044587	-1.888.894	0.0670
		**HDI.stan:kcalb.stan**	0.4981101	0.1338056	3.722.641	0.0007
		**LE.stan:kcalb.stan**	-0.3876741	0.1715770	-2.259.476	0.0300
Model:;BN.stan;~;HDI.stan;+;GDP.stan;+;LE.stan;+;AM.stan;+;**NPalc.stan**;+;HDI.stan:LE.stan;+;HDI.stan:AM.stan;+;**HDI.stan:NPalc.stan**;+;**AM.stan:NPalc.stan**	R^2=0.82		Value	Std.Error	t-value	p-value
	P<0.00001	(intercept)	-0.1008572	0.09355537	-1.078.048	0.2882

		HDI.stan	0.7216666	0.13136904	5.493.430	0.0000
		GDP.stan	0.2433743	0.10715722	2.271.189	0.0292
		LE.stan	-0.0119919	0.15125614	-0.079282	0.9372
		AM.stan	-0.0403415	0.12417735	-0.324870	0.7472
		**NPalc.stan**	-0.5130705	0.10617103	-4.832.491	0.0000
		HDI.stan:LE.stan	0.2866424	0.14610646	1.961.873	0.0575
		HDI.stan:AM.stan	-0.3872736	0.15624738	-2.478.593	0.0180
		**HDI.stan:NPalc.stan**	0.4649539	0.14419299	3.224.525	0.0027
		**AM.stan:NPalc.stan**	-0.7368207	0.12447758	-5.919.305	0.0000
Model:;BN.stan;~;HDI.stan;+;GDP.stan;+;LE.stan;+;AM.stan;+;**NPv.stan**;+;**HDI.stan:NPv.stan**;+;**LE.stan:NPv.stan**;+;**AM.stan:NPv.stan**	R^2=0.76		Value	Std.Error	t-value	p-value
	P<0.00001	(intercept)	-0.1282441	0.0892205	-1.437.383	0.1590
		HDI.stan	0.9177592	0.1883293	4.873.162	0.0000
		GDP.stan	0.2923883	0.1170595	2.497.775	0.0171
		LE.stan	-0.1851014	0.1375911	-1.345.301	0.1867
		AM.stan	0.0173370	0.1400570	0.123785	0.9022
		**NPv.stan**	0.1572214	0.1286150	1.222.419	0.2293
		**HDI.stan:NPv.stan**	-0.4597403	0.1363323	-3.372.204	0.0018
		**LE.stan:NPv.stan**	0.3271650	0.1517026	2.156.622	0.0376
		**AM.stan:NPv.stan**	0.2892874	0.1172145	2.468.017	0.0183
Model:;BN.stan;~;HDI.stan;+;GDP.stan;+;LE.stan;+;AM.stan;+;**NPta.stan**;+;HDI.stan:LE.stan;+;HDI.stan:AM.stan;+;**HDI.stan:NPta.stan**;+;GDP.stan:LE.stan;+;**LE.stan:NPta.stan**;+;**AM.stan:NPta.stan**	R^2=0.75		Value	Std.Error	t-value	p-value
	P<0.00001	(intercept)	0.2718829	0.1652641	1.645.142	0.1092
		HDI.stan	-0.1113980	0.2003096	-0.556129	0.5818
		GDP.stan	0.5627475	0.2065577	2.724.408	0.0101
		LE.stan	0.0363633	0.2237560	0.162513	0.8719
		AM.stan	0.3412840	0.1733342	1.968.936	0.0572
		**NPta.stan**	-0.4621179	0.1308299	-3.532.203	0.0012
		HDI.stan:LE.stan	0.7596641	0.2206585	3.442.713	0.0015
		HDI.stan:AM.stan	-0.5727702	0.2008700	-2.851.448	0.0073
		**HDI.stan:NPta.stan**	0.6750160	0.2935645	2.299.379	0.0278
		GDP.stan:LE.stan	-0.6511629	0.3393667	-1.918.759	0.0634
		**LE.stan:NPta.stan**	-0.5100525	0.2840505	-1.795.640	0.0814
		**AM.stan:NPta.stan**	-0.5455894	0.1494374	-3.650.957	0.0009
Model:;CeN.stan;~;GDP.stan;+;LE.stan;+;**tv.stan**;+;GDP.stan:LE.stan;+;**GDP.stan:tv.stan**;+;**LE.stan:tv.stan**	R^2=0.84		Value	Std.Error	t-value	p-value
	P<0.00001	(intercept)	-0.2677540	0.09628879	-2.780.739	0.0083
		GDP.stan	-0.3822873	0.09818262	-3.893.635	0.0004
		LE.stan	-0.2278412	0.15169757	-1.501.944	0.1412
		**tv.stan**	-0.0728045	0.07217614	-1.008.706	0.3193

		GDP.stan:LE.stan	0.4803722	0.12415591	3.869.104	0.0004
		**GDP.stan:tv.stan**	0.2649901	0.09137557	2.900.011	0.0061
		**LE.stan:tv.stan**	-0.2364739	0.05822991	-4.061.039	0.0002
Model:;CeN.stan;~;HDI.stan;+;GDP.stan;+;LE.stan;+;**Nv.stan**;+;HDI.stan:LE.stan	R^2=0.84		Value	Std.Error	t-value	p-value
	P<0.00001	(intercept)	-0.1649462	0.07038531	-2.343.476	0.0242
		HDI.stan	-0.2208701	0.08417472	-2.623.948	0.0122
		GDP.stan	-0.1994278	0.08244922	-2.418.796	0.0202
		LE.stan	-0.1618549	0.12687721	-1.275.682	0.2094
		**Nv.stan**	-0.2857010	0.06535319	-4.371.646	0.0001
		HDI.stan:LE.stan	0.2872213	0.05805973	4.946.996	0.0000
Model:;CeN.stan;~;HDI.stan;+;LE.stan;+;AM.stan;+;**Pv.stan**;+;HDI.stan:LE.stan;+;**HDI.stan:Pv.stan**;+;**LE.stan:Pv.stan**	R^2=0.88		Value	Std.Error	t-value	p-value
	P<0.00001	(intercept)	-0.0729666	0.06836792	-1.067.264	0.2926
		HDI.stan	-0.5529470	0.09978360	-5.541.462	0.0000
		LE.stan	-0.4147962	0.12707805	-3.264.106	0.0023
		AM.stan	0.2739198	0.10714887	2.556.442	0.0147
		**Pv.stan**	-0.2083947	0.05992212	-3.477.759	0.0013
		HDI.stan:LE.stan	0.2796405	0.05931538	4.714.468	0.0000
		**HDI.stan:Pv.stan**	0.6313027	0.14222663	4.438.709	0.0001
		**LE.stan:Pv.stan**	-0.2561166	0.09485032	-2.700.218	0.0103
Model:;CeN.stan;~;HDI.stan;+;LE.stan;+;AM.stan;+;**protv.stan**;+;HDI.stan:LE.stan;+;**HDI.stan:protv.stan**;+;**AM.stan:protv.stan**	R^2=0.83		Value	Std.Error	t-value	p-value
	P<0.00001	(intercept)	-0.0897567	0.07847193	-1.143.807	0.2599
		HDI.stan	-0.5860098	0.16842015	-3.479.452	0.0013
		LE.stan	-0.2455329	0.13312920	-1.844.320	0.0729
		AM.stan	0.0823159	0.12767746	0.644718	0.5230
		**protv.stan**	-0.2887739	0.08980967	-3.215.398	0.0027
		HDI.stan:LE.stan	0.2471749	0.06299611	3.923.653	0.0004
		**HDI.stan:protv.stan**	0.3891993	0.16955652	2.295.396	0.0273
		**AM.stan:protv.stan**	-0.2710904	0.12519245	-2.165.390	0.0367
Model:;CeN.stan;~;HDI.stan;+;LE.stan;+;AM.stan;+;**kcalv.stan**;+;HDI.stan:LE.stan;+;**HDI.stan:kcalv.stan**;+;**AM.stan:kcalv.stan**	R^2=0.83		Value	Std.Error	t-value	p-value
	P<0.00001	(intercept)	-0.0897567	0.07847193	-1.143.807	0.2599
		HDI.stan	-0.5860098	0.16842015	-3.479.452	0.0013
		LE.stan	-0.2455329	0.13312920	-1.844.320	0.0729
		AM.stan	0.0823159	0.12767746	0.644718	0.5230
		**kcalv.stan**	-0.2887739	0.08980967	-3.215.398	0.0027
		HDI.stan:LE.stan	0.2471749	0.06299611	3.923.653	0.0004
		**HDI.stan:kcalv.stan**	0.3891993	0.16955652	2.295.396	0.0273

		**AM.stan:kcalv.stan**	-0.2710904	0.12519245	-2.165.390	0.0367
;Model:;CeN.stan;~;HDI.stan;+;LE.stan;+;AM.stan;+;**protv.stan**;+;HDI.stan:LE.stan;+;HDI.stan:**protv.stan**;+;**AM.stan:protv.stan**;	R^2=0.83		Value	Std.Error	t-value	p-value
	P<0.00001	(intercept)	-0.0897567	0.07847193	-1.143.807	0.2599
		HDI.stan	-0.5860098	0.16842015	-3.479.452	0.0013
		LE.stan	-0.2455329	0.13312920	-1.844.320	0.0729
		AM.stan	0.0823159	0.12767746	0.644718	0.5230
		**protv.stan**	-0.2887739	0.08980967	-3.215.398	0.0027
		HDI.stan:LE.stan	0.2471749	0.06299611	3.923.653	0.0004
		**HDI.stan:protv.stan**	0.3891993	0.16955652	2.295.396	0.0273
		**AM.stan:protv.stan**	-0.2710904	0.12519245	-2.165.390	0.0367
;Model:;CeN.stan;~;HDI.stan;+;LE.stan;+;AM.stan;+;**kcalv.stan**;+;HDI.stan:LE.stan;+;**HDI.stan:kcalv.stan**;+;**AM.stan:kcalv.stan**;	R^2=0.83		Value	Std.Error	t-value	p-value
	P<0.00001	(intercept)	-0.0897567	0.07847193	-1.143.807	0.2599
		HDI.stan	-0.5860098	0.16842015	-3.479.452	0.0013
		LE.stan	-0.2455329	0.13312920	-1.844.320	0.0729
		AM.stan	0.0823159	0.12767746	0.644718	0.5230
		**kcalv.stan**	-0.2887739	0.08980967	-3.215.398	0.0027
		HDI.stan:LE.stan	0.2471749	0.06299611	3.923.653	0.0004
		**HDI.stan:kcalv.stan**	0.3891993	0.16955652	2.295.396	0.0273
		**AM.stan:kcalv.stan**	-0.2710904	0.12519245	-2.165.390	0.0367
Model:;CeN.stan;~;HDI.stan;+;GDP.stan;+;LE.stan;+;**tta.stan**;+;HDI.stan:LE.stan;+;GDP.stan:LE.stan;+;LE.stan:tta.stan	R^2=0.81		Value	Std.Error	t-value	p-value
	P<0.00001	(intercept)	-0.2583184	0.10823722	-23.865.949	0.0221
		HDI.stan	-0.1555249	0.10834572	-14.354.505	0.1593
		GDP.stan	-0.4067661	0.14545586	-27.964.913	0.0081
		LE.stan	-0.1873336	0.16934688	-11.062.123	0.2756
		**tta.stan**	0.0774026	0.12340099	0.6272442	0.5343
		HDI.stan:LE.stan	0.2557330	0.09225909	27.719.003	0.0086
		GDP.stan:LE.stan	0.6065596	0.22909850	26.475.931	0.0117
		LE.stan:tta.stan	-0.3681897	0.16491424	-22.326.130	0.0315
;Model:;CeN.stan;~;HDI.stan;+;GDP.stan;+;LE.stan;+;**Nta.stan**;+;HDI.stan:LE.stan;+;GDP.stan:LE.stan;+;**LE.stan:Nta.stan**	R^2=0.82		Value	Std.Error	t-value	p-value
	P<0.00001	(intercept)	-0.2548106	0.10678296	-2.386.247	0.0221
		HDI.stan	-0.2087884	0.10991432	-1.899.556	0.0651
		GDP.stan	-0.4883085	0.15599580	-3.130.267	0.0034
		LE.stan	-0.1838755	0.16399482	-1.121.228	0.2692
		**Nta.stan**	0.1739020	0.14167236	1.227.494	0.2272
		HDI.stan:LE.stan	0.3115514	0.09680466	3.218.351	0.0026
		GDP.stan:LE.stan	0.7147771	0.23801191	3.003.115	0.0047

		**LE.stan:Nta.stan**	-0.4883443	0.17952549	-2.720.195	0.0098
Model:;CeN.stan;~;HDI.stan;+;GDP.stan;+;LE.stan;+;**Pta.stan**;+;HDI.stan:LE.stan;+;GDP.stan:LE.stan;+;**LE.stan:Pta.stan**	R^2=0.82		Value	Std.Error	t-value	p-value
	P<0.00001	(intercept)	-0.2526376	0.10749108	-23.503.118	0.0241
		HDI.stan	-0.1722331	0.10855077	-15.866.590	0.1209
		GDP.stan	-0.4277426	0.14862108	-28.780.751	0.0065
		LE.stan	-0.1909435	0.16727011	-11.415.280	0.2608
		**Pta.stan**	0.1009309	0.12989488	0.7770197	0.4420
		HDI.stan:LE.stan	0.2747103	0.09339751	29.413.018	0.0055
		GDP.stan:LE.stan	0.6423247	0.23184885	27.704.458	0.0086
		**LE.stan:Pta.stan**	-0.4150043	0.17044378	-24.348.459	0.0197
Model:;CeN.stan;~;HDI.stan;+;GDP.stan;+;LE.stan;+;**protta.stan**;+;HDI.stan:LE.stan;+;GDP.stan:LE.stan;+;**LE.stan:protta.stan**	R^2=0.81		Value	Std.Error	t-value	p-value
	P<0.00001	(intercept)	-0.2451982	0.1082395	-22.653.310	0.0293
		HDI.stan	-0.1843461	0.1099029	-16.773.543	0.1017
		GDP.stan	-0.4700772	0.1695028	-27.732.714	0.0086
		LE.stan	-0.1553085	0.1655960	-0.9378760	0.3542
		**protta.stan**	0.1129391	0.1568011	0.7202699	0.4758
		HDI.stan:LE.stan	0.3267360	0.1043872	31.300.396	0.0034
		GDP.stan:LE.stan	0.7012169	0.2583645	27.140.605	0.0099
		**LE.stan:protta.stan**	-0.4804978	0.1988777	-24.160.466	0.0206
Model:;CeN.stan;~;HDI.stan;+;GDP.stan;+;LE.stan;+;**kcalta.stan;**+;HDI.stan:LE.stan;+;GDP.stan:LE.stan;+;**LE.stan:kcalta.stan**	R^2=0.81		Value	Std.Error	t-value	p-value
	P<0.00001	(intercept)	-0.2834433	0.10697794	-2.649.549	0.0117
		HDI.stan	-0.1828135	0.10880375	-1.680.214	0.1011
		GDP.stan	-0.5128353	0.15663966	-3.273.981	0.0023
		LE.stan	-0.1771935	0.16727035	-1.059.324	0.2961
		**kcalta.stan**	0.2273421	0.14144989	1.607.227	0.1163
		HDI.stan:LE.stan	0.2823748	0.09555051	2.955.242	0.0053
		GDP.stan:LE.stan	0.7105074	0.24379016	2.914.422	0.0059
		**LE.stan:kcalta.stan**	-0.4391598	0.18686735	-2.350.115	0.0241
Model:;CeN.stan;~;HDI.stan;+;GDP.stan;+;LE.stan;+;**Naa.stan;**+;**HDI.stan:Naa.stan;**+;GDP.stan:LE.stan	R^2=0.79		Value	Std.Error	t-value	p-value
	P<0.00001	(intercept)	-0.2686427	0.11108961	-2.418.253	0.0204
		HDI.stan	0.0306642	0.09965287	0.307710	0.7599
		GDP.stan	-0.4413469	0.12293930	-3.589.958	0.0009
		LE.stan	-0.1475315	0.17796939	-0.828971	0.4122
		**Naa.stan**	0.0433830	0.08295721	0.522956	0.6040
		**HDI.stan:Naa.stan**	-0.1689794	0.08095173	-2.087.409	0.0434
		GDP.stan:LE.stan	0.5026791	0.14983547	3.354.874	0.0018
Model:;CeN.stan;~;HDI.stan;+;GDP.stan;+;LE.stan;+;**kcalaa.stan;**+;HDI.stan:LE.stan;+;**GDP.stan:kcalaa.stan**	R^2=0.79		Value	Std.Error	t-value	p-value
	P<0.00001	(intercept)	-0.2413846	0.09472846	-2.548.174	0.0149

		HDI.stan	-0.1645625	0.09850224	-1.670.648	0.1028
		GDP.stan	-0.1441139	0.10407616	-1.384.697	0.1740
		LE.stan	-0.2526487	0.14533548	-1.738.383	0.0900
		**kcalaa.stan**	-0.2010074	0.10668728	-1.884.080	0.0670
		HDI.stan:LE.stan	0.2641533	0.06748142	3.914.460	0.0004
		**GDP.stan:kcalaa.stan**	0.2725129	0.14207156	1.918.138	0.0624
Model:;CeN.stan;~;HDI.stan;+;GDP.stan;+;LE.stan;+;AM.stan;+;**talc.stan;**+;HDI.stan:LE.stan;+;HDI.stan:AM.stan;+;GDP.stan:LE.stan;+;**LE.stan:talc.stan**	R^2=0.88		Value	Std.Error	t-value	p-value
	P<0.00001	(intercept)	-0.2768372	0.09018629	-3.069.615	0.0041
		HDI.stan	-0.0065895	0.09606493	-0.068594	0.9457
		GDP.stan	-0.5258015	0.11220040	-4.686.271	0.0000
		LE.stan	-0.0791034	0.13964184	-0.566474	0.5746
		AM.stan	-0.2581216	0.12230463	-2.110.481	0.0418
		**talc.stan**	0.3632742	0.09039974	4.018.532	0.0003
		HDI.stan:LE.stan	-0.5085736	0.16287418	-3.122.494	0.0035
		HDI.stan:AM.stan	0.3611781	0.13074702	2.762.420	0.0090
		GDP.stan:LE.stan	0.7857255	0.17326274	4.534.879	0.0001
		**LE.stan:talc.sta**	-0.3401790	0.08816614	-3.858.386	0.0005
Model:;CeN.stan;~;GDP.stan;+;LE.stan;+;**Nalc.stan**;+;GDP.stan:LE.stan	R^2=0.81		Value	Std.Error	t-value	p-value
	P<0.00001	(intercept)	-0.2295616	0.09973350	-2.301.750	0.0265
		GDP.stan	-0.4101903	0.10254986	-3.999.911	0.0003
		LE.stan	-0.1349749	0.15700178	-0.859703	0.3950
		**Nalc.stan**	0.2793576	0.08159921	3.423.533	0.0014
		GDP.stan:LE.stan	0.4119559	0.13323971	3.091.840	0.0036
Model:;CeN.stan;~;GDP.stan;+;LE.stan;+;**Palc.stan**;+;GDP.stan:LE.stan;+;**GDP.stan:Palc.stan**	R^2=0.81		Value	Std.Error	t-value	p-value
	P<0.00001	(intercept)	527.114	2.983.606	1.766.701	0.0849
		GDP.stan	385.410	2.289.574	1.683.328	0.1001
		LE.stan	-0.12838	0.160295	-0.800915	0.4279
		**Palc.stan**	3.810.755	20.399.693	1.868.045	0.0691
		GDP.stan:LE.stan	0.60822	0.132360	4.595.237	0.0000
		**GDP.stan:Palc.st**	2.975.465	15.844.417	1.877.927	0.0677
Model:;CeN.stan;~;HDI.stan;+;GDP.stan;+;LE.stan;+;AM.stan;+;**protalc.stan**;+;HDI.stan:AM.stan;+;GDP.stan:LE.stan;+;LE.stan:AM.stan;+;**LE.stan:protalc.stan**	R^2=0.86		Value	Std.Error	t-value	p-value
	P<0.00001	(intercept)	-0.3042693	0.09494519	-3.204.684	0.0028
		HDI.stan	-0.1965346	0.11309235	-1.737.824	0.0908
		GDP.stan	-0.4756197	0.11835056	-4.018.736	0.0003
		LE.stan	-0.0772585	0.15194837	-0.508452	0.6142
		AM.stan	-0.0485436	0.11574271	-0.419410	0.6774

		**protalc.stan**	0.2756193	0.07558781	3.646.347	0.0008
		HDI.stan:AM.stan	0.1993168	0.09802802	2.033.263	0.0494
		GDP.stan:LE.stan	0.7397579	0.20362742	3.632.899	0.0009
		LE.stan:AM.stan	-0.2553502	0.13657172	-1.869.715	0.0697
		**LE.stan:protalc.st**	-0.2932299	0.09886413	-2.965.988	0.0053
Model:;CeN.stan;~;HDI.stan;+;GDP.stan;+;LE.stan;+;AM.stan;+;**kcalalc.stan**;+;HDI.stan:AM.stan;+;GDP.stan:LE.stan;+;**LE.stan:kcalalc.stan**;+;HDI.stan:LE.stan	R^2=0.86		Value	Std.Error	t-value	p-value
	P<0.00001	(intercept)	-0.4116983	0.10016070	-4.110.378	0.0002
		HDI.stan	-0.1164471	0.09957286	-1.169.466	0.2499
		GDP.stan	-0.4959615	0.12078268	-4.106.230	0.0002
		LE.stan	0.1382344	0.16113756	0.857866	0.3966
		AM.stan	-0.1590790	0.12028594	-1.322.507	0.1943
		**kcalalc.stan**	0.2964062	0.08341101	3.553.562	0.0011
		HDI.stan:AM.stan	0.4534641	0.15264240	2.970.761	0.0053
		GDP.stan:LE.stan	0.7145901	0.18149311	3.937.285	0.0004
		**LE.stan:kcalalc.st**	-0.3488492	0.10928142	-3.192.210	0.0029
		HDI.stan:LE.stan	-0.3708361	0.16364068	-2.266.161	0.0296
Model:;CeN.stan;~;GDP.stan;+;LE.stan;+;**NPta.stan**;+;GDP.stan:LE.stan;+;**LE.stan:NPta.stan**	R^2=0.81		Value	Std.Error	t-value	p-value
	P<0.00001	(intercept)	-0.2801028	0.09989211	-2.804.054	0.0077
		GDP.stan	-0.3836291	0.10486000	-3.658.489	0.0007
		LE.stan	-0.2001562	0.15968020	-1.253.482	0.2173
		**NPta.stan**	0.1076256	0.08290889	1.298.119	0.2017
		GDP.stan:LE.stan	0.5414428	0.13129292	4.123.930	0.0002
		**LE.stan:NPta.stan**	0.2585153	0.08026779	3.220.660	0.0025
Model:;CeN.stan;~;HDI.stan;+;GDP.stan;+;LE.stan;+;AM.stan;+;**NPaa.stan**;+;**HDI.stan:NPaa.stan**;+;GDP.stan:LE.stan;+;GDP.stan:AM.stan;+;LE.stan:AM.stan;+;**LE.stan:NPaa.stan**	R^2=0.85		Value	Std.Error	t-value	p-value
	P<0.00001	(intercept)	-0.2966427	0.1133574	-26.168.799	0.0130
		HDI.stan	0.1889270	0.1183530	15.963.017	0.1194
		GDP.stan	-0.3867764	0.1395119	-27.723.539	0.0089
		LE.stan	-0.3816806	0.1856009	-20.564.590	0.0473
		AM.stan	0.0486607	0.1384282	0.3515232	0.7273
		**NPaa.stan**	0.2423020	0.1856562	13.051.114	0.2004
		**HDI.stan:NPaa.stan**	0.6823778	0.3409894	20.011.699	0.0532
		GDP.stan:LE.stan	0.4100587	0.2216752	18.498.179	0.0728
		GDP.stan:AM.stan	0.3159350	0.1462918	21.596.212	0.0377
		LE.stan:AM.stan	-0.3211010	0.1600802	-20.058.762	0.0526
		**LE.stan:NPaa.stan**	-0.6241153	0.2793425	-22.342.292	0.0320
	R^2=0.80		Value	Std.Error	t-value	p-value

Model:;CeN.stan;~;GDP.stan;+;LE.stan;+;**NPalc.stan**;+;GDP.stan:LE.stan;+;**LE.stan:NPalc.stan**	*P*<0.00001	(intercept)	-0.2577906	0.10327160	-2.496.240	0.0168
		GDP.stan	-0.3725156	0.10960954	-3.398.569	0.0015
		LE.stan	-0.1756737	0.16310280	-1.077.073	0.2879
		**NPalc.stan**	0.0398745	0.09688990	0.411544	0.6829
		GDP.stan:LE.stan	0.3893593	0.14283510	2.725.935	0.0095
		**LE.stan:NPalc.st**	-0.0945604	0.04372268	-2.162.731	0.0366
Model:;PN.stan;~;HDI.stan;+;GDP.stan;+;LE.stan;+;**tv.stan**;+;HDI.stan:GDP.stan;+;**HDI.stan:tv.stan**;+;**GDP.stan:tv.stan**;+;**LE.stan:tv.stan**	R^2=0.54		Value	Std.Error	t-value	p-value
	P<0.00001	(intercept)	-0.4734312	0.2163308	-2.188.460	0.0350
		HDI.stan	0.8398197	0.2140307	3.923.829	0.0004
		GDP.stan	-0.1417407	0.2247486	-0.630663	0.5321
		LE.stan	0.4432136	0.2681186	1.653.051	0.1068
		**tv.stan**	-0.0204684	0.1316647	-0.155459	0.8773
		HDI.stan:GDP.stan	0.6182788	0.2805754	2.203.610	0.0339
		**HDI.stan:tv.stan**	-10.896.310	0.3288726	-3.313.231	0.0021
		**GDP.stan:tv.stan**	0.3926699	0.2146308	1.829.513	0.0754
		**LE.stan:tv.stan**	0.5218831	0.1927230	2.707.944	0.0102
Model:;PN.stan;~;HDI.stan;+;GDP.stan;+;LE.stan;+;AM.stan;+;**Nv.stan**;+;HDI.stan:GDP.stan;+;HDI.stan:LE.stan;+;**HDI.stan:Nv.stan**;+;GDP.stan:AM.stan;+;**GDP.stan:Nv.stan**;+;LE.stan:AM.stan;+;**LE.stan:Nv.stan**;+;**AM.stan:Nv.stan**	R^2=0.72		Value	Std.Error	t-value	p-value
	P<0.00001	(intercept)	-0.7053430	0.2165746	-3.256.814	0.0027
		HDI.stan	15.786.660	0.3577281	4.413.033	0.0001
		GDP.stan	-0.2548895	0.2201000	-1.158.062	0.2554
		LE.stan	0.5852873	0.2500059	2.341.094	0.0256
		AM.stan	-0.5388160	0.2109773	-2.553.905	0.0156
		**Nv.stan**	-0.1943421	0.1528192	-1.271.713	0.2126
		HDI.stan:GDP.stan	10.810.007	0.3802678	2.842.735	0.0077
		HDI.stan:LE.stan	0.7331141	0.2962431	2.474.705	0.0188
		**HDI.stan:Nv.stan**	-21.312.212	0.4251118	-5.013.319	0.0000
		GDP.stan:AM.stan	-0.4815197	0.2739161	-1.757.910	0.0883
		**GDP.stan:Nv.stan**	0.5014739	0.2603312	1.926.292	0.0630
		LE.stan:AM.stan	-0.5201627	0.3099848	-1.678.026	0.1031
		**LE.stan:Nv.stan**	0.9903492	0.2656937	3.727.409	0.0007
		**AM.stan:Nv.stan**	0.4671918	0.2025063	2.307.048	0.0277
Model:;PN.stan;~;HDI.stan;+;LE.stan;+;AM.stan;+;**Pv.stan**;+;HDI.stan:LE.stan;+;HDI.stan:AM.stan	R^2=0.51		Value	Std.Error	t-value	p-value
	P<0.00001	(intercept)	-0.0238907	0.1307822	-0.182675	0.8560
		HDI.stan	0.4838121	0.1690729	2.861.560	0.0067
		LE.stan	0.2015813	0.2142871	0.940707	0.3526
		AM.stan	0.1025864	0.1754130	0.584828	0.5620
		**Pv.stan**	-0.3848666	0.1178890	-3.264.653	0.0023
		HDI.stan:LE.stan	0.5757620	0.1969983	2.922.675	0.0057
		HDI.stan:AM.stan	-0.5583216	0.2401136	-2.325.240	0.0254

Model:;PN.stan;~;HDI.stan;+;LE.stan;+;AM.stan;+;**protv.stan;**+;HDI.stan:AM.stan;+;**HDI.stan:protv.stan**;+;LE.stan:AM.stan	R^2=0.59		Value	Std.Error	t-value	p-value
	P<0.00001	(intercept)	-0.1838452	0.1274333	-1.442.678	0.1573
		HDI.stan	10.794.410	0.2062739	5.233.048	0.0000
		LE.stan	0.2494548	0.2083046	1.197.548	0.2385
		AM.stan	-0.4111814	0.1968869	-2.088.414	0.0435
		**protv.stan**	-0.4703003	0.1275525	-3.687.111	0.0007
		HDI.stan:AM.stan	-0.4421157	0.1648613	-2.681.743	0.0108
		**HDI.stan:protv.stan**	-0.5673808	0.1700111	-3.337.315	0.0019
		LE.stan:AM.stan	0.4381476	0.1427727	3.068.846	0.0040
;Model:;PN.stan;~;HDI.stan;+;LE.stan;+;AM.stan;+;**kcalv.stan;**+;HDI.stan:AM.stan;+;**HDI.stan:kcalv.stan**;+;LE.stan:AM.stan	R^2=0.59		Value	Std.Error	t-value	p-value
	P<0.00001	(intercept)	-0.1838452	0.1274333	-1.442.678	0.1573
		HDI.stan	10.794.410	0.2062739	5.233.048	0.0000
		LE.stan	0.2494548	0.2083046	1.197.548	0.2385
		AM.stan	-0.4111814	0.1968869	-2.088.414	0.0435
		**kcalv.stan**	-0.4703003	0.1275525	-3.687.111	0.0007
		HDI.stan:AM.stan	-0.4421157	0.1648613	-2.681.743	0.0108
		**HDI.stan:kcalv.stan**	-0.5673808	0.1700111	-3.337.315	0.0019
		LE.stan:AM.stan	0.4381476	0.1427727	3.068.846	0.0040
Model:;PN.stan;~;HDI.stan;+;AM.stan;+;**NPv.stan**;+;**AM.stan:NPv.stan**	R^2=0.44		Value	Std.Error	t-value	p-value
	P<0.00001	(intercept)	-0.0954174	0.1228659	-0.776598	0.4419
		HDI.stan	0.5714055	0.1518151	3.763.826	0.0005
		AM.stan	0.0386920	0.1461590	0.264725	0.7925
		**NPv.stan**	0.4369156	0.1491749	2.928.881	0.0055
		**AM.stan:NPv.stan**	0.2778321	0.1205536	2.304.635	0.0263
Model:;PN.stan;~;HDI.stan;+;GDP.stan;+;LE.stan;+;AM.stan;+;**tta.stan**;+;HDI.stan:GDP.stan;+;HDI.stan:LE.stan;+;HDI.stan:AM.stan	R^2=0.65		Value	Std.Error	t-value	p-value
	P<0.00001	(intercept)	-0.2110841	0.1522429	-1.386.495	0.1739
		HDI.stan	0.4348831	0.1523897	2.853.756	0.0070
		GDP.stan	-0.2794428	0.2223083	-1.257.006	0.2166
		LE.stan	0.0088994	0.1949442	0.045651	0.9638
		AM.stan	-0.0996675	0.1860855	-0.535600	0.5954
		**tta.stan**	0.7550971	0.1611941	4.684.398	0.0000
		HDI.stan:GDP.stan	0.7342170	0.3592999	2.043.466	0.0482
		HDI.stan:LE.stan	0.5148444	0.1859563	2.768.631	0.0087
		HDI.stan:AM.stan	-0.8559487	0.3081262	-2.777.916	0.0085
Model:;PN.stan;~;HDI.stan;+;GDP.stan;+;LE.stan;+;**Nta.stan**;+;HDI.stan:GDP.stan;+;**HDI.stan:Nta.stan**;+;**LE.stan:Nta.stan**	R^2=0.64		Value	Std.Error	t-value	p-value
	P<0.00001	(intercept)	-0.3640637	0.1471103	-2.474.767	0.0179
		HDI.stan	0.4672436	0.1592958	2.933.181	0.0057
		GDP.stan	-0.4414550	0.2134209	-2.068.472	0.0454
		LE.stan	0.0856020	0.2153973	0.397414	0.6933
		**Nta.stan**	0.8741278	0.2032459	4.300.838	0.0001

		HDI.stan:GDP.stan	10.747.848	0.3715478	2.892.723	0.0063
		HDI.stan:Nta.stan	-0.8434598	0.2842463	-2.967.355	0.0052
		**LE.stan:Nta.stan**	0.4495059	0.1787524	2.514.684	0.0163
Model:;PN.stan;~;HDI.stan;+;GDP.stan;+;LE.stan;+;AM.stan;+;**Pta.stan**;+;HDI.stan:GDP.stan;+;HDI.stan:LE.stan;+;HDI.stan:AM.stan	R^2=0.64		Value	Std.Error	t-value	p-value
	P<0.00001	(intercept)	-0.2262567	0.1561126	-1.449.318	0.1557
		HDI.stan	0.4272946	0.1563508	2.732.923	0.0096
		GDP.stan	-0.2799132	0.2285176	-1.224.909	0.2284
		LE.stan	-0.0102890	0.1994804	-0.051579	0.9591
		AM.stan	-0.0903106	0.1901405	-0.474968	0.6376
		**Pta.stan**	0.7520208	0.1708475	4.401.708	0.0001
		HDI.stan:GDP.stan	0.7700573	0.3683406	2.090.612	0.0435
		HDI.stan:LE.stan	0.4589874	0.1892252	2.425.615	0.0203
		HDI.stan:AM.stan	-0.8042180	0.3143917	-2.558.013	0.0148
Model:;PN.stan;~;HDI.stan;+;LE.stan;+;**protta.stan**;+;**LE.stan:protta.stan**	R^2=0.50		Value	Std.Error	t-value	p-value
	P<0.00001	(intercept)	-0.1888300	0.1446601	-13.053.365	0.1991
		HDI.stan	0.3948760	0.1485818	26.576.329	0.0112
		LE.stan	0.1025399	0.2397346	0.4277226	0.6711
		**protta.stan**	0.4952301	0.1722442	28.751.633	0.0064
		**LE.stan:protta.stan**	0.2723123	0.1359657	20.028.018	0.0518
Model:;PN.stan;~;HDI.stan;+;GDP.stan;+;LE.stan;+;AM.stan;+;**kcalta.stan**;+;HDI.stan:GDP.stan;+;HDI.stan:AM.stan;+;LE.stan:AM.stan	R^2=0.62		Value	Std.Error	t-value	p-value
	P<0.00001	(intercept)	-0.3339338	0.1684989	-1.981.816	0.0550
		HDI.stan	0.6323571	0.1881880	3.360.241	0.0018
		GDP.stan	-0.4036143	0.2462559	-1.639.004	0.1097
		LE.stan	0.2010678	0.2211665	0.909124	0.3692
		AM.stan	-0.2617402	0.1992818	-1.313.418	0.1971
		**kcalta.stan**	0.8052725	0.2002107	4.022.126	0.0003
		HDI.stan:GDP.stan	0.7713137	0.3643135	2.117.170	0.0410
		HDI.stan:AM.stan	-0.6657614	0.2457941	-2.708.614	0.0102
		LE.stan:AM.stan	0.4345048	0.1549108	2.804.872	0.0080
Model:;PN.stan;~;HDI.stan;+;GDP.stan;+;LE.stan;+;AM.stan;+;**NPta.stan**;+;HDI.stan:LE.stan;+;HDI.stan:AM.stan;+;**GDP.stan:NPta.stan**	R^2=0.60		Value	Std.Error	t-value	p-value
	P<0.00001	(intercept)	0.0108243	0.1257966	0.086046	0.9319
		HDI.stan	0.4735820	0.1646308	2.876.631	0.0066
		GDP.stan	0.1177397	0.1665160	0.707078	0.4839
		LE.stan	0.0103282	0.2159396	0.047829	0.9621
		AM.stan	0.0136009	0.1793517	0.075834	0.9400
		**NPta.stan**	-0.6319380	0.1570600	-4.023.546	0.0003
		HDI.stan:LE.stan	0.5526633	0.1946445	2.839.347	0.0073
		HDI.stan:AM.stan	-0.6696981	0.2362507	-2.834.693	0.0074
		**GDP.stan:NPta.stan**	-0.3574149	0.1534442	-2.329.282	0.0254

Model:;PN.stan;~;HDI.stan;+;GDP.stan;+;LE.stan;+;AM.stan;+;**taa.stan**;+;HDI.stan:AM.stan;+;**HDI.stan:taa.stan**;+;**GDP.stan:taa.stan**;+;LE.stan:AM.stan;+;**LE.stan:taa.stan**;+;**AM.stan:taa.stan**	R^2=0.63		Value	Std.Error	t-value	p-value
	P<0.00001	(intercept)	-0.1348879	0.1486914	-0.907167	0.3707
		HDI.stan	11.632.257	0.2593931	4.484.413	0.0001
		GDP.stan	0.0808842	0.1826295	0.442887	0.6607
		LE.stan	-0.0230767	0.2581742	-0.089384	0.9293
		AM.stan	-0.0736022	0.1890938	-0.389237	0.6995
		**taa.stan**	-0.1411387	0.1702908	-0.828810	0.4130
		HDI.stan:AM.stan	-0.9759273	0.2756312	-3.540.699	0.0012
		**HDI.stan:taa.stan**	14.553.825	0.6307125	2.307.521	0.0272
		**GDP.stan:taa.stan**	0.5099323	0.2509796	2.031.768	0.0501
		LE.stan:AM.stan	10.201.525	0.2609201	3.909.827	0.0004
		**LE.stan:taa.stan**	-13.006.074	0.4461705	-2.915.046	0.0063
		**AM.stan:taa.stan**	-0.3977311	0.2199186	-1.808.538	0.0794
Model:;PN.stan;~;HDI.stan;+;GDP.stan;+;LE.stan;+;AM.stan;+;**Paa.stan**;+;HDI.stan:AM.stan;+;HDI.stan:Paa.stan;+;**GDP.stan:Paa.stan**;+;LE.stan:AM.stan;+;**LE.stan:Paa.stan**;+;**AM.stan:Paa.stan**	R^2=0.63		Value	Std.Error	t-value	p-value
	P<0.00001	(intercept)	-0.1326996	0.1467574	-0.904210	0.3722
		HDI.stan	11.683.348	0.2553079	4.576.179	0.0001
		GDP.stan	0.0812224	0.1795800	0.452291	0.6539
		LE.stan	-0.0220940	0.2537919	-0.087056	0.9311
		AM.stan	-0.0794840	0.1877954	-0.423248	0.6748
		**Paa.stan**	-0.1399075	0.1663847	-0.840867	0.4063
		HDI.stan:AM.stan	-0.9821030	0.2706533	-3.628.639	0.0009
		**HDI.stan:Paa.stan**	14.421.418	0.6258104	2.304.439	0.0274
		**GDP.stan:Paa.stan**	0.5104002	0.2453858	2.079.991	0.0451
		LE.stan:AM.stan	10.225.986	0.2548152	4.013.099	0.0003
		**LE.stan:Paa.stan**	-12.991.071	0.4274312	-3.039.337	0.0045
		**AM.stan:Paa.stan**	-0.3902265	0.2205237	-1.769.545	0.0858
Model:;PN.stan;~;HDI.stan;+;GDP.stan;+;LE.stan;+;AM.stan;+;**protaa.stan**;+;HDI.stan:AM.stan;+;**HDI.stan:protaa.stan**;+;**GDP.stan:protaa**.stan;+;LE.stan:AM.stan;+;**LE.stan:protaa.stan**;+;**AM.stan:protaa.stan**	R^2=0.64		Value	Std.Error	t-value	p-value
	P<0.00001	(intercept)	-0.1503467	0.1418294	-1.060.054	0.2966
		HDI.stan	11.264.726	0.2474345	4.552.608	0.0001
		GDP.stan	0.1164765	0.1727755	0.674149	0.5048
		LE.stan	0.0589436	0.2486129	0.237090	0.8140
		AM.stan	-0.1006479	0.1837955	-0.547608	0.5875
		**protaa.stan**	-0.1948133	0.1647919	-1.182.178	0.2453
		HDI.stan:AM.stan	-0.8870113	0.2651166	-3.345.740	0.0020
		**HDI.stan:protaa.stan**	12.329.446	0.6772968	1.820.390	0.0775
		**GDP.stan:protaa.stan**	0.5579916	0.2456445	2.271.541	0.0296
		LE.stan:AM.stan	0.9665206	0.2440893	3.959.700	0.0004
		**LE.stan:protaa.stan**	-11.911.612	0.4233627	-2.813.571	0.0081

		**AM.stan:protaa.stan**	-0.3789227	0.2166745	-1.748.810	0.0893
Model:;PN.stan;~;HDI.stan;+;GDP.stan;+;LE.stan;+;AM.stan;+;**kcalaa.stan**;+;HDI.stan:LE.stan;+;HDI.stan:AM.stan;+;GDP.stan:LE.stan;+;GDP.stan:AM.stan;+;**GDP.stan:kcalaa.stan**;+;**AM.stan:kcalaa.stan**	R^2=0.63		Value	Std.Error	t-value	p-value
	P<0.00001	(intercept)	-0.1323065	0.1894387	-0.6984133	0.4897
		HDI.stan	0.5782806	0.2121098	27.263.272	0.0101
		GDP.stan	0.4326594	0.2358096	18.347.827	0.0753
		LE.stan	-0.2444507	0.2675279	-0.9137394	0.3673
		AM.stan	0.1088392	0.2217522	0.4908144	0.6267
		**kcalaa.stan**	-0.4857825	0.1824641	-26.623.465	0.0118
		HDI.stan:LE.stan	0.9321913	0.3170463	29.402.369	0.0059
		HDI.stan:AM.stan	-10.461.094	0.3735075	-28.007.723	0.0083
		GDP.stan:LE.stan	-0.6396260	0.3678272	-17.389.307	0.0911
		GDP.stan:AM.stan	0.7298235	0.3703336	19.707.188	0.0569
		**GDP.stan:kcalaa.stan**	0.7806268	0.2810694	27.773.452	0.0089
		**AM.stan:kcalaa.stan**	-0.7311104	0.3144001	-23.254.138	0.0262
Model:;PN.stan;~;HDI.stan;+;GDP.stan;+;AM.stan;+;**NPaa.stan**;+;HDI.stan:GDP.stan;+;HDI.stan:AM.stan;+;**AM.stan:NPaa.stan**	R^2=0.53		Value	Std.Error	t-value	p-value
	P<0.00001	(intercept)	-0.0603987	0.1641613	-0.367923	0.7150
		HDI.stan	0.8523268	0.2020202	4.219.018	0.0001
		GDP.stan	-0.0621001	0.2442370	-0.254262	0.8007
		AM.stan	-0.1594064	0.2202526	-0.723743	0.4737
		**NPaa.stan**	0.1405126	0.2829865	0.496534	0.6224
		HDI.stan:GDP.stan	0.9407910	0.4355219	2.160.146	0.0371
		HDI.stan:AM.stan	-0.9458103	0.3239775	-2.919.370	0.0059
		**AM.stan:NPaa.stan**	-0.2648286	0.1345770	-1.967.860	0.0564
Model:;PN.stan;~;HDI.stan;+;GDP.stan;+;AM.stan;+;**talc.stan**;+;HDI.stan:GDP.stan;+;HDI.stan:AM.stan;+;**GDP.stan:talc.stan**	R^2=0.55		Value	Std.Error	t-value	p-value
	P<0.00001	(intercept)	0.0109698	0.1624251	0.067537	0.9465
		HDI.stan	0.5377259	0.1458153	3.687.720	0.0007
		GDP.stan	0.0114097	0.2224272	0.051296	0.9594
		AM.stan	-0.4030572	0.2357095	-1.709.974	0.0954
		**talc.stan**	0.4367089	0.1651136	2.644.900	0.0118
		HDI.stan:GDP.stan	11.681.478	0.4446576	2.627.072	0.0124
		HDI.stan:AM.stan	-0.7666301	0.2636766	-2.907.464	0.0061
		**GDP.stan:talc.stan**	-0.4070233	0.1434765	-2.836.865	0.0073
Model:;PN.stan;~;HDI.stan;+;GDP.stan;+;LE.stan;+;AM.stan;**+;Nalc.stan**;+;HDI.stan:AM.stan;+;**HDI.stan:Nalc.stan**;+;**LE.stan:Nalc.stan**	R^2=0.57		Value	Std.Error	t-value	p-value
	P<0.00001	(intercept)	0.0487604	0.1426366	0.341851	0.7344
		HDI.stan	10.680.893	0.2436150	4.384.333	0.0001
		GDP.stan	0.3698334	0.1552810	2.381.704	0.0225
		LE.stan	-0.5348410	0.2300474	-2.324.917	0.0257
		AM.stan	-0.1597347	0.1993989	-0.801081	0.4282

		**Nalc.stan**	-0.4247637	0.3359437	-1.264.390	0.2140
		HDI.stan:AM.stan	-0.3761464	0.1944592	-1.934.321	0.0607
		**HDI.stan:Nalc.sta**	10.254.349	0.4844927	2.116.513	0.0411
		**LE.stan:Nalc.sta**	-10.435.628	0.3483868	-2.995.414	0.0049
Model:;PN.stan;~;HDI.stan;+;GDP.stan;+;LE.stan;+;AM.stan;+;**Palc.stan**;+;HDI.stan:LE.stan;+;HDI.stan:AM.stan;+;**HDI.stan:Palc.stan**;+;**LE.stan:Palc.stan**	R^2=0.51		Value	Std.Error	t-value	p-value
	P<0.00001	(intercept)	-623.737	724.253	-0.8612136	0.3948
		HDI.stan	4.087.301	1.671.692	24.450.089	0.0195
		GDP.stan	0.34607	0.18053	19.169.699	0.0632
		LE.stan	-3.048.356	1.288.136	-23.664.861	0.0235
		AM.stan	0.04896	0.22060	0.2219506	0.8256
		**Palc.stan**	-4.126.467	4.903.487	-0.8415374	0.4056
		HDI.stan:LE.stan	0.65073	0.25335	25.684.732	0.0145
		HDI.stan:AM.stan	-0.68868	0.27967	-24.625.054	0.0187
		**HDI.stan:Palc.stan**	27.201.757	11.267.542	24.141.696	0.0210
		**LE.stan:Palc.stan**	-20.512.946	8.673.576	-23.649.929	0.0235
Model:;PN.stan;~;HDI.stan;+;GDP.stan;+;LE.stan;+;**protalc.stan**;+;**HDI.stan:protalc.stan**;+;**LE.stan:protalc.stan**	R^2=0.56		Value	Std.Error	t-value	p-value
	P<0.00001	(intercept)	-0.0771417	0.1186202	-0.650325	0.5193
		HDI.stan	0.8193496	0.1947485	4.207.219	0.0001
		GDP.stan	0.3413067	0.1438791	2.372.177	0.0227
		LE.stan	-0.4062438	0.1715527	-2.368.041	0.0229
		**protalc.stan**	-0.0761340	0.1205188	-0.631719	0.5313
		**HDI.stan:protalc.stan**	0.6076804	0.2226247	2.729.618	0.0095
		**LE.stan:protalc.stan**	-0.8809828	0.2336340	-3.770.781	0.0005
Model:;PN.stan;~;HDI.stan;+;GDP.stan;+;LE.stan;+;**kcalb.stan**;+;**HDI.stan:kcalb.stan**;+;**LE.stan:kcalb.stan**	R^2=0.59		Value	Std.Error	t-value	p-value
	P<0.00001	(intercept)	-0.0758011	0.1066990	-0.710420	0.4817
		HDI.stan	0.7200549	0.1585879	4.540.415	0.0001
		GDP.stan	0.3427050	0.1399071	2.449.518	0.0189
		LE.stan	-0.2768947	0.1485878	-1.863.509	0.0699
		**kcalb.stan**	-0.0391908	0.1166815	-0.335878	0.7388
		**HDI.stan:kcalb.stan**	0.4787050	0.1484559	3.224.560	0.0026
		**LE.stan:kcalb.stan**	-0.7803470	0.1831255	-4.261.270	0.0001
Model:;PN.stan;~;HDI.stan;+;AM.stan;+;**NPalc.stan**;+;**AM.stan:NPalc.stan**	R^2=0.46		Value	Std.Error	t-value	p-value
	P<0.00001	(intercept)	-0.1218230	0.11989378	-1.016.091	0.3155
		HDI.stan	0.5520884	0.14400660	3.833.772	0.0004
		AM.stan	0.0734651	0.14555677	0.504718	0.6165
		**NPalc.stan**	-0.4875580	0.17019262	-2.864.742	0.0066
		**AM.stan:NPalc.st**	-0.2867663	0.09031022	-3.175.347	0.0028
	R^2=0.80		Value	Std.Error	t-value	p-value

Model:;CN.stan;~;HDI.stan;+;GDP.stan;+;LE.stan;+;AM.stan;+;**tv.stan**;+;HDI.stan:AM.stan;+;**GDP.stan:tv.stan**;+;LE.stan:AM.stan;+;**AM.stan:tv.stan**	$P<0.00001$	(intercept)	-0.0875006	0.10247062	-0.853910	0.3988
		HDI.stan	0.6177771	0.13799500	4.476.808	0.0001
		GDP.stan	-0.2696355	0.12199767	-2.210.169	0.0335
		LE.stan	0.4359615	0.17424037	2.502.070	0.0170
		AM.stan	0.3724967	0.13538867	2.751.314	0.0092
		**tv.stan**	-0.0426616	0.08599173	-0.496113	0.6228
		HDI.stan:AM.stan	-0.4789569	0.12726868	-3.763.352	0.0006
		**GDP.stan:tv.stan**	0.5017713	0.12685694	3.955.410	0.0003
		LE.stan:AM.stan	0.5000418	0.11534475	4.335.193	0.0001
		**AM.stan:tv.stan**	-0.5089580	0.09757894	-5.215.860	0.0000
Model:;CN.stan;~;GDP.stan;+;LE.stan;+;AM.stan;+;**Nv.stan**;+;GDP.stan:LE.stan;+;GDP.stan:AM.stan;+;**GDP.stan:Nv.stan**;+;**AM.stan:Nv.stan**	$R^2=0.77$		Value	Std.Error	t-value	p-value
	$P<0.00001$	(intercept)	-0.0474761	0.1376367	-0.344938	0.7321
		GDP.stan	-0.0945545	0.1483205	-0.637501	0.5277
		LE.stan	0.6873133	0.1911000	3.596.615	0.0009
		AM.stan	0.4069036	0.1561814	2.605.326	0.0131
		**Nv.stan**	-0.0503851	0.1038859	-0.485004	0.6305
		GDP.stan:LE.stan	0.7129763	0.1794218	3.973.743	0.0003
		GDP.stan:AM.stan	-0.5154964	0.1612208	-3.197.455	0.0028
		**GDP.stan:Nv.stan**	0.5461277	0.1772061	3.081.879	0.0039
		**AM.stan:Nv.stan**	-0.4741462	0.1216746	-3.896.837	0.0004
Model:;CN.stan;~;HDI.stan;+;GDP.stan;+;LE.stan;+;AM.stan;+;**Pv.stan**;+;HDI.stan:LE.stan;+;HDI.stan:AM.stan;+;GDP.stan:LE.stan;+;**GDP.stan:Pv.stan**;+;AM.stan:Pv.stan	$R^2=0.82$		Value	Std.Error	t-value	p-value
	$P<0.00001$	(intercept)	-0.1529523	0.1248397	-1.225.190	0.2287
		HDI.stan	0.4427575	0.1182460	3.744.375	0.0006
		GDP.stan	-0.3194295	0.1381508	-2.312.180	0.0268
		LE.stan	0.5125712	0.1826473	2.806.344	0.0081
		AM.stan	0.4947313	0.1269330	3.897.578	0.0004
		**Pv.stan**	-0.0395265	0.1068275	-0.370003	0.7136
		HDI.stan:LE.stan	0.3092248	0.1609654	1.921.063	0.0629
		HDI.stan:AM.stan	-0.5347372	0.1651222	-3.238.432	0.0026
		GDP.stan:LE.stan	0.5350121	0.2155209	2.482.414	0.0180
		**GDP.stan:Pv.stan**	0.5183708	0.1613375	3.212.959	0.0028
		**AM.stan:Pv.stan**	-0.5102371	0.1069321	-4.771.600	0.0000
Model:;CN.stan;~;HDI.stan;+;LE.stan;+;AM.stan;+;**protv.stan**;+;HDI.stan:LE.stan;+;HDI.stan:AM.stan;+;**LE.stan:protv.stan**;+;**AM.stan:protv.stan**	$R^2=0.78$		Value	Std.Error	t-value	p-value
	$P<0.00001$	(intercept)	-0.0250616	0.09242456	-0.271158	0.7878
		HDI.stan	0.1807327	0.12258194	1.474.382	0.1488
		LE.stan	0.2602260	0.15002636	1.734.535	0.0911
		AM.stan	0.4145302	0.12495920	3.317.325	0.0020
		**protv.stan**	-0.4181290	0.10881683	-3.842.503	0.0005

		HDI.stan:LE.stan	0.6430023	0.14154444	4.542.759	0.0001
		HDI.stan:AM.stan	-0.6722702	0.17104015	-3.930.482	0.0004
		**LE.stan:protv.stan**	0.4644984	0.17417028	2.666.921	0.0113
		**AM.stan:protv.stan**	-0.4360249	0.11905270	-3.662.453	0.0008
Model:;CN.stan;~;HDI.stan;+;LE.stan;+;AM.stan;+;**kcalv.stan**;+;HDI.stan:LE.stan;+;HDI.stan:AM.stan;+;**LE.stan:kcalv.stan**;+;**AM.stan:kcalv.stan**	R^2=0.78		Value	Std.Error	t-value	p-value
	P<0.00001	(intercept)	-0.0250616	0.09242456	-0.271158	0.7878
		HDI.stan	0.1807327	0.12258194	1.474.382	0.1488
		LE.stan	0.2602260	0.15002636	1.734.535	0.0911
		AM.stan	0.4145302	0.12495920	3.317.325	0.0020
		**kcalv.stan**	-0.4181290	0.10881683	-3.842.503	0.0005
		HDI.stan:LE.stan	0.6430023	0.14154444	4.542.759	0.0001
		HDI.stan:AM.stan	-0.6722702	0.17104015	-3.930.482	0.0004
		**LE.stan:kcalv.stan**	0.4644984	0.17417028	2.666.921	0.0113
		**AM.stan:kcalv.stan**	-0.4360249	0.11905270	-3.662.453	0.0008
Model:;CN.stan;~;HDI.stan;+;LE.stan;+;AM.stan;+;**NPv.stan**;+;HDI.stan:LE.stan;+;HDI.stan:AM.stan;+;**HDI.stan:NPv.stan**;+;LE.stan:AM.stan;+;**AM.stan:NPv.stan**	R^2=0.77		Value	Std.Error	t-value	p-value
	P<0.00001	(intercept)	0.0720278	0.1026056	0.701987	0.4872
		HDI.stan	0.6699413	0.1619236	4.137.391	0.0002
		LE.stan	-0.1607721	0.1925329	-0.835037	0.4092
		AM.stan	0.3579040	0.1356261	2.638.903	0.0122
		**NPv.stan**	-0.0331400	0.1354621	-0.244644	0.8081
		HDI.stan:LE.stan	11.031.386	0.2771730	3.979.964	0.0003
		HDI.stan:AM.stan	-0.5271253	0.1769831	-2.978.394	0.0052
		**HDI.stan:NPv.stan**	-0.6103520	0.1815543	-3.361.815	0.0018
		LE.stan:AM.stan	-0.5987415	0.2420099	-2.474.037	0.0182
		**AM.stan:NPv.stan**	0.4441490	0.1175165	3.779.462	0.0006
Model:;CN.stan;~;HDI.stan;+;GDP.stan;+;LE.stan;+;AM.stan;+;**tta.stan**;+;HDI.stan:LE.stan;+;**HDI.stan:tta.stan**;+;GDP.stan:AM.stan;+;**GDP.stan:tta.stan**	R^2=0.71		Value	Std.Error	t-value	p-value
	P<0.00001	(intercept)	0.0062909	0.1624102	0.038734	0.9693
		HDI.stan	0.1475917	0.1313654	1.123.520	0.2687
		GDP.stan	-0.0958051	0.1978089	-0.484332	0.6311
		LE.stan	0.2225393	0.1913854	1.162.781	0.2526
		AM.stan	0.5415908	0.1836950	2.948.316	0.0056
		**tta.stan**	0.0765832	0.1622374	0.472044	0.6397
		HDI.stan:LE.stan	0.4689070	0.1371549	3.418.813	0.0016
		HDI.stan:tta.stan	-0.7534509	0.2580215	-2.920.109	0.0060
		GDP.stan:AM.stan	-0.3745941	0.1906744	-1.964.575	0.0572
		**GDP.stan:tta.stan**	0.5864157	0.2104235	2.786.836	0.0084
Model:;CN.stan;~;HDI.stan;+;GDP.stan;+;LE.stan;+;AM.stan;+;**Pta.stan**;+;HDI.stan:LE.stan;+;**HDI.stan:Pta.stan**;+;GDP.stan:AM.stan;+;**GDP.stan:Pta.stan**	R^2=0.71		Value	Std.Error	t-value	p-value
	P<0.00001	(intercept)	-0.0411143	0.1680814	-0.244610	0.8081
		HDI.stan	0.2001498	0.1336231	1.497.868	0.1429
		GDP.stan	-0.1426465	0.2073112	-0.688079	0.4958

		LE.stan	0.2559876	0.1910750	1.339.723	0.1887
		AM.stan	0.5323448	0.1842326	2.889.526	0.0065
		**Pta.stan**	0.0934620	0.1721594	0.542880	0.5906
		HDI.stan:LE.stan	0.5039380	0.1461233	3.448.716	0.0015
		**HDI.stan:Pta.stan**	-0.7893333	0.2708644	-2.914.127	0.0061
		GDP.stan:AM.stan	-0.3705779	0.1940129	-1.910.068	0.0641
		**GDP.stan:Pta.stan**	0.6472045	0.2297298	2.817.242	0.0078
Model:;CN.stan;~;HDI.stan;+;GDP.stan;+;LE.stan;+;AM.stan;+;**NPta.stan**;+;HDI.stan:LE.stan;+;HDI.stan:AM.stan;+;**HDI.stan:NPta.stan**;+;**GDP.stan:NPta.stan**	R^2=0.74		Value	Std.Error	t-value	p-value
	P<0.00001	(intercept)	0.0557525	0.1072506	0.519834	0.6064
		HDI.stan	0.2629493	0.1360900	1.932.172	0.0612
		GDP.stan	-0.0517469	0.1407744	-0.367587	0.7153
		LE.stan	0.1206218	0.1852264	0.651213	0.5190
		AM.stan	0.5468117	0.1476112	3.704.405	0.0007
		**NPta.stan**	0.0349486	0.1334152	0.261954	0.7949
		HDI.stan:LE.stan	0.4356354	0.1617710	2.692.914	0.0107
		HDI.stan:AM.stan	-0.5686147	0.1950698	-2.914.930	0.0061
		**HDI.stan:NPta.stan**	0.4367484	0.1512483	2.887.625	0.0065
		**GDP.stan:NPta.stan**	-0.4021614	0.1498152	-2.684.384	0.0109
Model:;CN.stan;~;HDI.stan;+;GDP.stan;+;LE.stan;+;AM.stan;+;**Naa.stan**;+;HDI.stan:GDP.stan;+;GDP.stan:LE.stan;+;**AM.stan:Naa.stan**	R^2=0.73		Value	Std.Error	t-value	p-value
	P<0.00001	(intercept)	-0.0231712	0.14530041	-0.159471	0.8742
		HDI.stan	0.4317143	0.12886859	3.350.035	0.0019
		GDP.stan	-0.0975721	0.16302169	-0.598522	0.5531
		LE.stan	0.5137596	0.20521271	2.503.547	0.0168
		AM.stan	0.3231014	0.16867813	1.915.491	0.0632
		**Naa.stan**	-0.0701298	0.09708982	-0.722319	0.4746
		HDI.stan:GDP.stan	-0.6011161	0.21828007	-2.753.875	0.0091
		GDP.stan:LE.stan	0.6959885	0.21679023	3.210.424	0.0027
		**AM.stan:Naa.stan**	-0.2447318	0.08448696	-2.896.681	0.0063
Model:;CN.stan;~;HDI.stan;+;GDP.stan;+;LE.stan;+;AM.stan;+;**kcalaa.stan**;+;HDI.stan:LE.stan;+;HDI.stan:AM.stan;+;**GDP.stan:kcalaa.stan**;+;**AM.stan:kcalaa.stan**	R^2=0.74		Value	Std.Error	t-value	p-value
	P<0.00001	(intercept)	-0.0188611	0.1145413	-0.164666	0.8701
		HDI.stan	0.2725310	0.1324542	2.057.549	0.0469
		GDP.stan	0.0253088	0.1322217	0.191412	0.8493
		LE.stan	0.2396923	0.1693580	1.415.300	0.1656
		AM.stan	0.4389673	0.1577143	2.783.308	0.0085
		**kcalaa.stan**	-0.1989140	0.1407320	-1.413.424	0.1661
		HDI.stan:LE.stan	0.5413155	0.1576330	3.434.025	0.0015
		HDI.stan:AM.stan	-0.5593551	0.1883052	-2.970.471	0.0053
		**GDP.stan:kcalaa.stan**	0.4593975	0.2075805	2.213.106	0.0333

		**AM.stan:kcalaa.stan**	-0.4970436	0.2310952	-2.150.817	0.0383
Model:;CN.stan;~;HDI.stan;+;GDP.stan;+;LE.stan;+;AM.stan;+;**NPaa.stan**;+;HDI.stan:AM.stan;+;GDP.stan:LE.stan	R^2=0.75		Value	Std.Error	t-value	p-value
	P<0.00001	(intercept)	-0.0564477	0.1292121	-0.436861	0.6647
		HDI.stan	0.6650516	0.1458764	4.559.007	0.0001
		GDP.stan	-0.2387000	0.1518432	-1.572.016	0.1242
		LE.stan	0.4172582	0.1931678	2.160.082	0.0371
		AM.stan	0.2882701	0.1556372	1.852.193	0.0718
		**NPaa.stan**	0.5297579	0.1372798	3.858.964	0.0004
		HDI.stan:AM.stan	-0.5289573	0.1370320	-3.860.100	0.0004
		GDP.stan:LE.stan	0.6228350	0.1861914	3.345.134	0.0019
Model:;CN.stan;~;HDI.stan;+;GDP.stan;+;LE.stan;+;AM.stan;+;**talc.stan**;+;HDI.stan:AM.stan;+;GDP.stan:LE.stan;+;**GDP.stan:talc.stan**	R^2=0.75		Value	Std.Error	t-value	p-value
	P<0.00001	(intercept)	-0.0605901	0.1397520	-0.433555	0.6671
		HDI.stan	0.4282442	0.1304439	3.282.977	0.0022
		GDP.stan	-0.3132238	0.1623489	-1.929.325	0.0614
		LE.stan	0.4977951	0.1992802	2.497.966	0.0171
		AM.stan	0.1269211	0.1819801	0.697445	0.4899
		**talc.stan**	0.3849855	0.1189593	3.236.279	0.0026
		HDI.stan:AM.stan	-0.3221113	0.1129674	-2.851.364	0.0071
		GDP.stan:LE.stan	0.5944173	0.1894035	3.138.366	0.0033
		**GDP.stan:talc.st**	-0.1874262	0.0988239	-1.896.567	0.0657
Model:;CN.stan;~;HDI.stan;+;GDP.stan;+;LE.stan;+;AM.stan;+;**Nalc.stan**;+;HDI.stan:GDP.stan;+;GDP.stan:LE.stan;+;LE.stan:AM.stan;+;**LE.stan:Nalc.stan**;+;AM.stan:Nalc.stan	R^2=0.75		Value	Std.Error	t-value	p-value
	P<0.00001	(intercept)	0.1345217	0.1576614	0.853232	0.3993
		HDI.stan	0.4342499	0.1335171	3.252.392	0.0025
		GDP.stan	-0.0991442	0.1632783	-0.607210	0.5476
		LE.stan	0.3532875	0.2062719	1.712.728	0.0956
		AM.stan	0.1122248	0.1966245	0.570757	0.5718
		**Nalc.stan**	-0.1724907	0.2524278	-0.683327	0.4989
		HDI.stan:GDP.stan	-0.7657581	0.2345531	-3.264.754	0.0025
		GDP.stan:LE.stan	10.559.983	0.3161081	3.340.624	0.0020
		LE.stan:AM.stan	-0.4486266	0.2036966	-2.202.425	0.0343
		**LE.stan:Nalc.stan**	0.6885986	0.3267289	2.107.554	0.0423
		**AM.stan:Nalc.stan**	-11.814.269	0.4520824	-2.613.300	0.0131
Model:;CN.stan;~;HDI.stan;+;LE.stan;+;AM.stan;+;**Palc.stan;**+;HDI.stan:LE.stan;+;HDI.stan:AM.stan;+;**HDI.stan:Palc.stan**	R^2=0.69		Value	Std.Error	t-value	p-value
	P<0.00001	(intercept)	834.453	4.574.809	1.824.018	0.0760
		HDI.stan	701.005	3.662.964	1.913.766	0.0632
		LE.stan	0.21385	0.178691	1.196.764	0.2388
		AM.stan	0.33714	0.155257	2.171.521	0.0362
		**Palc.stan**	5.671.567	31.176.051	1.819.206	0.0768

		HDI.stan:LE.stan	0.53932	0.160594	3.358.255	0.0018
		HDI.stan:AM.stan	-0.56856	0.193617	-2.936.506	0.0056
		**HDI.stan:Palc.st**	4.534.316	24.917.599	1.819.724	0.0767
Model:;CN.stan;~;HDI.stan;+;GDP.stan;+;LE.stan;+;AM.stan;+;**protalc.stan;**+;HDI.stan:AM.stan;+;GDP.stan:LE.stan	R^2=0.70		Value	Std.Error	t-value	p-value
	P<0.00001	(intercept)	-0.2271058	0.1345040	-1.688.468	0.0995
		HDI.stan	0.3686082	0.1406541	2.620.672	0.0125
		GDP.stan	-0.3597919	0.1696016	-2.121.394	0.0405
		LE.stan	0.5048417	0.2171726	2.324.610	0.0255
		AM.stan	0.4266065	0.1614547	2.642.268	0.0119
		**protalc.stan**	0.2653376	0.1078318	2.460.663	0.0185
		HDI.stan:AM.stan	-0.2429283	0.1177183	-2.063.641	0.0459
		GDP.stan:LE.stan	0.6470696	0.2057278	3.145.270	0.0032
Model:;CN.stan;~;HDI.stan;+;GDP.stan;+;LE.stan;+;AM.stan;+;**kcalb.stan;**+;HDI.stan:GDP.stan;+;HDI.stan:LE.stan;+;**HDI.stan:kcalb.stan;**+;GDP.stan:LE.stan;+;**GDP.stan:kcalb.stan;**+;LE.stan:AM.stan	R^2=0.76		Value	Std.Error	t-value	p-value
	P<0.00001	(intercept)	0.0257760	0.1515653	0.1700656	0.8660
		HDI.stan	0.2912210	0.1545259	18.846.099	0.0681
		GDP.stan	0.1126412	0.1967294	0.5725694	0.5707
		LE.stan	0.3721148	0.2279264	16.326.092	0.1118
		AM.stan	0.2059416	0.1951046	10.555.448	0.2986
		**kcalalc.stan**	0.1892385	0.1266567	14.941.059	0.1444
		HDI.stan:GDP.stan	-0.7738403	0.2920515	-26.496.711	0.0121
		HDI.stan:LE.stan	0.5119061	0.2616565	19.564.049	0.0587
		**HDI.stan:kcalalc.sta**	0.4042347	0.1426124	28.344.997	0.0077
		GDP.stan:LE.stan	0.6966325	0.2810498	24.786.804	0.0183
		**GDP.stan:kcalalc.st**	-0.2996664	0.1243624	-24.096.217	0.0215
		LE.stan:AM.stan	-0.4469949	0.2582418	-17.309.162	0.0925
Model:;CN.stan;~;HDI.stan;+;GDP.stan;+;LE.stan;+;AM.stan;+;**NPalc.stan**;+;HDI.stan:AM.stan;+;GDP.stan:LE.stan;+;**GDP.stan:NPalc.stan**;+;LE.stan:AM.stan;+;**AM.stan:NPalc.stan**	R^2=0.81		Value	Std.Error	t-value	p-value
	P<0.00001	(intercept)	-0.0664881	0.1220930	-0.544570	0.5895
		HDI.stan	0.8460745	0.1507302	5.613.172	0.0000
		GDP.stan	-0.2839843	0.1491581	-1.903.914	0.0652
		LE.stan	0.1342282	0.1829331	0.733755	0.4680
		AM.stan	0.2811219	0.1414179	1.987.881	0.0547
		**NPalc.stan**	0.0190453	0.1270882	0.149859	0.8817
		HDI.stan:AM.stan	-0.7037951	0.1429321	-4.923.983	0.0000
		GDP.stan:LE.stan	11.303.323	0.2676172	4.223.690	0.0002
		**GDP.stan:NPalc.sta**	0.7403152	0.2685732	2.756.474	0.0092
		LE.stan:AM.stan	-0.4412670	0.1951238	-2.261.472	0.0300
		**AM.stan:NPalc.sta**	-0.8556389	0.1910662	-4.478.234	0.0001
Model:;LN.stan;~;HDI.stan;+;GDP.stan;+;LE.stan;+;AM.stan;+;**tv.stan**;+;HDI.stan:LE.stan;+;**GDP.stan:tv.stan**;+;LE.stan:AM.stan;+;**LE.stan:tv.stan**	R^2=0.65		Value	Std.Error	t-value	p-value
	P<0.00001	(intercept)	-0.0599985	0.1357192	-0.442078	0.6611
		HDI.stan	0.5542588	0.2335274	2.373.420	0.0231

		GDP.stan	-0.0220229	0.1605711	-0.137154	0.8917
		LE.stan	-0.2238225	0.2596037	-0.862170	0.3943
		AM.stan	0.4787460	0.1769199	2.706.004	0.0103
		**tv.stan**	0.4721186	0.1199579	3.935.703	0.0004
		HDI.stan:LE.stan	-0.6429696	0.2752607	-2.335.857	0.0252
		**GDP.stan:tv.stan**	0.3726810	0.1526503	2.441.403	0.0197
		LE.stan:AM.stan	0.6091583	0.2470830	2.465.399	0.0186
		**LE.stan:tv.stan**	-0.2514190	0.1356377	-1.853.607	0.0720
Model:;LN.stan;~;HDI.stan;+;GDP.stan;+;LE.stan;+;AM.stan;+;**Nv.stan**;+;HDI.stan:LE.stan;+;HDI.stan:AM.stan;+;GDP.stan:LE.stan;+;**GDP.stan:Nv.stan**	R^2=0.60		Value	Std.Error	t-value	p-value
	P<0.00001	(intercept)	-0.3457971	0.1801066	-1.919.958	0.0628
		HDI.stan	0.1207517	0.1727801	0.698875	0.4891
		GDP.stan	-0.0462651	0.2011445	-0.230009	0.8194
		LE.stan	0.2117964	0.2558913	0.827681	0.4133
		AM.stan	0.7038053	0.1853212	3.797.759	0.0005
		**Nv.stan**	0.4962795	0.1564761	3.171.599	0.0031
		HDI.stan:LE.stan	-0.4394835	0.2270817	-1.935.354	0.0608
		HDI.stan:AM.stan	0.4392246	0.2401500	1.828.959	0.0757
		GDP.stan:LE.stan	0.5689555	0.2867951	1.983.840	0.0549
		**GDP.stan:Nv.stan**	0.3865748	0.1780863	2.170.716	0.0366
Model:;LN.stan;~;GDP.stan;+;AM.stan;+;**tta.stan**;+;**GDP.stan:tta.stan**	R^2=0.56		Value	Std.Error	t-value	p-value
	P<0.00001	(intercept)	-0.2889690	0.1467259	-1.969.448	0.0557
		GDP.stan	-0.1350474	0.1792292	-0.753490	0.4555
		AM.stan	0.7656251	0.1821146	4.204.084	0.0001
		**tta.stan**	0.2208522	0.1657588	1.332.371	0.1901
		**GDP.stan:tta.stan**	0.4162502	0.1507530	2.761.140	0.0086
Model:;LN.stan;~;GDP.stan;+;AM.stan;+;**Nta.stan;+;GDP.stan:Nta.stan**	R^2=0.60		Value	Std.Error	t-value	p-value
	P<0.00001	(intercept)	-0.3396460	0.1548384	-2.193.551	0.0340
		GDP.stan	-0.1821178	0.1797225	-1.013.328	0.3168
		AM.stan	0.7331647	0.1926352	3.805.976	0.0005
		**Nta.stan**	0.3569632	0.1877793	1.900.972	0.0644
		**GDP.stan:Nta.stan**	0.4716507	0.1626148	2.900.417	0.0060
Model:;LN.stan;~;GDP.stan;+;AM.stan;+;**Pta.stan;+;GDP.stan:Pta.stan**	R^2=0.57		Value	Std.Error	t-value	p-value
	P<0.00001	(intercept)	-0.3197493	0.1485922	-2.151.858	0.0373
		GDP.stan	-0.1614895	0.1778385	-0.908068	0.3691
		AM.stan	0.7711117	0.1827770	4.218.867	0.0001
		**Pta.stan**	0.2687863	0.1693985	1.586.710	0.1203
		**GDP.stan:Pta.stan**	0.4538885	0.1541087	2.945.248	0.0053
Model:;LN.stan;~;GDP.stan;+;AM.stan;+;**protta.stan;+;GDP.stan:protta.stan**	R^2=0.55		Value	Std.Error	t-value	p-value

	P<0.00001	(intercept)	-0.3571118	0.1724662	-2.070.619	0.0447
		GDP.stan	-0.2565871	0.2097959	-1.223.032	0.2283
		AM.stan	0.7366276	0.1885378	3.907.056	0.0003
		**protta.stan**	0.4386412	0.2061420	2.127.860	0.0394
		**GDP.stan:protta.stan**	0.4781551	0.1847829	2.587.659	0.0133
Model:;LN.stan;~;HDI.stan;+;GDP.stan;+;LE.stan;+;AM.stan;+;**kcalta.stan;**+;HDI.stan:LE.stan;+;GDP.stan:AM.stan;+;**GDP.stan:kcalta.stan;**+;LE.stan:AM.stan;+;**LE.stan:kcalta.stan;**+;**AM.stan:kcalta.stan**	R^2=0.67		Value	Std.Error	t-value	p-value
	P<0.00001	(intercept)	-0.5194216	0.1918011	-27.081.261	0.0105
		HDI.stan	0.3053813	0.1819827	16.780.783	0.1025
		GDP.stan	-0.2491314	0.2722975	-0.9149236	0.3667
		LE.stan	0.4043565	0.2334946	17.317.593	0.0924
		AM.stan	0.4028431	0.2231740	18.050.630	0.0799
		**kcalta.stan**	0.3629346	0.2463998	14.729.500	0.1500
		HDI.stan:LE.stan	-0.4192020	0.1875352	-22.353.238	0.0321
		GDP.stan:AM.stan	-0.8764899	0.3460595	-25.327.723	0.0161
		**GDP.stan:kcalta.stan**	0.8494011	0.3355772	25.311.643	0.0162
		LE.stan:AM.stan	11.156.577	0.3679451	30.321.308	0.0046
		**LE.stan:kcalta.stan**	-0.8465125	0.3929650	-21.541.679	0.0384
		**AM.stan:kcalta.stan**	0.6126572	0.3192473	19.190.680	0.0634
Model:;LN.stan;~;GDP.stan;+;AM.stan;+;**NPta.stan**;+;**GDP.stan:NPta.stan**	R^2=0.54		Value	Std.Error	t-value	p-value
	P<0.00001	(intercept)	-0.0380334	0.1062285	-0.358034	0.7222
		GDP.stan	0.0862204	0.1512789	0.569943	0.5718
		AM.stan	0.5808459	0.1438678	4.037.359	0.0002
		**NPta.stan**	-0.1322618	0.1487852	-0.888944	0.3792
		**GDP.stan:NPta.stan**	-0.3420314	0.1463316	-2.337.372	0.0244
Model:;LN.stan;~;LE.stan;+;AM.stan;+;**taa.stan**;+;LE.stan:AM.stan;+;**AM.stan:taa.stan**	R^2=0.57		Value	Std.Error	t-value	p-value
	P<0.00001	(intercept)	-0.0038968	0.1284950	-0.030327	0.9760
		LE.stan	0.3009866	0.2162548	1.391.814	0.1717
		AM.stan	0.5340893	0.1516977	3.520.747	0.0011
		**taa.stan**	0.0104709	0.1207715	0.086700	0.9313
		LE.stan:AM.stan	0.2170442	0.1065046	2.037.886	0.0482
		**AM.stan:taa.stan**	-0.4562271	0.1484210	-3.073.871	0.0038
Model:;LN.stan;~;HDI.stan;+;GDP.stan;+;LE.stan;+;AM.stan;+;**Naa.stan**;+;HDI.stan:LE.stan;+;HDI.stan:AM.stan;+;**GDP.stan:Naa.stan**;+;**LE.stan:Naa.stan**;+;**AM.stan:Naa.stan**	R^2=0.67		Value	Std.Error	t-value	p-value
	P<0.00001	(intercept)	0.1288426	0.1442764	0.893026	0.3779
		HDI.stan	-0.2676644	0.1862992	-1.436.745	0.1597
		GDP.stan	0.3173832	0.1499145	2.117.095	0.0414
		LE.stan	0.2459513	0.2546444	0.965862	0.3407
		AM.stan	0.3692519	0.1839052	2.007.838	0.0524
		**Naa.stan**	-0.0767739	0.1305783	-0.587953	0.5603
		HDI.stan:LE.stan	-0.4518345	0.2142774	-2.108.643	0.0422

		HDI.stan:AM.stan	0.5828378	0.2450271	2.378.667	0.0230
		**GDP.stan:Naa.stan**	-0.4215998	0.1650937	-2.553.700	0.0152
		**LE.stan:Naa.stan**	0.5252666	0.1919425	2.736.583	0.0097
		**AM.stan:Naa.stan**	-0.5876005	0.1850037	-3.176.156	0.0031
Model:;LN.stan;~;LE.stan;+;AM.stan;+;**Paa.stan**;+;LE.stan:AM.stan;+;**AM.stan:Paa.stan**	R^2=0.58		Value	Std.Error	t-value	p-value
	P<0.00001	(intercept)	-0.0033595	0.1276946	-0.026309	0.9791
		LE.stan	0.3001251	0.2145549	1.398.826	0.1696
		AM.stan	0.5352714	0.1504395	3.558.051	0.0010
		**Paa.stan**	0.0033170	0.1191379	0.027842	0.9779
		LE.stan:AM.stan	0.2137337	0.1054766	2.026.361	0.0494
		**AM.stan:Paa.stan**	-0.4632231	0.1471287	-3.148.422	0.0031
Model:;LN.stan;~;LE.stan;+;AM.stan;+;**protaa.stan**;+;LE.stan:AM.stan;+;**AM.stan:protaa.stan**	R^2=0.58		Value	Std.Error	t-value	p-value
	P<0.00001	(intercept)	-0.0169566	0.1270267	-0.133489	0.8945
		LE.stan	0.3045453	0.2129273	1.430.279	0.1604
		AM.stan	0.5545006	0.1493165	3.713.593	0.0006
		protaa.stan	0.0238812	0.1217890	0.196087	0.8455
		LE.stan:AM.stan	0.2105115	0.1051452	2.002.103	0.0521
		**AM.stan:protaa.stan**	-0.4321652	0.1390852	-3.107.199	0.0035
Model:;LN.stan;~;AM.stan;+;kcalaa.stan;+;**AM.stan:kcalaa.stan**	R^2=0.54		Value	Std.Error	t-value	p-value
	P<0.00001	(intercept)	0.1520567	0.1199234	1.267.949	0.2118
		AM.stan	0.5185154	0.1352409	3.834.013	0.0004
		**kcalaa.stan**	0.1033223	0.1562231	0.661377	0.5120
		**AM.stan:kcalaa.stan**	-0.5562551	0.2241473	-2.481.650	0.0172
Model:;LN.stan;~;HDI.stan;+;GDP.stan;+;LE.stan;+;AM.stan;+;**talc.stan**;+;HDI.stan:GDP.stan;+;HDI.stan:LE.stan;+;GDP.stan:AM.stan;+;**GDP.stan:talc.stan**;+;LE.stan:AM.stan	R^2=0.64		Value	Std.Error	t-value	p-value
	P<0.00001	(intercept)	-0.2421214	0.1736083	-13.946.419	0.1719
		HDI.stan	0.0791294	0.1889650	0.4187518	0.6780
		GDP.stan	-0.1289392	0.1946078	-0.6625594	0.5120
		LE.stan	0.3766948	0.2341173	16.090.005	0.1166
		AM.stan	0.3482982	0.2459071	14.163.812	0.1655
		**talc.stan**	0.3381082	0.1685861	20.055.521	0.0527
		HDI.stan:GDP.stan	0.7262173	0.3753663	19.346.898	0.0611
		HDI.stan:LE.stan	-0.5160559	0.2400440	-21.498.383	0.0386
		GDP.stan:AM.stan	-0.6298238	0.3213947	-19.596.582	0.0580
		**GDP.stan:talc.sta**	0.3255135	0.1707360	19.065.306	0.0648
		LE.stan:AM.stan	0.5913571	0.2360959	25.047.329	0.0171
Model:;LN.stan;~;LE.stan;+;AM.stan;+;**Nalc.stan**;+;**LE.stan:Nalc.stan**;+;**AM.stan:Nalc.stan**	R^2=0.54		Value	Std.Error	t-value	p-value
	P<0.00001	(intercept)	0.0843552	0.1227933	0.6869686	0.4961
		LE.stan	0.1782341	0.1750777	10.180.288	0.3148
		AM.stan	0.3827748	0.1958613	19.543.160	0.0577
		**Nalc.stan**	-0.2577796	0.3115958	-0.8272884	0.4130

		**LE.stan:Nalc.sta**	0.9592380	0.3939656	24.348.268	0.0195
		**AM.stan:Nalc.st**	-14.115.422	0.5249345	-26.889.871	0.0104
Model:;LN.stan;~;HDI.stan;+;GDP.stan;+;LE.stan;+;AM.stan;+;**Palc.stan**;+;HDI.stan:GDP.stan;+;HDI.stan:LE.stan;+;GDP.stan:AM.stan;+;**GDP.stan:Palc.stan**;+;LE.stan:AM.stan	R^2=0.63		Value	Std.Error	t-value	p-value
	P<0.00001	(intercept)	1.519.135	479.292	3.169.541	0.0032
		HDI.stan	0.06880	0.19062	0.360936	0.7203
		GDP.stan	1.179.787	368.527	3.201.359	0.0029
		LE.stan	0.37537	0.24006	1.563.649	0.1269
		AM.stan	0.35245	0.22135	1.592.249	0.1203
		**Palc.stan**	10.438.891	3.264.053	3.198.138	0.0029
		HDI.stan:GDP.stan	0.87634	0.37379	2.344.491	0.0249
		HDI.stan:LE.stan	-0.58745	0.22672	-2.591.093	0.0139
		GDP.stan:AM.stan	-0.66878	0.30868	-2.166.570	0.0372
		**GDP.stan:Palc.stan**	8.114.177	2.534.842	3.201.058	0.0029
		LE.stan:AM.stan	0.61394	0.23702	2.590.297	0.0139
Model:;LN.stan;~;HDI.stan;+;GDP.stan;+;LE.stan;+;AM.stan;+;p**rotalc.stan**;+;HDI.stan:GDP.stan;+;HDI.stan:LE.stan;+;GDP.stan:AM.stan;+;**GDP.stan:protalc.stan**;+;LE.stan:AM.stan	R^2=0.64		Value	Std.Error	t-value	p-value
	P<0.00001	(intercept)	-0.2451957	0.1701649	-14.409.294	0.1585
		HDI.stan	0.0588256	0.1913586	0.3074101	0.7604
		GDP.stan	-0.1484980	0.1940689	-0.7651816	0.4493
		LE.stan	0.4595873	0.2394466	19.193.726	0.0631
		AM.stan	0.4053662	0.2206096	18.374.823	0.0746
		**protalc.stan**	0.3026905	0.1268791	23.856.615	0.0226
		HDI.stan:GDP.stan	0.7837474	0.3754774	20.873.359	0.0442
		HDI.stan:LE.stan	-0.5670700	0.2283445	-24.833.973	0.0180
		GDP.stan:AM.stan	-0.6335391	0.3043810	-20.814.019	0.0448
		**GDP.stan:protalc.sta**	0.3117936	0.1334578	23.362.717	0.0253
		LE.stan:AM.stan	0.6620789	0.2377298	27.850.058	0.0086
Model:;LN.stan;~;GDP.stan;+;AM.stan;+;**NPalc.stan**;+;**GDP.stan:NPalc.stan**	R^2=0.58		Value	Std.Error	t-value	p-value
	P<0.00001	(intercept)	-0.1695969	0.1119119	-1.515.450	0.1373
		GDP.stan	-0.0337323	0.1487564	-0.226762	0.8217
		AM.stan	0.7052728	0.1459831	4.831.194	0.0000
		**NPalc.stan**	-0.3642633	0.1690022	-2.155.376	0.0371
		**GDP.stan:NPalc.st**	-0.5208196	0.1554696	-3.349.978	0.0017

Table S7. Relationships within countries between national total mortality from malignant neoplasms of the colon, prostate, breast, cervix and lung (period 1960-2010) and various traits of annual per capita intake during the same period. The bold type indicates statistical significance (*P*<0.01).

**Per capita national food intake (mean for 1960-2009)**	**National annual mortality from malignant neoplasms (100000 inhabitants y^{-1}) (mean for 1960-2009)**					
	**Total**	**Colon**	**Prostate**	**Breast**	**Cervix**	**Lung**
Total vegetable intake (Tv)	*R*=0.22<br>*P*=0.11	*R*=0.14<br>*P*=0.29	*R*=0.17<br>*P*=0.17	*R*=0.23<br>*P*=0.071	*R*=-0.11<br>*P*=0.37	***R*=0.26**<br>***P*=0.04**
Total N intake from vegetables (Nv)	***R*=0.39**<br>***P*=0.001**	***R*=0.34**<br>***P*=0.005**	*R*=0.15<br>*P*=0.26	***R*=0.32**<br>***P*=0.009**	*R*=-0.073<br>*P*=0.57	***R*=0.46**<br>***P*<0.0001**
Total P intake from vegetables (Pv)	***R*=0.28**<br>***P*=0.027**	*R*=0.21<br>*P*=0.11	*R*=0.057<br>*P*=0.65	*R*=0.20<br>*P*=0.11	*R*=-0.12<br>*P*=0.37	***R*=0.34**<br>***P*=0.005**
Total protein intake from vegetables (Protv)	*R*=0.012<br>*P*=0.93	R=-0.02<br>*P*=0.88	*R*=-0.18<br>*P*=0.15	*R*=-0.06<br>*P*=0.66	*R*=-0.22<br>*P*=0.09	*R*=0.09<br>*P*=0.50
Total kilocalories from vegetables (Kcalv)	*R*=-0.20<br>*P*=0.13	*R*=-0.20<br>*P*=0.12	*R*=-0.16<br>*P*=0.22	*R*=-0.16<br>*P*=0.20	*R*=-0.23<br>*P*=0.06	*R*=-15<br>*P*=0.24
Total intake of terrestrial animals (Tta)	***R*=0.78**<br>***P*<0.0001**	***R*=0.75**<br>***P*<0.0001**	***R*=0.78**<br>***P*<0.0001**	***R*=0.82**<br>***P*<0.0001**	*R*=0.17<br>*P*=0.17	***R*=0.77**<br>***P*<0.0001**
Total N intake from terrestrial animals (Nta)	***R*=0.82**<br>***P*<0.0001**	***R*=0.80**<br>***P*<0.0001**	***R*=0.77**<br>***P*<0.0001**	***R*=0.86**<br>***P*<0.0001**	*R*=0.17<br>*P*=0.18	***R*=0.80**<br>***P*<0.0001**
Total P intake from terrestrial animals (Pta)	***R*=0.79**<br>***P*<0.0001**	***R*=0.76**<br>***P*<0.0001**	***R*=0.77**<br>***P*<0.0001**	***R*=0.83**<br>***P*<0.0001**	*R*=0.16<br>*P*=0.20	***R*=0.78**<br>***P*<0.0001**
Total protein intake from terrestrial animals (Protta)	***R*=0.79**<br>***P*<0.0001**	***R*=0.78**<br>***P*<0.0001**	***R*=0.74**<br>***P*<0.0001**	***R*=0.82**<br>***P*<0.0001**	*R*=0.13<br>*P*=0.30	***R*=0.76**<br>***P*<0.0001**
Total kilocalories from terrestrial animals (Kcalta)	***R*=0.81**<br>***P*<0.0001**	***R*=0.80**<br>***P*<0.0001**	***R*=0.76**<br>***P*<0.0001**	***R*=0.84**<br>***P*<0.0001**	*R*=0.19<br>*P*=0.13	***R*=0.76**<br>***P*<0.0001**
Total intake of aquatic animals (Taa)	*R*=0.19<br>*P*=0.13	*R*=0.17<br>*P*=0.19	*R*=0.20<br>*P*=0.11	R=0.12<br>P=0.33	*R*=-0.02<br>*P*=0.86	*R*=0.13<br>*P*=0.31
Total N intake from aquatic animals (Naa)	*R*=0.20<br>*P*=0.12	*R*=0.18<br>*P*=0.15	*R*=0.20<br>*P*=0.11	*R*=0.13<br>*P*=0.32	*R*=-0.12<br>*P*=0.93	*R*=0.14<br>P=0.26
Total P intake from aquatic animals (Paa)	*R*=0.19<br>*P*=0.13	*R*=0.17<br>*P*=0.18	*R*=0.28<br>*P*=0.11	*R*=0.12<br>*P*=0.33	*R*=-0.03<br>*P*=0.81	*R*=0.13<br>*P*=0.30
Total protein intake from aquatic animals (Prtaa)	*R*=0.17<br>*P*=0.17	*R*=0.14<br>*P*=0.27	*R*=0.17<br>*P*=0.17	*R*=0.087<br>*P*=0.49	*R*=-0.007<br>*P*=0.96	*R*=0.10<br>*P*=0.41
Total kilocalories from aquatic animals (Kcalaa)	*R*=-0.05<br>*P*=0.69	*R*=0.06<br>*P*=0.64	*R*=0.05<br>*P*=0.70	*R*=-0.0046<br>*P*=0.97	*R*=-0.08<br>*P*=0.54	*R*=0.24<br>*P*=0.85
Ratio of intake of animal/vegetable foods (Tav)	***R*=0.75**<br>***P*<0.0001**	***R*=0.75**<br>***P*<0.0001**	***R*=0.74**<br>***P*<0.0001**	***R*=0.76**<br>***P*<0.0001**	***R*=0.26**<br>***P*=0.043**	***R*=0.71**<br>***P*<0.0001**
Ratio of N intake from animal/vegetable foods (Nav)	***R*=0.69**<br>***P*<0.0001**	***R*=0.66**<br>***P*<0.0001**	***R*=0.73**<br>***P*<0.0001**	***R*=0.71**<br>***P*<0.0001**	*R*=0.19<br>*P*=0.14	***R*=0.62**<br>***P*<0.0001**
Ratio of P intake from animal/vegetable foods (Pav)	***R*=0.68**<br>***P*<0.0001**	***R*=0.67**<br>***P*<0.0001**	***R*=0.74**<br>***P*<0.0001**	***R*=0.72**<br>***P*<0.0001**	*R*=0.18<br>*P*=0.15	***R*=0.64**<br>***P*<0.0001**
Ratio of protein intake from animal/vegetable foods (Protav)	***R*=0.74**<br>***P*<0.0001**	***R*=0.68**<br>***P*<0.0001**	***R*=0.74**<br>***P*<0.0001**	***R*=0.73**<br>***P*<0.0001**	*R*=0.16<br>*P*=0.21	***R*=0.73**<br>***P*<0.0001**
Ratio of kilocalories intake from animal/vegetable foods (Kcalav)	***R*=0.75**<br>***P*<0.0001**	***R*=0.78**<br>***P*<0.0001**	***R*=0.64**<br>***P*<0.0001**	***R*=0.73**<br>***P*<0.0001**	***R*=0.30**<br>***P*=0.015**	***R*=0.63**<br>***P*<0.0001**
N:P ratio of vegetable foods (NPv)	*R*=0.29<br>*P*=0.021	*R*=0.32<br>*P*=0.009	*R*=0.17<br>*P*=0.18	*R*=0.26<br>*P*=0.036	*R*=0.069<br>*P*=0.59	*R*=0.29<br>*P*=0.018
N:P ratio of terrestrial animal foods (NPta)	*R*=-0.16<br>*P*=0.21	*R*=-0.11<br>*P*=0.38	*R*=-0.30<br>*P*=0.015	*R*=-0.19<br>*P*=0.12	*R*=-0.11<br>*P*=0.37	*R*=-0.16<br>*P*=0.20
N:P ratio of aquatic animal foods (NPaa)	*R*=0.12<br>*P*=0.36	*R*=0.13<br>*P*=0.29	*R*=-0.019<br>*P*=0.88	*R*=0.035<br>*P*=0.78	*R*=0.33<br>*P*=0.008	*R*=0.11<br>*P*=0.39
Total intake of alcoholic beverages (Talc)	***R=0.79***<br>***P<0.0001***	***R=0.81***<br>***P<0.0001***	***R=0.65***<br>***P<0.0001***	***R=0.76***<br>***P<0.0001***	***R=0.28***<br>***P=0.027***	***R=0.76***<br>***P<0.0001***
Total N intake from alcoholic beverages (Nalc)	***R=0.32***<br>***P=0.008***	***R=0.29***<br>***P=0.021***	***R=0.14***<br>***P=0.26***	***R=0.18***<br>***P=0.14***	***R=0.13***<br>***P=0.28***	***R=0.30***<br>***P=0.015***
Total P intake from alcoholic beverages (Palc)	***R=0.78***<br>***P<0.0001***	***R=0.80***<br>***P<0.0001***	***R=0.63***<br>***P<0.0001***	***R=0.74***<br>***P<0.0001***	***R=0.27***<br>***P=0.027***	***R=0.75***<br>***P<0.0001***
Total kilocalories from alcoholic beverages (Kcalalc)	***R=0.63***<br>***P<0.0001***	***R=0.66***<br>***P<0.0001***	***R=0.48***<br>***P<0.0001***	***R=0.57***<br>***P<0.0001***	***R=0.31***<br>***P=0.011***	***R=0.63***<br>***P<0.0001***
Total protein intake from alcoholic beverages (Protalc)	***R=0.70***<br>***P<0.0001***	***R=0.74***<br>***P<0.0001***	***R=0.70***<br>***P<0.0001***	***R=0.66***<br>***P<0.0001***	***R=0.23***<br>***P=0.066***	***R=0.72***<br>***P<0.0001***

Table S8. Best linear models accounting for mortality from malignant neoplasms (total (TN), colon (CN), cervix (CEN), breast (BN), prostate (PN) and lung (LN) neoplasms as functions of national per capita wealth (using GDP), the human development index (HDI), mean age of the population (MA), Life Expectance at Birth (LE) and mean per capita intake of food from different sources.

**Total mortality from neoplasms for 1961-2010**						
**Model**	**Statistical results of the model**	**Independent factor statistics**				
Model: TN.stan ~ HDI.stan + GDP.stan + LE.stan + AM.stan + **tv.stan** + HDI.stan:GDP.stan + **HDI.stan:tv.stan** + GDP.stan:LE.stan + **AM.stan:tv.stan** + GDP.stan:AM.stan	R^2=0.91		Value	Std.Error	t-value	p-value
	P<0.00001	Intercept)	0.0589327	0.09376045	0.628545	0.5323
		HDI.stan	-0.0981361	0.13185788	-0.744257	0.4599
		GDP.stan	-0.0433188	0.14016506	-0.309055	0.7585
		LE.stan	0.2145502	0.10938533	1.961.416	0.0550
		AM.stan	0.8333139	0.08692328	9.586.774	0.0000
		**tv.stan**	0.0185118	0.04525648	0.409042	0.6841
		HDI.stan:GDP.stan	-0.5971184	0.13811547	-4.323.327	0.0001
		**HDI.stan:tv.stan**	0.2711534	0.08328127	3.255.875	0.0020
		GDP.stan:LE.stan	0.3865866	0.13460058	2.872.102	0.0058
		**AM.stan:tv.stan**	-0.2867602	0.07993676	-3.587.338	0.0007
		GDP.stan:AM.stan	0.1940336	0.09894471	1.961.031	0.0550
Model: TN.stan ~ HDI.stan + GDP.stan + LE.stan + AM.stan + **Nv.stan** + HDI.stan:GDP.stan + **HDI.stan:Nv.stan** + GDP.stan:LE.stan + **LE.stan:Nv.stan** + **AM.stan:Nv.stan**	R^2=0.90		Value	Std.Error	t-value	p-value
	P<0.00001	Intercept)	0.1392660	0.09323082	1.493.777	0.1411
		HDI.stan	-0.0450049	0.12393772	-0.363125	0.7179
		GDP.stan	0.0013626	0.13046897	0.010444	0.9917
		LE.stan	0.0650496	0.12351606	0.526649	0.6006
		AM.stan	0.9105486	0.10731793	8.484.590	0.0000
		**Nv.stan**	-0.0179262	0.05991328	-0.299202	0.7659
		HDI.stan:GDP.stan	-0.4060107	0.13248695	-3.064.533	0.0034
		**HDI.stan:Nv.stan**	0.3556469	0.10118322	3.514.880	0.0009
		GDP.stan:LE.stan	0.3083819	0.14521818	2.123.577	0.0383
		**LE.stan:Nv.stan**	-0.2107663	0.08979715	-2.347.138	0.0226
		**AM.stan:Nv.stan**	-0.1981157	0.08355735	-2.371.015	0.0213
Model: TN.stan ~ HDI.stan + GDP.stan + LE.stan + AM.stan + **Nv.stan** + HDI.stan:GDP.stan + **HDI.stan:Nv.stan** + GDP.stan:LE.stan + **LE.stan:Nv.stan** + **AM.stan:Nv.stan**	R^2=0.90		Value	Std.Error	t-value	p-value
	P<0.00001	Intercept)	0.1392660	0.09323082	1.493.777	0.1411
		HDI.stan	-0.0450049	0.12393772	-0.363125	0.7179
		GDP.stan	0.0013626	0.13046897	0.010444	0.9917
		LE.stan	0.0650496	0.12351606	0.526649	0.6006
		AM.stan	0.9105486	0.10731793	8.484.590	0.0000
		**Nv.stan**	-0.0179262	0.05991328	-0.299202	0.7659
		HDI.stan:GDP.stan	-0.4060107	0.13248695	-3.064.533	0.0034

			Value	Std.Error	t-value	p-value
		**HDI.stan:Nv.stan**	0.3556469	0.10118322	3.514.880	0.0009
		GDP.stan:LE.stan	0.3083819	0.14521818	2.123.577	0.0383
		**LE.stan:Nv.stan**	-0.2107663	0.08979715	-2.347.138	0.0226
		**AM.stan:Nv.stan**	-0.1981157	0.08355735	-2.371.015	0.0213
Model: TN.stan ~ HDI.stan + AM.stan + **Pv.stan** + **HDI.stan:Pv.stan** + **AM.stan:Pv.stan**	R^2=0.90		Value	Std.Error	t-value	p-value
	P<0.00001	Intercept)	0.0534974	0.04697437	1.138.863	0.2594
		HDI.stan	0.0057253	0.07817464	0.073237	0.9419
		AM.stan	0.9332584	0.08128163	11.481.787	0.0000
		**Pv.stan**	-0.0440421	0.04941427	-0.891282	0.3764
		**HDI.stan:Pv.stan**	0.2313476	0.07418030	3.118.721	0.0028
		**AM.stan:Pv.stan**	-0.2813063	0.08089583	-3.477.389	0.0010
Model: TN.stan ~ HDI.stan + GDP.stan + LE.stan + AM.stan + **protv.stan** + HDI.stan:GDP.stan + **HDI.stan:protv.stan** + GDP.stan:LE.stan + **LE.stan:protv.stan**	R^2=0.90		Value	Std.Error	t-value	p-value
	P<0.00001	Intercept)	0.0778195	0.08483965	0.917254	0.3630
		HDI.stan	-0.0230224	0.12344238	-0.186503	0.8527
		GDP.stan	-0.0271672	0.12909385	-0.210445	0.8341
		LE.stan	0.0587259	0.10783382	0.544596	0.5882
		AM.stan	0.9222267	0.09102574	10.131.493	0.0000
		**protv.stan**	-0.0778265	0.05498654	-1.415.374	0.1626
		HDI.stan:GDP.stan	-0.3458260	0.12272098	-2.817.986	0.0067
		**HDI.stan:protv.stan**	0.2279979	0.08181954	2.786.594	0.0073
		GDP.stan:LE.stan	0.2645927	0.12679542	2.086.769	0.0416
		**LE.stan:protv.stan**	-0.3100139	0.08235708	-3.764.265	0.0004
Model: TN.stan ~ HDI.stan + LE.stan + AM.stan + **kcalv.stan** + **HDI.stan:kcalv.stan** + **LE.stan:kcalv.stan**	R^2=0.89		Value	Std.Error	t-value	p-value
	P<0.00001	Intercept)	0.0375505	0.04465167	0.840966	0.4038
		HDI.stan	-0.0114355	0.10037487	-0.113928	0.9097
		LE.stan	-0.0249557	0.09805408	-0.254510	0.8000
		AM.stan	0.9470061	0.08776122	10.790.712	0.0000
		**kcalv.stan**	-0.0247695	0.07126585	-0.347565	0.7294
		**HDI.stan:kcalv.stan**	0.2260752	0.07333125	3.082.931	0.0031
		**LE.stan:kcalv.stan**	-0.3598372	0.14105989	-2.550.954	0.0134
Model: TN.stan ~ HDI.stan + GDP.stan + AM.stan + **tta.stan** + HDI.stan:GDP.stan + HDI.stan:AM.stan + **HDI.stan:tta.stan** + **GDP.stan:tta.stan**	R^2=0.91		Value	Std.Error	t-value	p-value
	P<0.00001	Intercept)	-0.0244809	0.08938807	-0.273872	0.7852
		HDI.stan	0.0574827	0.11692894	0.491604	0.6249
		GDP.stan	-0.1824492	0.14445565	-1.263.012	0.2118
		AM.stan	0.7555162	0.09117543	8.286.401	0.0000
		**tta.stan**	0.3128952	0.08578364	3.647.492	0.0006
		HDI.stan:GDP.stan	-0.2713100	0.13384596	-2.027.031	0.0474
		HDI.stan:AM.stan	0.3762604	0.11777835	3.194.648	0.0023
		**HDI.stan:tta.stan**	-0.3605036	0.11999470	-3.004.329	0.0040

		**GDP.stan:tta.stan**	0.2923917	0.10527891	2.777.306	0.0074
Model: TN.stan ~ HDI.stan + GDP.stan + AM.stan + **Nta.stan** + HDI.stan:GDP.stan + HDI.stan:AM.stan + **HDI.stan:Nta.stan + GDP.stan:Nta.stan**	R^2=0.91		Value	Std.Error	t-value	p-value
	P<0.00001	Intercept)	-0.0604609	0.09396556	-0.643437	0.5226
		HDI.stan	0.0880530	0.12313124	0.715115	0.4775
		GDP.stan	-0.1776757	0.15068919	-1.179.087	0.2433
		AM.stan	0.7236736	0.10140760	7.136.286	0.0000
		**Nta.stan**	0.3120991	0.09344849	3.339.799	0.0015
		HDI.stan:GDP.stan	-0.3537667	0.12544220	-2.820.157	0.0066
		HDI.stan:AM.stan	0.3960736	0.13251336	2.988.933	0.0042
		**HDI.stan:Nta.stan**	-0.2797950	0.12131120	-2.306.423	0.0248
		**GDP.stan:Nta.stan**	0.3314756	0.11013818	3.009.634	0.0039
Model: TN.stan ~ HDI.stan + GDP.stan + AM.stan + **Pta.stan** + HDI.stan:GDP.stan + HDI.stan:AM.stan + **HDI.stan:Pta.stan + GDP.stan:Pta.stan**	R^2=0.91		Value	Std.Error	t-value	p-value
	P<0.00001	Intercept)	-0.0355641	0.08988472	-0.395663	0.6939
		HDI.stan	0.0563867	0.11795297	0.478044	0.6345
		GDP.stan	-0.1776216	0.14443145	-1.229.799	0.2239
		AM.stan	0.7444835	0.09335627	7.974.649	0.0000
		**Pta.stan**	0.3214541	0.08896178	3.613.396	0.0006
		HDI.stan:GDP.stan	-0.3128623	0.13240693	-2.362.884	0.0216
		HDI.stan:AM.stan	0.3981341	0.12035978	3.307.867	0.0016
		**HDI.stan:Pta.stan**	-0.3484374	0.12004317	-2.902.601	0.0053
		**GDP.stan:Pta.stan**	0.3196282	0.10766417	2.968.752	0.0044
Model: TN.stan ~ HDI.stan + GDP.stan + AM.stan + **protta.stan** + HDI.stan:GDP.stan + GDP.stan:AM.stan	R^2=0.90		Value	Std.Error	t-value	p-value
	P<0.00001	Intercept)	-0.0298543	0.09363658	-0.318831	0.7510
		HDI.stan	0.0396740	0.11939355	0.332296	0.7409
		GDP.stan	-0.1964129	0.14202051	-1.382.989	0.1720
		AM.stan	0.8116674	0.08449069	9.606.590	0.0000
		**protta.stan**	0.2684935	0.08433254	3.183.747	0.0023
		HDI.stan:GDP.stan	-0.2791162	0.09996756	-2.792.068	0.0071
		GDP.stan:AM.stan	0.3302190	0.10639073	3.103.832	0.0030
Model: TN.stan ~ HDI.stan + GDP.stan + AM.stan + **kcalta.stan** + HDI.stan:GDP.stan + GDP.stan:AM.stan	R^2=0.89		Value	Std.Error	t-value	p-value
	P<0.00001	Intercept)	0.0084672	0.09376061	0.090307	0.9284
		HDI.stan	0.0418328	0.12140006	0.344587	0.7317
		GDP.stan	-0.1386285	0.14068557	-0.985378	0.3285
		AM.stan	0.7892805	0.08980777	8.788.555	0.0000
		**kcalta.stan**	0.2363845	0.08338025	2.835.018	0.0063
		HDI.stan:GDP.stan	-0.2735137	0.10153904	-2.693.680	0.0092
		GDP.stan:AM.stan	0.2746949	0.10325016	2.660.479	0.0101
Model: TN.stan ~ HDI.stan + AM.stan + **NPta.stan** + **HDI.stan:NPta.stan**	R^2=0.88		Value	Std.Error	t-value	p-value
	P<0.00001	Intercept)	0.0184344	0.04523472	0.407528	0.6851
		HDI.stan	0.0381103	0.07993330	0.476776	0.6353

		AM.stan	0.9016951	0.07914729	11.392.621	0.0000
		**NPta.stan**	-0.0124846	0.05472410	-0.228137	0.8203
		**HDI.stan:NPta.stan**	0.1069298	0.04807150	2.224.392	0.0299
Model: TN.stan ~ HDI.stan + GDP.stan + LE.stan + AM.stan + **taa.stan** + HDI.stan:GDP.stan + **HDI.stan:taa.stan** + GDP.stan:LE.stan + **LE.stan:taa.stan** + **AM.stan**:taa.stan	R^2=0.91		Value	Std.Error	t-value	p-value
	P<0.00001	Intercept)	0.1224907	0.08692095	1.409.220	0.1645
		HDI.stan	-0.0459265	0.12299173	-0.373412	0.7103
		GDP.stan	-0.0021235	0.12660597	-0.016772	0.9867
		LE.stan	0.2020052	0.12355788	1.634.904	0.1079
		AM.stan	0.8145322	0.08675735	9.388.625	0.0000
		**taa.stan**	-0.0835660	0.05797422	-1.441.434	0.1552
		HDI.stan:GDP.stan	-0.5355274	0.13429343	-3.987.741	0.0002
		**HDI.stan:taa.stan**	0.4191400	0.14755598	2.840.549	0.0063
		GDP.stan:LE.stan	0.5077566	0.14776832	3.436.167	0.0011
		**LE.stan:taa.stan**	-0.2729972	0.13464206	-2.027.577	0.0476
		**AM.stan:taa.stan**	-0.2404749	0.08970621	-2.680.694	0.0097
Model: TN.stan ~ HDI.stan + GDP.stan + LE.stan + AM.stan + **Naa.stan** + HDI.stan:GDP.stan + **HDI.stan:Naa.stan** + GDP.stan:LE.stan + **LE.stan:Naa.stan** + **AM.stan:Naa.stan**	R^2=0.91		Value	Std.Error	t-value	p-value
	P<0.00001	Intercept)	0.1240303	0.08523612	1.455.138	0.1514
		HDI.stan	-0.0418304	0.12008593	-0.348337	0.7289
		GDP.stan	-0.0061190	0.12462237	-0.049100	0.9610
		LE.stan	0.1891223	0.11740977	1.610.789	0.1131
		AM.stan	0.8276190	0.08393608	9.860.111	0.0000
		**Naa.stan**	-0.0883663	0.05567068	-1.587.304	0.1183
		HDI.stan:GDP.stan	-0.5095885	0.13129292	-3.881.309	0.0003
		**HDI.stan:Naa.stan**	0.3998720	0.13365172	2.991.896	0.0042
		GDP.stan:LE.stan	0.4758536	0.14570124	3.265.954	0.0019
		**LE.stan:Naa.stan**	-0.2533053	0.12692935	-1.995.640	0.0510
		**AM.stan:Naa.stan**	-0.2317518	0.08722071	-2.657.073	0.0103
Model: TN.stan ~ HDI.stan + GDP.stan + LE.stan + AM.stan + **Paa.stan** + HDI.stan:GDP.stan + **HDI.stan:Paa.stan** + GDP.stan:LE.stan + **LE.stan:Paa.stan** + **AM.stan**:Paa.stan	R^2=0.91		Value	Std.Error	t-value	p-value
	P<0.00001	Intercept)	0.1242430	0.08626934	1.440.176	0.1556
		HDI.stan	-0.0469729	0.12160396	-0.386277	0.7008
		GDP.stan	-0.0029540	0.12558417	-0.023522	0.9813
		LE.stan	0.2020345	0.12180642	1.658.653	0.1030
		AM.stan	0.8176487	0.08596673	9.511.222	0.0000
		**Paa.stan**	-0.0855289	0.05752282	-1.486.868	0.1429
		HDI.stan:GDP.stan	-0.5402168	0.13361017	-4.043.232	0.0002
		**HDI.stan:Paa.stan**	0.4332657	0.14635650	2.960.345	0.0046
		GDP.stan:LE.stan	0.5102985	0.14706737	3.469.828	0.0010

		**LE.stan:Paa.stan**	-0.2795821	0.13350498	-2.094.170	0.0410
		**AM.stan:Paa.stan**	-0.2452959	0.08850292	-2.771.614	0.0076
Model: TN.stan ~ HDI.stan + GDP.stan + LE.stan + AM.stan + protaa.stan + HDI.stan:GDP.stan + **HDI.stan:protaa.stan** + GDP.stan:LE.stan + **LE.stan:protaa.stan** + **AM.stan:protaa.stan**	R^2=0.91		Value	Std.Error	t-value	p-value
	P<0.00001	Intercept)	0.1118337	0.08663144	1.290.914	0.2022
		HDI.stan	0.0083324	0.12269511	0.067912	0.9461
		GDP.stan	-0.0294267	0.12513358	-0.235163	0.8150
		LE.stan	0.1756409	0.12347463	1.422.486	0.1606
		AM.stan	0.8057034	0.08497972	9.481.126	0.0000
		**protaa.stan**	-0.0693271	0.05988479	-1.157.675	0.2521
		HDI.stan:GDP.stan	-0.5093154	0.13110936	-3.884.661	0.0003
		**HDI.stan:protaa.stan**	0.4723231	0.16156275	2.923.466	0.0050
		GDP.stan:LE.stan	0.4939611	0.14476876	3.412.069	0.0012
		**LE.stan:protaa.stan**	-0.3080158	0.14548231	-2.117.205	0.0389
		**AM.stan:protaa.stan**	-0.2551388	0.09352516	-2.728.023	0.0086
Model: TN.stan ~ HDI.stan + GDP.stan + LE.stan + AM.stan + **kcalaa.stan** + HDI.stan:GDP.stan + GDP.stan:LE.stan + **GDP.stan:kcalaa.stan** + **LE.stan:kcalaa.stan**	R^2=0.91		Value	Std.Error	t-value	p-value
	P<0.00001	Intercept)	0.0521616	0.08588568	0.607338	0.5461
		HDI.stan	-0.1098192	0.12146116	-0.904150	0.3699
		GDP.stan	0.0164598	0.13161444	0.125061	0.9009
		LE.stan	0.1215155	0.12075132	1.006.329	0.3187
		AM.stan	0.8930287	0.08771699	10.180.795	0.0000
		**kcalaa.stan**	-0.1075745	0.04826062	-2.229.033	0.0299
		HDI.stan:GDP.stan	-0.3713897	0.13237381	-2.805.614	0.0069
		GDP.stan:LE.stan	0.3720270	0.14470157	2.570.995	0.0129
		**GDP.stan:kcalaa.stan**	0.2992601	0.12339017	2.425.316	0.0186
		**LE.stan:kcalaa.stan**	-0.3344732	0.13931586	-2.400.826	0.0198
Model: TN.stan ~ HDI.stan + AM.stan + **Nalc.stan** + **HDI.stan:Nalc.stan** + **AM.stan:Nalc.stan**	R^2=0.88		Value	Std.Error	t-value	p-value
	P<0.00001	Intercept)	0.0314672	0.05353037	0.587838	0.5589
		HDI.stan	0.0401453	0.07951541	0.504874	0.6155
		AM.stan	0.9068677	0.08229240	11.020.066	0.0000
		**Nalc.stan**	-0.0489670	0.05013139	-0.976774	0.3327
		**HDI.stan:Nalc.stan**	0.1440691	0.05719271	2.519.012	0.0145
		**AM.stan:Nalc.stan**	-0.1908857	0.09177179	-2.080.005	0.0419
Model: TN.stan ~ HDI.stan + GDP.stan + LE.stan + AM.stan + **NPalc.stan** + HDI.stan:GDP.stan + **HDI.stan:NPalc.stan** + GDP.stan:LE.stan + **AM.stan:NPalc.stan**	R^2=0.91		Value	Std.Error	t-value	p-value
	P<0.00001	Intercept)	-0.0187268	0.08474687	-0.220973	0.8259
		HDI.stan	0.0631477	0.12037058	0.524610	0.6020
		GDP.stan	-0.1187197	0.12863401	-0.922926	0.3601

		LE.stan	0.1066403	0.10249871	1.040.407	0.3027
		AM.stan	0.8120867	0.08572370	9.473.304	0.0000
		**NPalc.stan**	-0.1087624	0.04647347	-2.340.312	0.0229
		HDI.stan:GDP.stan	-0.2473655	0.12504196	-1.978.260	0.0529
		**HDI.stan:NPalc.stan**	0.1907061	0.07121111	2.678.038	0.0097
		GDP.stan:LE.stan	0.2441514	0.12525597	1.949.220	0.0564
		**AM.stan:NPalc.stan**	-0.3386263	0.08572903	-3.949.961	0.0002
Model: TN.stan ~ HDI.stan + GDP.stan + LE.stan + AM.stan + **ttav.stan** + HDI.stan:LE.stan + **HDI.stan:ttav.stan** + **GDP.stan:ttav.stan** + **AM.stan:ttav.stan**	R^2=0.90		Value	Std.Error	t-value	p-value
	P<0.00001	Intercept)	-0.1320585	0.09249758	-1.427.696	0.1590
		HDI.stan	0.0599505	0.10927090	0.548641	0.5855
		GDP.stan	-0.2796464	0.10823630	-2.583.666	0.0125
		LE.stan	0.1158928	0.10982253	1.055.273	0.2959
		AM.stan	0.8299690	0.09516742	8.721.147	0.0000
		**ttav.stan**	0.2147025	0.07919754	2.710.974	0.0089
		HDI.stan:LE.stan	0.2160416	0.09168432	2.356.364	0.0220
		**HDI.stan:ttav.stan**	-0.5767714	0.15461425	-3.730.390	0.0005
		**GDP.stan:ttav.stan**	0.2299441	0.11539895	1.992.601	0.0513
		**AM.stan:ttav.stan**	0.2800269	0.10448391	2.680.096	0.0097
Model: TN.stan ~ HDI.stan + GDP.stan + AM.stan + **Ntav.stan** + **HDI.stan:Ntav.stan** + **AM.stan:Ntav.stan**	R^2=0.90		Value	Std.Error	t-value	p-value
	P<0.00001	Intercept)	-0.0770490	0.06564560	-1.173.711	0.2453
		HDI.stan	0.1262085	0.09850764	1.281.205	0.2052
		GDP.stan	-0.2171610	0.09064499	-2.395.731	0.0198
		AM.stan	0.9100296	0.08001319	11.373.494	0.0000
		**Ntav.stan**	0.1423418	0.06831718	2.083.543	0.0416
		**HDI.stan:Ntav.stan**	-0.1714867	0.06957120	-2.464.910	0.0167
		**AM.stan:Ntav.stan**	0.3080511	0.08619742	3.573.785	0.0007
Model: TN.stan ~ HDI.stan + GDP.stan + AM.stan + **Ptav.stan** + **HDI.stan:Ptav.stan** + **AM.stan:Ptav.stan**	R^2=0.91		Value	Std.Error	t-value	p-value
	P<0.00001	Intercept)	-0.0461714	0.06525381	-0.707566	0.4820
		HDI.stan	0.0534580	0.09472443	0.564353	0.5747
		GDP.stan	-0.2217890	0.08855035	-2.504.665	0.0151
		AM.stan	0.9017300	0.07584717	11.888.775	0.0000
		**Ptav.stan**	0.2400742	0.06777015	3.542.477	0.0008
		**HDI.stan:Ptav.stan**	-0.2393254	0.06899975	-3.468.496	0.0010
		**AM.stan:Ptav.stan**	0.3364563	0.08798559	3.823.993	0.0003
Model: TN.stan ~ HDI.stan + GDP.stan + AM.stan + **prottav.stan** + HDI.stan:GDP.stan + **AM.stan:prottav.stan**	R^2=0.90		Value	Std.Error	t-value	p-value
	P<0.00001	Intercept)	-0.0361905	0.09121629	-0.396755	0.6930

		HDI.stan	0.0822666	0.11886766	0.692086	0.4916
		GDP.stan	-0.1125772	0.12855409	-0.875719	0.3848
		AM.stan	0.9137742	0.07988209	11.439.038	0.0000
		**prottav.stan**	0.0980381	0.06699616	1.463.339	0.1488
		HDI.stan:GDP.stan	-0.2027172	0.08880996	-2.282.596	0.0261
		**AM.stan:prottav.stan**	0.2972681	0.08312198	3.576.288	0.0007
Model: TN.stan ~ HDI.stan + GDP.stan + LE.stan + AM.stan + **kcaltav.stan** + HDI.stan:AM.stan + **HDI.stan:kcaltav.stan** + LE.stan:AM.stan + **LE.stan:kcaltav.stan** + **AM.stan:kcaltav.stan**	R^2=0.93		Value	Std.Error	t-value	p-value
	P<0.00001	Intercept)	-0.0322435	0.10495234	-0.307221	0.7599
		HDI.stan	-0.1902762	0.11586778	-1.642.184	0.1064
		GDP.stan	-0.2114863	0.08851379	-2.389.303	0.0204
		LE.stan	0.2208718	0.11847790	1.864.245	0.0677
		AM.stan	0.9861228	0.10459926	9.427.627	0.0000
		**kcaltav.stan**	0.1863664	0.11858053	1.571.644	0.1219
		HDI.stan:AM.stan	0.4773543	0.15277123	3.124.635	0.0029
		**HDI.stan:kcaltav.stan**	-11.002.414	0.18602115	-5.914.603	0.0000
		LE.stan:AM.stan	-0.3741700	0.15278967	-2.448.922	0.0176
		**LE.stan:kcaltav.stan**	0.8643404	0.17358610	4.979.318	0.0000
		**AM.stan:kcaltav.stan**	0.2580753	0.10234343	2.521.659	0.0147
Model: colon.stan ~ HDI.stan + AM.stan + **tv.stan** + **HDI.stan:tv.stan** + **AM.stan:tv.stan**	R^2=0.84		Value	Std.Error	t-value	p-value
	P<0.00001	Intercept)	0.0098886	0.05280229	0.187276	0.8521
		HDI.stan	0.0832289	0.09120434	0.912554	0.3652
		AM.stan	0.8365304	0.09173862	9.118.630	0.0000
		**tv.stan**	-0.0614767	0.05388950	-1.140.792	0.2586
		**HDI.stan:tv.stan**	0.1888798	0.09441110	2.000.610	0.0500
		**AM.stan:tv.stan**	-0.2242246	0.08947729	-2.505.938	0.0150
Model: colon.stan ~ HDI.stan + AM.stan + **Nv.stan** + **HDI.stan:Nv.stan** + **AM.stan:Nv.stan**	R^2=0.84		Value	Std.Error	t-value	p-value
	P=0.000060	Intercept)	0.0556468	0.05692957	0.977468	0.3323
		HDI.stan	0.0505446	0.09280660	0.544623	0.5881
		AM.stan	0.8826582	0.09959923	8.862.099	0.0000
		**Nv.stan**	-0.0272177	0.06138256	-0.443411	0.6591
		**HDI.stan:Nv.stan**	0.2256379	0.10211705	2.209.600	0.0310
		**AM.stan:Nv.stan**	-0.2598987	0.09842977	-2.640.448	0.0106
Model: colon.stan ~ HDI.stan + AM.stan + **Pv.stan** + **HDI.stan:Pv.stan** + **AM.stan:Pv.stan**	R^2=0.87		Value	Std.Error	t-value	p-value
	P=0.00057	Intercept)	0.0767535	0.05110144	1.501.982	0.1384
		HDI.stan	0.0083082	0.08504289	0.097694	0.9225
		AM.stan	0.9300869	0.08842285	10.518.626	0.0000

		**Pv.stan**	-0.1291197	0.05375570	-2.401.972	0.0195
		**HDI.stan:Pv.stan**	0.2379217	0.08069761	2.948.311	0.0046
		**AM.stan:Pv.stan**	-0.3510917	0.08800316	-3.989.535	0.0002
Model: colon.stan ~ HDI.stan + LE.stan + AM.stan + **protv.stan** + **HDI.stan:protv.stan +** **LE.stan:protv.stan**	R^2=0.84		Value	Std.Error	t-value	p-value
	P=0.00038	Intercept)	-0.0088004	0.05207558	-0.168992	0.8664
		HDI.stan	0.1171407	0.11361686	1.031.015	0.3068
		LE.stan	-0.1321931	0.11085856	-1.192.449	0.2379
		AM.stan	0.9168881	0.10561493	8.681.425	0.0000
		**protv.stan**	-0.1038932	0.06509734	-1.595.967	0.1159
		**HDI.stan:protv.stan**	0.2211417	0.09999956	2.211.427	0.0310
		**LE.stan:protv.stan**	-0.2916803	0.10215089	-2.855.387	0.0060
Model: colon.stan ~ AM.stan + **kcalv.stan**	R^2=0.83		Value	Std.Error	t-value	p-value
	P=0.0084	Intercept)	0.0000000	0.05184720	0.000000	10.000
		AM.stan	0.8938734	0.05245302	17.041.410	0.0000
		**kcalv.stan**	-0.1161767	0.05245302	-2.214.871	0.0305
Model: colon.stan ~ AM.stan + **Nta.stan**	R^2=0.83		Value	Std.Error	t-value	p-value
	P=0.00016	Intercept)	0.0000000	0.05193678	0.000000	10.000
		AM.stan	0.7435882	0.09082019	8.187.478	0.0000
		**Nta.stan**	0.1963705	0.09082019	2.162.191	0.0345
Model: colon.stan ~ AM.stan + **kcalta.stan**	R^2=0.83		Value	Std.Error	t-value	p-value
	P=0.00069	Intercept)	0.0000000	0.05200795	0.000000	10.000
		AM.stan	0.7483412	0.09024907	8.291.955	0.0000
		**kcalta.stan**	0.1912921	0.09024907	2.119.602	0.0381
Model: colon.stan ~ AM.stan + **Naa.stan**	R^2=0.83		Value	Std.Error	t-value	p-value
	P=0.00026	Intercept)	0.0000000	0.05212259	0.000000	10.000
		AM.stan	0.9390658	0.05523415	17.001.542	0.0000
		**Naa.stan**	-0.1132034	0.05523415	-2.049.517	0.0446
Model: colon.stan ~ AM.stan + **kcalaa.stan**	R^2=0.83		Value	Std.Error	t-value	p-value
	P=0.00053	Intercept)	0.0000000	0.05170811	0.000000	10.000
		AM.stan	0.9270861	0.05306713	17.470.064	0.0000
		**kcalaa.stan**	-0.1217781	0.05306713	-2.294.794	0.0251
Model: colon.stan ~ AM.stan + **talc.stan** + **AM.stan:talc.stan**	R^2=0.85		Value	Std.Error	t-value	p-value
	P<0.00001	Intercept)	-0.1354085	0.07815253	-1.732.619	0.0882

		AM.stan	0.7701148	0.08863356	8.688.749	0.0000
		**talc.stan**	0.1536654	0.09135588	1.682.053	0.0977
		**AM.stan:talc.stan**	0.1690665	0.07525159	2.246.684	0.0283
Model: colon.stan ~ AM.stan + **Palc.stan** + **AM.stan:Palc.stan**	R^2=0.84		Value	Std.Error	t-value	p-value
	P<0.00001	Intercept)	-0.1374199	0.07772563	-1.768.013	0.0821
		AM.stan	0.7908869	0.08766427	9.021.770	0.0000
		**Palc.stan**	0.1312412	0.08939331	1.468.132	0.1472
		**AM.stan:Palc.stan**	0.1726266	0.07443196	2.319.254	0.0237
Model: colon.stan ~ AM.stan + **protalc.stan**	R^2=0.83		Value	Std.Error	t-value	p-value
	P<0.00001	Intercept)	0.0000000	0.05177816	0.000000	10.000
		AM.stan	0.7784708	0.07632519	10.199.395	0.0000
		**protalc.stan**	0.1720994	0.07632519	2.254.818	0.0277
Model: colon.stan ~ HDI.stan + AM.stan + **NPalc.stan** + **HDI.stan:NPalc.stan** + **AM.stan:NPalc.stan**	R^2=0.86		Value	Std.Error	t-value	p-value
	P<0.00001	Intercept)	-0.0498159	0.05137163	-0.969716	0.3361
		HDI.stan	0.1113533	0.08734056	1.274.932	0.2073
		AM.stan	0.7245508	0.09041538	8.013.579	0.0000
		**NPalc.stan**	-0.1721251	0.05396802	-3.189.391	0.0023
		**HDI.stan:NPalc.stan**	0.1646492	0.08152126	2.019.709	0.0480
		**AM.stan:NPalc.stan**	-0.3465586	0.10058680	-3.445.369	0.0011
Model: colon.stan ~ AM.stan + **ttav.stan**	R^2=0.83		Value	Std.Error	t-value	p-value
	P<0.00001	Intercept)	0.0000000	0.05175950	0.000000	1.000
		AM.stan	0.7715620	0.07836934	9.845.202	0.000
		**ttav.stan**	0.1775473	0.07836934	2.265.520	0.027
Model: colon.stan ~ HDI.stan + AM.stan + **Ptav.stan** + **HDI.stan:Ptav.stan** + **AM.stan:Ptav.stan**	R^2=0.85		Value	Std.Error	t-value	p-value
	P<0.00001	Intercept)	-0.0370198	0.07680775	-0.481981	0.6316
		HDI.stan	0.0039909	0.10405231	0.038355	0.9695
		AM.stan	0.7889134	0.08969317	8.795.691	0.0000
		**Ptav.stan**	0.1707899	0.08531789	2.001.807	0.0499
		**HDI.stan:Ptav.stan**	-0.2116486	0.08639026	-2.449.912	0.0173
		**AM.stan:Ptav.stan**	0.2916134	0.10691609	2.727.498	0.0084
Model: colon.stan ~ GDP.stan + AM.stan + **prottav.stan** + **GDP.stan:prottav.stan** + **AM.stan:prottav.stan**	R^2=0.85		Value	Std.Error	t-value	p-value
	P<0.00001	Intercept)	-0.0777431	0.08037240	-0.967287	0.3373
		GDP.stan	0.0023649	0.10829531	0.021837	0.9827
		AM.stan	0.8219834	0.10026484	8.198.122	0.0000

		**prottav.stan**	0.1862204	0.08363500	2.226.584	0.0298
		**GDP.stan:prottav.stan**	-0.1925317	0.09692497	-1.986.399	0.0516
		**AM.stan:prottav.stan**	0.3243266	0.11365232	2.853.673	0.0060
Model: colon.stan ~ HDI.stan + GDP.stan + LE.stan + AM.stan + **kcaltav.stan** + **HDI.stan:kcaltav.stan** + GDP.stan:AM.stan + **GDP.stan:kcaltav.stan** + LE.stan:AM.stan + **LE.stan:kcaltav.stan** + **AM.stan:kcaltav.stan**	R^2=0.88		Value	Std.Error	t-value	p-value
	P<0.00001	Intercept)	-0.1068964	0.1339162	-0.798233	0.4283
		HDI.stan	-0.0832158	0.1620156	-0.513628	0.6096
		GDP.stan	-0.1591087	0.1547613	-1.028.091	0.3086
		LE.stan	0.2486849	0.1535834	1.619.218	0.1113
		AM.stan	0.8430422	0.1360165	6.198.088	0.0000
		**kcaltav.stan**	0.1546039	0.1512853	1.021.936	0.3115
		**HDI.stan:kcaltav.stan**	-0.4082695	0.1751464	-2.331.019	0.0236
		GDP.stan:AM.stan	0.4054134	0.1599631	2.534.418	0.0143
		**GDP.stan:kcaltav.stan**	-0.6365476	0.2218714	-2.868.994	0.0059
		LE.stan:AM.stan	-0.3305482	0.1663641	-1.986.896	0.0521
		**LE.stan:kcaltav.stan**	0.9086311	0.2387485	3.805.808	0.0004
		**AM.stan:kcaltav.stan**	0.2819259	0.1490796	1.891.110	0.0641
Model: lung.stan ~ HDI.stan + GDP.stan + AM.stan + **Nv.stan** + HDI.stan:GDP.stan + HDI.stan:AM.stan + **AM.stan:Nv.stan**	R^2=0.82		Value	Std.Error	t-value	p-value
	P<0.00001	Intercept)	0.1154163	0.11599904	0.994977	0.3240
		HDI.stan	0.0130350	0.15242112	0.085520	0.9321
		GDP.stan	0.1804506	0.17316934	1.042.047	0.3018
		AM.stan	0.7330519	0.12767907	5.741.363	0.0000
		**Nv.stan**	0.0671242	0.06605961	1.016.116	0.3139
		HDI.stan:GDP.stan	-0.4255512	0.16938126	-2.512.387	0.0148
		HDI.stan:AM.stan	0.3588933	0.15878844	2.260.198	0.0276
		**AM.stan:Nv.stan**	-0.1420006	0.06215792	-2.284.514	0.0261
Model: lung.stan ~ HDI.stan + GDP.stan + AM.stan + **Pv.stan** + HDI.stan:GDP.stan + HDI.stan:AM.stan + **AM.stan:Pv.stan**	R^2=0.82		Value	Std.Error	t-value	p-value
	P<0.00001	Intercept)	0.1096022	0.11687904	0.937740	0.3523
		HDI.stan	0.0021784	0.15339504	0.014201	0.9887
		GDP.stan	0.1955407	0.17826881	1.096.887	0.2773
		AM.stan	0.7375960	0.13044336	5.654.531	0.0000
		**Pv.stan**	0.0223797	0.06713176	0.333370	0.7401
		HDI.stan:GDP.stan	-0.4480662	0.17407016	-2.574.055	0.0127
		HDI.stan:AM.stan	0.3766920	0.16372880	2.300.707	0.0251
		**AM.stan:Pv.stan**	-0.1506853	0.07096894	-2.123.257	0.0381
Model: lung.stan ~ HDI.stan + LE.stan + AM.stan + **kcalv.stan** + **HDI.stan:kcalv.stan** + **LE.stan:kcalv.stan**	R^2=0.81		Value	Std.Error	t-value	p-value
	P<0.00001	Intercept)	0.0384520	0.05939878	0.647354	0.5200

		HDI.stan	0.0414575	0.13352567	0.310484	0.7573
		LE.stan	-0.1425798	0.13043840	-1.093.081	0.2789
		AM.stan	0.9595937	0.11674611	8.219.492	0.0000
		**kcalv.stan**	0.0225623	0.09480281	0.237992	0.8127
		**HDI.stan:kcalv.stan**	0.2354109	0.09755035	2.413.225	0.0190
		**LE.stan:kcalv.stan**	-0.3910647	0.18764772	-2.084.037	0.0416
Model: lung.stan ~ AM.stan + **tta.stan**	R^2=0.80		Value	Std.Error	t-value	p-value
	P<0.00001	Intercept)	0.0000000	0.05597247	0.000000	10.000
		AM.stan	0.7252064	0.08908786	8.140.351	0.0000
		**tta.stan**	0.2080181	0.08908786	2.334.977	0.0228
Model: lung.stan ~ AM.stan + **Nta.stan**	R^2=0.80		Value	Std.Error	t-value	p-value
	P<0.00001	Intercept)	0.0000000	0.05601888	0.000000	10.000
		AM.stan	0.7012143	0.09795843	7.158.285	0.0000
		**Nta.stan**	0.2263752	0.09795843	2.310.931	0.0242
Model: lung.stan ~ AM.stan + **Pta.stan**	R^2=0.80		Value	Std.Error	t-value	p-value
	P<0.00001	Intercept)	0.0000000	0.05577491	0.000000	10.000
		AM.stan	0.7131572	0.09060435	7.871.114	0.0000
		**Pta.stan**	0.2206512	0.09060435	2.435.327	0.0178
Model: lung.stan ~ HDI.stan + GDP.stan + AM.stan + **protta.stan** + HDI.stan:GDP.stan + HDI.stan:AM.stan	R^2=0.82		Value	Std.Error	t-value	p-value
	P<0.00001	Intercept)	0.0508662	0.1144422	0.444471	0.6584
		HDI.stan	-0.0910886	0.1550808	-0.587362	0.5592
		GDP.stan	0.1210901	0.1715581	0.705826	0.4831
		AM.stan	0.6920655	0.1254018	5.518.783	0.0000
		**protta.stan**	0.2531754	0.1090596	2.321.439	0.0238
		HDI.stan:GDP.stan	-0.3769657	0.1629909	-2.312.802	0.0243
		HDI.stan:AM.stan	0.3128959	0.1512860	2.068.241	0.0431
Model: lung.stan ~ HDI.stan + GDP.stan + LE.stan + AM.stan + **taa.stan** + HDI.stan:GDP.stan + **HDI.stan:taa.stan** + GDP.stan:LE.stan + **AM.stan:taa.stan**	R^2=0.85		Value	Std.Error	t-value	p-value
	P<0.00001	Intercept)	0.1318096	0.10643133	1.238.448	0.2208
		HDI.stan	-0.1454602	0.15266121	-0.952830	0.3448
		GDP.stan	0.1073441	0.16008142	0.670559	0.5053
		LE.stan	0.2125613	0.14664968	1.449.449	0.1529
		AM.stan	0.7856499	0.10915604	7.197.494	0.0000
		**taa.stan**	-0.2099879	0.06148867	-3.415.066	0.0012
		HDI.stan:GDP.stan	-0.4339533	0.15904371	-2.728.516	0.0085
		**HDI.stan:taa.stan**	0.2842791	0.10364366	2.742.851	0.0082

		GDP.stan:LE.stan	0.3504661	0.17681598	1.982.095	0.0525
		**AM.stan:taa.stan**	-0.4181772	0.11336124	-3.688.891	0.0005
Model: lung.stan ~ HDI.stan + AM.stan + **Naa.stan** + **HDI.stan:Naa.stan** + **AM.stan:Naa.stan**	R^2=0.84		Value	Std.Error	t-value	p-value
	P<0.00001	Intercept)	0.0304072	0.05480887	0.554787	0.5811
		HDI.stan	0.0544171	0.09145148	0.595038	0.5541
		AM.stan	0.8817105	0.09178229	9.606.543	0.0000
		**Naa.stan**	-0.1792752	0.05636956	-3.180.354	0.0023
		**HDI.stan:Naa.stan**	0.2994238	0.09799345	3.055.549	0.0034
		**AM.stan:Naa.stan**	-0.3941331	0.11083766	-3.555.950	0.0007
Model: lung.stan ~ HDI.stan + GDP.stan + LE.stan + AM.stan + **Paa.stan** + HDI.stan:GDP.stan + **HDI.stan:Paa.stan** + GDP.stan:LE.stan + **AM.stan:Paa.stan**	R^2=0.86		Value	Std.Error	t-value	p-value
	P<0.00001	Intercept)	0.1328327	0.10585476	1.254.858	0.2148
		HDI.stan	-0.1450715	0.15153522	-0.957345	0.3426
		GDP.stan	0.1043960	0.15906532	0.656309	0.5144
		LE.stan	0.2123238	0.14538391	1.460.435	0.1499
		AM.stan	0.7900362	0.10838786	7.288.973	0.0000
		**Paa.stan**	-0.2126783	0.06137504	-3.465.225	0.0010
		HDI.stan:GDP.stan	-0.4340080	0.15780887	-2.750.213	0.0080
		**HDI.stan:Paa.stan**	0.2898986	0.10313622	2.810.832	0.0068
		GDP.stan:LE.stan	0.3486898	0.17553404	1.986.451	0.0520
		**AM.stan:Paa.stan**	-0.4219554	0.11203772	-3.766.190	0.0004
Model: lung.stan ~ HDI.stan + GDP.stan + LE.stan + AM.stan + **protaa.stan** + HDI.stan:GDP.stan + **HDI.stan:protaa.stan** + GDP.stan:LE.stan + **AM.stan:protaa.stan**	R^2=0.85		Value	Std.Error	t-value	p-value
	P<0.00001	Intercept)	0.1033308	0.10685628	0.967008	0.3378
		HDI.stan	-0.0837219	0.15244249	-0.549203	0.5851
		GDP.stan	0.0547891	0.16038690	0.341606	0.7339
		LE.stan	0.1886738	0.14660098	1.286.989	0.2035
		AM.stan	0.7892604	0.10883345	7.252.002	0.0000
		**protaa.stan**	-0.2023161	0.06152508	-3.288.351	0.0018
		HDI.stan:GDP.stan	-0.3959372	0.15854146	-2.497.373	0.0155
		**HDI.stan:protaa.stan**	0.2886841	0.11145764	2.590.079	0.0123
		GDP.stan:LE.stan	0.3399642	0.17740297	1.916.339	0.0605
		**AM.stan:protaa.stan**	-0.4133443	0.11987399	-3.448.157	0.0011
Model: lung.stan ~ AM.stan + **kcalaa.stan**	R^2=0.83		Value	Std.Error	t-value	p-value
	P<0.00001	Intercept)	0.0000000	0.05496491	0.00000	10.000
		AM.stan	0.9162738	0.05640952	1.624.325	0.0000
		**kcalaa.stan**	-0.1590280	0.05640952	-281.917	0.0065

			Value	Std.Error	t-value	p-value
Model: lung.stan ~ GDP.stan + AM.stan + **talc.stan** + GDP.stan:AM.stan + **GDP.stan:talc.stan**	R^2=0.81		Value	Std.Error	t-value	p-value
	P<0.00001	Intercept)	0.0534127	0.09213369	0.579730	0.5643
		GDP.stan	-0.0008287	0.11333335	-0.007312	0.9942
		AM.stan	0.8449679	0.13113489	6.443.502	0.0000
		**talc.stan**	0.0430491	0.10633979	0.404826	0.6871
		GDP.stan:AM.stan	-0.2679352	0.13104282	-2.044.639	0.0454
		**GDP.stan:talc.stan**	0.2348864	0.10314242	2.277.302	0.0264
Model: lung.stan ~ HDI.stan + AM.stan + **NPalc.stan** + **HDI.stan:NPalc.stan** + **AM.stan:NPalc.stan**	R^2=0.81		Value	Std.Error	t-value	p-value
	P<0.00001	Intercept)	-0.0345864	0.05947499	-0.581529	0.5631
		HDI.stan	0.0229307	0.10111767	0.226772	0.8214
		AM.stan	0.7991233	0.10467751	7.634.145	0.0000
		**NPalc.stan**	-0.1246466	0.06248094	-1.994.953	0.0507
		**HDI.stan:NPalc.stan**	0.1872392	0.09438044	1.983.877	0.0519
		**AM.stan:NPalc.stan**	-0.3043240	0.11645337	-2.613.269	0.0114
Model: breast.stan ~ HDI.stan + AM.stan + **Pv.stan** + **HDI.stan:Pv.stan** + **AM.stan:Pv.stan**	R^2=0.79		Value	Std.Error	t-value	p-value
	P<0.00001	Intercept)	0.0644081	0.06627595	0.971817	0.3351
		HDI.stan	0.1295625	0.10804308	1.199.175	0.2353
		AM.stan	0.7756593	0.11595088	6.689.551	0.0000
		**Nv.stan**	-0.0176771	0.07146002	-0.247371	0.8055
		**HDI.stan:Nv.stan**	0.2582457	0.11888207	2.172.285	0.0339
		**AM.stan:Nv.stan**	-0.2990906	0.11458944	-2.610.106	0.0115
Model: breast.stan ~ HDI.stan + AM.stan + **Pv.stan** + **HDI.stan:Pv.stan** + **AM.stan:Pv.stan**	R^2=0.79		Value	Std.Error	t-value	p-value
	P<0.00001	Intercept)	0.0647535	0.06392730	1.012.924	0.3152
		HDI.stan	0.1025180	0.10638765	0.963627	0.3392
		AM.stan	0.8058412	0.11061595	7.285.036	0.0000
		**Pv.stan**	-0.0976581	0.06724775	-1.452.213	0.1517
		**HDI.stan:Pv.stan**	0.2282846	0.10095177	2.261.324	0.0274
		**AM.stan:Pv.stan**	-0.3115948	0.11009092	-2.830.341	0.0063
Model: breast.stan ~ HDI.stan + AM.stan + **Pv.stan** + **HDI.stan:Pv.stan** + **AM.stan:Pv.stan**	R^2=0.80		Value	Std.Error	t-value	p-value
	P<0.00001	Intercept)	0.06475	0.06393	1.013	0.31523
		HDI.stan	0.10252	0.10639	0.964	0.33917
		AM.stan	0.80584	0.11062	7.285	8.9e-10
		**Pv.stan**	-0.09766	0.06725	-1.452	0.15174
		**HDI.stan:Pv.stan**	0.22828	0.10095	2.261	0.02744
		**AM.stan:Pv.stan**	-0.31159	0.11009	-2.830	0.00635

Model	Fit	Term	Value	Std.Error	t-value	p-value
Model: breast.stan ~ AM.stan + **protv.stan** + **AM.stan:protv**.stan	R^2=0.77, P<0.00001	Intercept)	0.0026237	0.06102972	0.042990	0.9659
		AM.stan	0.8559585	0.06170141	13.872.593	0.0000
		**protv.stan**	-0.1370044	0.06972824	-1.964.834	0.0540
		**AM.stan:protv.stan**	-0.1521254	0.07332784	-2.074.593	0.0423
Model: breast.stan ~ HDI.stan + GDP.stan + AM.stan + **tta.stan** + HDI.stan:GDP.stan + GDP.stan:AM.stan	R^2=0.83, P<0.00001	Intercept)	-0.0505969	0.1179455	-0.428986	0.6695
		HDI.stan	0.0351164	0.1539160	0.228153	0.8203
		GDP.stan	-0.1380521	0.1775908	-0.777361	0.4401
		AM.stan	0.5545913	0.1069737	5.184.370	0.0000
		**tta.stan**	0.4685911	0.1000444	4.683.831	0.0000
		HDI.stan:GDP.stan	-0.2591582	0.1277367	-2.028.846	0.0471
		GDP.stan:AM.stan	0.3362607	0.1299016	2.588.581	0.0122
Model: breast.stan ~ AM.stan + **Nta.stan** + **AM.stan:Nta.stan**	R^2=0.83, P<0.00001	Intercept)	-0.1810763	0.08890193	-2.036.810	0.0460
		AM.stan	0.5247421	0.09139739	5.741.325	0.0000
		**Nta.stan**	0.4325701	0.09098216	4.754.450	0.0000
		AM.stan:Nta.stan	0.2250358	0.08977469	2.506.674	0.0149
Model: breast.stan ~ HDI.stan + GDP.stan + AM.stan + **Pta.stan** + HDI.stan:GDP.stan + GDP.stan:AM.stan	R^2=0.84, P<0.00001	Intercept)	-0.0571014	0.1158110	-0.493057	0.6238
		HDI.stan	0.0140324	0.1516277	0.092545	0.9266
		GDP.stan	-0.1476839	0.1743740	-0.846938	0.4005
		AM.stan	0.5381589	0.1055515	5.098.545	0.0000
		**Pta.stan**	0.5115169	0.1023101	4.999.673	0.0000
		HDI.stan:GDP.stan	-0.2839262	0.1261328	-2.251.009	0.0282
		GDP.stan:AM.stan	0.3705607	0.1289449	2.873.791	0.0057
Model: breast.stan ~ HDI.stan + LE.stan + AM.stan + **protta.stan** + HDI.stan:LE.stan + **HDI.stan:protta.stan** + LE.stan:AM.stan	R^2=0.84, P<0.00001	Intercept)	-0.1138119	0.09541444	-1.192.816	0.2379
		HDI.stan	-0.0807960	0.12261852	-0.658922	0.5126
		LE.stan	-0.0872227	0.13890051	-0.627951	0.5325
		AM.stan	0.6506451	0.12316587	5.282.673	0.0000
		**protta.stan**	0.4221936	0.11023416	3.829.970	0.0003
		HDI.stan:LE.stan	-0.4980064	0.14587218	-3.413.992	0.0012
		**HDI.stan:protta.stan**	0.3147091	0.12113732	2.597.953	0.0119
		LE.stan:AM.stan	0.3546761	0.14373979	2.467.487	0.0166

Model: breast.stan ~ AM.stan + **kcalta.stan**	R^2=0.80		Value	Std.Error	t-value	p-value
	P<0.00001	Intercept)	0.0000000	0.05583339	0.000000	1,00E+00
		AM.stan	0.5352265	0.09688734	5.524.215	0,00E+00
		**kcalta.stan**	0.4051670	0.09688734	4.181.837	1,00E-04
Model: breast.stan ~ HDI.stan + GDP.stan + LE.stan + AM.stan + **taa.stan** + HDI.stan:GDP.stan + GDP.stan:LE.stan + **AM.stan:taa.stan**	R^2=0.84		Value	Std.Error	t-value	p-value
	P<0.00001	Intercept)	0.0157600	0.10943946	0.144007	0.8860
		HDI.stan	-0.1359523	0.15902199	-0.854927	0.3962
		GDP.stan	0.1148071	0.16615941	0.690945	0.4925
		LE.stan	0.5275614	0.15161238	3.479.672	0.0010
		AM.stan	0.4951014	0.11358584	4.358.830	0.0001
		**taa.stan**	-0.2515239	0.06334888	-3.970.456	0.0002
		HDI.stan:GDP.stan	-0.4794669	0.16544061	-2.898.121	0.0054
		GDP.stan:LE.stan	0.6043045	0.17964150	3.363.947	0.0014
		**AM.stan:taa.stan**	-0.2324550	0.06864520	-3.386.326	0.0013
Model: breast.stan ~ HDI.stan + GDP.stan + LE.stan + AM.stan + **Naa.stan** + HDI.stan:GDP.stan + GDP.stan:LE.stan + **AM.stan:Naa.stan**	R^2=0.85		Value	Std.Error	t-value	p-value
	P<0.00001	Intercept)	0.0263084	0.10751340	0.244699	0.8076
		HDI.stan	-0.1224287	0.15488302	-0.790459	0.4326
		GDP.stan	0.1042801	0.16266811	0.641061	0.5241
		LE.stan	0.4922004	0.14468612	3.401.849	0.0012
		AM.stan	0.5299855	0.10953531	4.838.490	0.0000
		**Naa.stan**	-0.2543818	0.06121550	-4.155.512	0.0001
		HDI.stan:GDP.stan	-0.4526781	0.15989511	-2.831.094	0.0064
		GDP.stan:LE.stan	0.5664622	0.17176912	3.297.812	0.0017
		**AM.stan:Naa.stan**	-0.2278985	0.06612332	-3.446.567	0.0011
Model: breast.stan ~ HDI.stan + GDP.stan + LE.stan + AM.stan + **Paa.stan** + HDI.stan:GDP.stan + GDP.stan:LE.stan + **AM.stan:Paa.stan**	R^2=0.84		Value	Std.Error	t-value	p-value
	P<0.00001	Intercept)	0.0152804	0.10907984	0.140084	0.8891
		HDI.stan	-0.1313361	0.15818272	-0.830281	0.4099
		GDP.stan	0.1089213	0.16544456	0.658355	0.5130
		LE.stan	0.5257843	0.15068569	3.489.278	0.0010
		AM.stan	0.4977676	0.11296179	4.406.513	0.0000
		**Paa.stan**	-0.2498634	0.06310866	-3.959.257	0.0002
		HDI.stan:GDP.stan	-0.4769626	0.16451744	-2.899.161	0.0053
		GDP.stan:LE.stan	0.6035408	0.17864369	3.378.461	0.0013
		**AM.stan:Paa.stan**	-0.2320294	0.06892625	-3.366.342	0.0014
Model: breast.stan ~ HDI.stan + GDP.stan + LE.stan + AM.stan + **protaa.stan** + HDI.stan:GDP.stan + GDP.stan:LE.stan + **AM.stan:protaa.stan**	R^2=0.85		Value	Std.Error	t-value	p-value
	P<0.00001	Intercept)	-0.0127945	0.10671543	-0.119893	0.9050

		HDI.stan	-0.0955368	0.15321729	-0.623538	0.5355
		GDP.stan	0.0617369	0.16148642	0.382304	0.7037
		LE.stan	0.5199081	0.14623532	3.555.284	0.0008
		AM.stan	0.5061344	0.10975042	4.611.685	0.0000
		**protaa.stan**	-0.2456411	0.06103760	-4.024.423	0.0002
		HDI.stan:GDP.stan	-0.4593159	0.15922628	-2.884.674	0.0056
		GDP.stan:LE.stan	0.6112718	0.17392451	3.514.581	0.0009
		**AM.stan:protaa.stan**	-0.2266769	0.06398435	-3.542.692	0.0008
Model: breast.stan ~ LE.stan + AM.stan + **kcalaa.stan** + LE.stan:AM.stan + **AM.stan:kcalaa.stan**	R^2=0.81		Value	Std.Error	t-value	p-value
	P<0.00001	Intercept)	-0.1497149	0.10049762	-1.489.736	0.1416
		LE.stan	0.3744888	0.12772649	2.931.959	0.0048
		AM.stan	0.5977310	0.11523601	5.187.015	0.0000
		**kcalaa.stan**	-0.1990196	0.05788986	-3.437.901	0.0011
		LE.stan:AM.stan	0.2319873	0.11263556	2.059.627	0.0439
		**AM.stan:kcalaa.stan**	-0.1924401	0.08699256	-2.212.145	0.0308
Model: breast.stan ~ AM.stan + **Nalc.stan**	R^2=0.78		Value	Std.Error	t-value	p-value
	P<0.00001	Intercept)	0.0000000	0.05923315	0.000000	10.000
		AM.stan	0.9412697	0.06508992	14.461.067	0.0000
		**Nalc.stan**	-0.1911586	0.06508992	-2.936.839	0.0046
Model: breast.stan ~ HDI.stan + AM.stan + **NPalc.stan** + **HDI.stan:NPalc.stan** + **AM.stan:NPalc.stan**	R^2=0.85		Value	Std.Error	t-value	p-value
	P<0.00001	Intercept)	-0.0747403	0.05402731	-1.383.380	0.1718
		HDI.stan	0.2082336	0.09185568	2.266.965	0.0271
		AM.stan	0.5627591	0.09508945	5.918.208	0.0000
		**NPalc.stan**	-0.2747651	0.05675792	-4.841.001	0.0000
		**HDI.stan:NPalc.stan**	0.1849523	0.08573555	2.157.242	0.0351
		**AM.stan:NPalc.stan**	-0.4657179	0.10578669	-4.402.424	0.0000
Model: breast.stan ~ AM.stan + **ttav.stan**	R^2=0.78		Value	Std.Error	t-value	p-value
	P<0.00001	Intercept)	0.0000000	0.05952185	0.000000	10.000
		AM.stan	0.6755215	0.09012237	7.495.604	0.0000
		**ttav.stan**	0.2539720	0.09012237	2.818.079	0.0065
Model: breast.stan ~ AM.stan + **Ntav.stan**	R^2=0.79		Value	Std.Error	t-value	p-value
	P<0.00001	Intercept)	0.0000000	0.05814126	0.000000	10.000
		AM.stan	0.6964399	0.07712936	9.029.505	0.0000
		**Ntav.stan**	0.2593037	0.07712936	3.361.933	0.0013

Model	Fit	Term	Value	Std.Error	t-value	p-value
Model: breast.stan ~ HDI.stan + AM.stan + **Ptav.stan** + **HDI.stan:Ptav.stan** + **AM.stan:Ptav.stan**	*R*²=0.82	Intercept)	-0.0618284	0.08321729	-0.742975	0.4604
	P<0.00001	HDI.stan	0.0145859	0.11273538	0.129382	0.8975
		AM.stan	0.6567837	0.09717799	6.758.564	0.0000
		**Ptav.stan**	0.3031110	0.09243760	3.279.088	0.0017
		**HDI.stan:Ptav.stan**	-0.2469251	0.09359946	-2.638.104	0.0106
		**AM.stan:Ptav.stan**	0.3692284	0.11583815	3.187.450	0.0023
Model: breast.stan ~ HDI.stan + AM.stan + **prottav.stan** + **HDI.stan:prottav.stan** + **AM.stan:prottav.stan**	*R*²=0.81	Intercept)	-0.1122066	0.08531378	-1.315.223	0.1935
	P<0.00001	HDI.stan	0.0756589	0.11660399	0.648853	0.5190
		AM.stan	0.6422286	0.10100822	6.358.182	0.0000
		**prottav.stan**	0.2474049	0.09489862	2.607.044	0.0115
		**HDI.stan:prottav.stan**	-0.1958423	0.09725227	-2.013.756	0.0486
		**AM.stan:prottav.stan**	0.3808745	0.12551681	3.034.450	0.0036
Model: breast.stan ~ HDI.stan + LE.stan + AM.stan + **kcaltav.stan** + **HDI.stan:kcaltav.stan** + **LE.stan:kcaltav.stan**	*R*²=0.81	Intercept)	0.0351964	0.1042695	0.337552	0.7369
	P<0.00001	HDI.stan	-0.1863127	0.1584783	-1.175.635	0.2445
		LE.stan	0.2808627	0.1347219	2.084.758	0.0415
		AM.stan	0.6255556	0.1264909	4.945.459	0.0000
		**kcaltav.stan**	0.2905799	0.1293137	2.247.094	0.0285
		**HDI.stan:kcaltav.stan**	-0.5013448	0.1646299	-3.045.283	0.0035
		**LE.stan:kcaltav.stan**	0.5263115	0.1631482	3.225.972	0.0021
Model: cervix.stan ~ HDI.stan + GDP.stan + LE.stan + AM.stan + **tv.stan** + HDI.stan:GDP.stan + GDP.stan:LE.stan + GDP.stan:AM.stan + LE.stan:AM.stan + **LE.stan:tv.stan** + **AM.stan:tv.stan**	*R*²=0.43	Intercept)	0.1417910	0.2536644	0.558971	0.5785
	P<0.00001	HDI.stan	0.2332442	0.3252025	0.717227	0.4764
		GDP.stan	-10.633.853	0.3487210	-3.049.387	0.0036
		LE.stan	0.0409785	0.2991260	0.136994	0.8916
		AM.stan	0.5830348	0.2406565	2.422.685	0.0189
		**tv.stan**	-0.1279920	0.1156089	-1.107.112	0.2732
		HDI.stan:GDP.stan	-10.540.327	0.3479466	-3.029.294	0.0038
		GDP.stan:LE.stan	15.621.954	0.4663980	3.349.490	0.0015
		GDP.stan:AM.stan	0.7960696	0.2517662	3.161.940	0.0026
		LE.stan:AM.stan	-13.873.466	0.3664288	-3.786.129	0.0004
		LE.stan:tv.stan	0.6351499	0.2325048	2.731.771	0.0085
		AM.stan:tv.stan	-0.4473635	0.1999568	-2.237.301	0.0295
	*R*²=0.35		Value	Std.Error	t-value	p-value

Model: cervix.stan ~ HDI.stan + GDP.stan + LE.stan + AM.stan + **Nv.stan** + GDP.stan:AM.stan + LE.stan:AM.stan	*P*<0.00001	Intercept)	0.1176743	0.2474306	0.475585	0.6362
		HDI.stan	0.6321823	0.2972482	2.126.782	0.0378
		GDP.stan	-0.9208605	0.3106554	-2.964.250	0.0044
		LE.stan	-0.7120538	0.2916556	-2.441.420	0.0178
		AM.stan	10.145.478	0.2712906	3.739.708	0.0004
		**Nv.stan**	-0.3880023	0.1368866	-2.834.480	0.0063
		GDP.stan:AM.stan	0.6686124	0.2376466	2.813.473	0.0067
		LE.stan:AM.stan	-0.7921256	0.2422312	-3.270.122	0.0018
Model: cervix.stan ~ HDI.stan + GDP.stan + LE.stan + AM.stan + **Pv.stan** + HDI.stan:GDP.stan + GDP.stan:LE.stan + GDP.stan:AM.stan + LE.stan:AM.stan	R^2=0.41		Value	Std.Error	t-value	p-value
	P<0.00001	Intercept)	0.2186966	0.2563973	0.852960	0.3974
		HDI.stan	0.4529644	0.3154425	1.435.965	0.1567
		GDP.stan	-10.395.214	0.3468157	-2.997.331	0.0041
		LE.stan	-0.4283246	0.2789288	-1.535.605	0.1304
		AM.stan	0.9240941	0.2532103	3.649.513	0.0006
		**Pv.stan**	-0.3441157	0.1236761	-2.782.395	0.0074
		HDI.stan:GDP.stan	-0.6444020	0.3341019	-1.928.759	0.0589
		GDP.stan:LE.stan	0.9312895	0.4157780	2.239.872	0.0292
		GDP.stan:AM.stan	0.7103289	0.2525994	2.812.077	0.0068
		LE.stan:AM.stan	-11.477.895	0.3061478	-3.749.135	0.0004
Model: cervix.stan ~ LE.stan + AM.stan + **protv.stan** + LE.stan:AM.stan	R^2=0.20		Value	Std.Error	t-value	p-value
	P=0.0084	Intercept)	0.4700064	0.1989793	2.362.087	0.0214
		LE.stan	-0.5128222	0.2519612	-2.035.322	0.0462
		AM.stan	0.5640368	0.2260957	2.494.682	0.0154
		**protv.stan**	-0.2741612	0.1205226	-2.274.771	0.0265
		LE.stan:AM.stan	-0.5876973	0.2034804	-2.888.226	0.0054
Model: cervix.stan ~ HDI.stan + GDP.stan + LE.stan + **protta.stan** + HDI.stan:GDP.stan + GDP.stan:LE.stan + **GDP.stan:protta.stan + LE.stan:protta.stan**	R^2=0.24		Value	Std.Error	t-value	p-value
	P=0.012	Intercept)	0.2752641	0.2446116	1.125.311	0.2653
		HDI.stan	0.4382898	0.3399795	1.289.165	0.2026
		GDP.stan	-0.7738771	0.3634051	-2.129.516	0.0376
		LE.stan	-0.2390029	0.3121800	-0.765593	0.4471
		**protta.stan**	0.4328656	0.2406517	1.798.722	0.0775
		HDI.stan:GDP.stan	-0.7083743	0.3521457	-2.011.594	0.0491
		GDP.stan:LE.stan	11.121.012	0.4293019	2.590.488	0.0122
		**GDP.stan:protta.stan**	0.5803310	0.2879987	2.015.048	0.0487
		**LE.stan:protta.stan**	-11.739.365	0.3087997	-3.801.612	0.0004
	R^2=0.32		Value	Std.Error	t-value	p-value

Model: cervix.stan ~ HDI.stan + GDP.stan + LE.stan + AM.stan + **NPaa.stan** + GDP.stan:AM.stan + **GDP.stan:NPaa.stan** + LE.stan:AM.stan	*P*=0.0034	Intercept)	-0.0820330	0.2469928	-0.3321269	0.7410
		HDI.stan	0.6535953	0.3125597	20.911.055	0.0411
		GDP.stan	-0.9280599	0.3346973	-27.728.337	0.0075
		LE.stan	-0.3591488	0.3184765	-11.277.089	0.2643
		AM.stan	0.5096281	0.2763129	18.443.877	0.0704
		**NPaa.stan**	0.1509513	0.1348062	11.197.653	0.2676
		GDP.stan:AM.stan	0.7248977	0.2530319	28.648.469	0.0059
		**GDP.stan:NPaa.stan**	-0.2795235	0.1446209	-19.328.018	0.0583
		LE.stan:AM.stan	-0.6305036	0.2630146	-23.972.190	0.0199
Model: cervix.stan ~ HDI.stan + GDP.stan + LE.stan + AM.stan + **talc.stan** + GDP.stan:AM.stan + **LE.stan:talc.stan**	R^2=0.42		Value	Std.Error	t-value	p-value
	P<0.00001	Intercept)	0.0540569	0.2125222	0.254359	0.8001
		HDI.stan	0.6202490	0.2799442	2.215.617	0.0307
		GDP.stan	-0.7856438	0.2947320	-2.665.621	0.0100
		LE.stan	-0.4517050	0.2320405	-1.946.665	0.0565
		AM.stan	0.0659278	0.2465263	0.267427	0.7901
		**talc.stan**	0.6013634	0.1846538	3.256.708	0.0019
		GDP.stan:AM.stan	0.5933097	0.2134887	2.779.115	0.0074
		**LE.stan:talc.stan**	-0.7941957	0.1752437	-4.531.951	0.0000
Model: cervix.stan ~ HDI.stan + GDP.stan + LE.stan + AM.stan **+ Palc.stan** + GDP.stan:AM.stan + **LE.stan:Palc.stan**	R^2=0.40		Value	Std.Error	t-value	p-value
	P<0.00001	Intercept)	-0.0079703	0.2146603	-0.037130	0.9705
		HDI.stan	0.6526972	0.2866207	2.277.216	0.0265
		GDP.stan	-0.8150215	0.3016524	-2.701.856	0.0091
		LE.stan	-0.4168051	0.2378628	-1.752.292	0.0851
		AM.stan	0.1156121	0.2510620	0.460492	0.6469
		**Palc.stan**	0.5002723	0.1818323	2.751.284	0.0079
		GDP.stan:AM.stan	0.6014777	0.2190867	2.745.387	0.0081
		**LE.stan:Palc.stan**	-0.7141674	0.1741138	-4.101.728	0.0001
Model: cervix.stan ~ HDI.stan + GDP.stan + LE.stan + AM.stan + **protalc.stan** + GDP.stan:AM.stan + **LE.stan:protalc.stan**	R^2=0.88		Value	Std.Error	t-value	p-value
	P<0.00001	Intercept)	-0.1198291	0.2272575	-0.5272834	0.6000
		HDI.stan	0.6333723	0.3096958	20.451.436	0.0455
		GDP.stan	-0.9143019	0.3220356	-28.391.327	0.0063
		LE.stan	-0.2470854	0.2533664	-0.9752100	0.3336
		AM.stan	0.1810155	0.2502137	0.7234437	0.4724
		**protalc.stan**	0.4535344	0.1875861	24.177.397	0.0188
		GDP.stan:AM.stan	0.5866416	0.2379924	24.649.597	0.0167
		**LE.stan:protalc.stan**	-0.6058955	0.2090689	-28.980.659	0.0053

Model	Fit	Term	Value	Std.Error	t-value	p-value
Model: cervix.stan ~ HDI.stan + LE.stan + **kcalb.stan** + **HDI.stan:kcalb.stan** + **LE.stan:kcalb.stan**	R^2=0.34		Value	Std.Error	t-value	p-value
	P=0.00012	Intercept)	0.2567520	0.1294126	1.983.980	0.0519
		HDI.stan	0.2898286	0.2160870	1.341.259	0.1850
		LE.stan	-0.5034481	0.2310370	-2.179.080	0.0333
		kcalb.stan	0.3544334	0.1276016	2.777.656	0.0073
		HDI.stan:kcalb.stan	0.6752386	0.1939574	3.481.376	0.0009
		LE.stan:kcalb.stan	-11.984.570	0.2573217	-4.657.427	0.0000
Model: cervix.stan ~ HDI.stan + GDP.stan + LE.stan + AM.stan + **ttav.stan** + HDI.stan:GDP.stan + GDP.stan:LE.stan + GDP.stan:AM.stan + LE.stan:AM.stan + **LE.stan:ttav.stan**	R^2=0.45		Value	Std.Error	t-value	p-value
	P=0.00016	Intercept)	0.1793861	0.2575193	0.696593	0.4890
		HDI.stan	0.3712169	0.3086802	1.202.594	0.2344
		GDP.stan	-11.652.066	0.3429046	-3.398.049	0.0013
		LE.stan	-0.3277424	0.2646332	-1.238.478	0.2209
		AM.stan	0.2813279	0.2350094	1.197.092	0.2365
		**ttav.stan**	0.7290562	0.2122780	3.434.442	0.0011
		HDI.stan:GDP.stan	-0.8050211	0.3273853	-2.458.941	0.0172
		GDP.stan:LE.stan	13.939.411	0.4901029	2.844.180	0.0063
		GDP.stan:AM.stan	0.6991582	0.2616613	2.671.997	0.0099
		LE.stan:AM.stan	-0.8064674	0.2994721	-2.692.963	0.0094
		**LE.stan:ttav.stan**	-0.6051449	0.2393565	-2.528.216	0.0144
Model: cervix.stan ~ HDI.stan + GDP.stan + LE.stan + AM.stan + **Ntav.stan** + GDP.stan:AM.stan + LE.stan:AM.stan	R^2=0.35		Value	Std.Error	t-value	p-value
	P<0.00001	Intercept)	-0.0915782	0.2394884	-0.382391	0.7036
		HDI.stan	0.7243378	0.2990262	2.422.322	0.0186
		GDP.stan	-12.221.535	0.3283356	-3.722.269	0.0005
		LE.stan	-0.7486157	0.2994958	-2.499.587	0.0153
		AM.stan	0.6909681	0.2291100	3.015.880	0.0038
		**Ntav.stan**	0.5017698	0.1827542	2.745.599	0.0081
		GDP.stan:AM.stan	0.8772784	0.2440501	3.594.666	0.0007
		LE.stan:AM.stan	-0.7317680	0.2386849	-3.065.833	0.0033
Model: cervix.stan ~ HDI.stan + GDP.stan + LE.stan + AM.stan + **Ptav.stan** + HDI.stan:GDP.stan + GDP.stan:LE.stan + GDP.stan:AM.stan + LE.stan:AM.stan + **LE.stan:Ptav.stan**	R^2=0.44		Value	Std.Error	t-value	p-value
	P<0.00001	Intercept)	0.2617602	0.2632146	0.994474	0.3244
		HDI.stan	0.4116238	0.3131149	1.314.610	0.1942
		GDP.stan	-11.125.584	0.3463605	-3.212.140	0.0022
		LE.stan	-0.6340085	0.3032516	-2.090.701	0.0413
		AM.stan	0.5542625	0.2266409	2.445.553	0.0178
		**Ptav.stan**	0.7140411	0.2219446	3.217.204	0.0022
		HDI.stan:GDP.stan	-0.6824880	0.3334350	-2.046.840	0.0456
		GDP.stan:LE.stan	10.656.769	0.5165622	2.063.018	0.0439

		GDP.stan:AM.stan	0.7651144	0.2786206	2.746.079	0.0082
		LE.stan:AM.stan	-0.8762164	0.2998454	-2.922.227	0.0051
		**LE.stan:Ptav.stan**	-0.4985612	0.2241147	-2.224.581	0.0303
Model: cervix.stan ~ HDI.stan + GDP.stan + AM.stan + **kcaltav.stan** + **HDI.stan:kcaltav.stan** + GDP.stan:AM.stan	R^2=0.33		Value	Std.Error	t-value	p-value
	P=0.00053	Intercept)	-0.0186202	0.2474627	-0.075244	0.9403
		HDI.stan	0.1081767	0.3359194	0.322032	0.7486
		GDP.stan	-0.9713432	0.3118855	-3.114.422	0.0029
		AM.stan	0.1041985	0.2257907	0.461483	0.6462
		**kcaltav.stan**	0.8954455	0.2500705	3.580.772	0.0007
		**HDI.stan:kcaltav.stan**	-0.6850409	0.2524551	-2.713.516	0.0088
		GDP.stan:AM.stan	0.6381906	0.2288778	2.788.346	0.0072
Model: prostate.stan ~ AM.stan + **Nv.stan**	R^2=0.64		Value	Std.Error	t-value	p-value
	P<0.00001	Intercept)	0.0000000	0.07545234	0.000000	10.000
		AM.stan	0.8803950	0.08503135	10.353.769	0.0000
		**Nv.stan**	-0.2467873	0.08503135	-2.902.309	0.0051
Model: prostate.stan ~ AM.stan + **Pv.stan** + **AM.stan:Pv.stan**	R^2=0.68		Value	Std.Error	t-value	p-value
	P<0.00001	Intercept)	0.0720118	0.07743049	0.930019	0.3560
		AM.stan	0.8512915	0.07777790	10.945.159	0.0000
		**Pv.stan**	-0.2909461	0.08001419	-3.636.181	0.0006
		**AM.stan:Pv.stan**	-0.2047186	0.08057310	-2.540.781	0.0136
Model: prostate.stan ~ LE.stan + AM.stan + **protv.stan** + **LE.stan:protv.stan**	R^2=0.56		Value	Std.Error	t-value	p-value
	P<0.00001	Intercept)	-0.0376282	0.06962363	-0.540451	0.5909
		LE.stan	0.1752730	0.12354979	1.418.642	0.1612
		AM.stan	0.6435762	0.12251513	5.253.034	0.0000
		**protv.stan**	-0.3434934	0.08658035	-3.967.336	0.0002
		**LE.stan:protv.stan**	-0.2747273	0.07339804	-3.742.979	0.0004
Model: prostate.stan ~ HDI.stan + GDP.stan + AM.stan + **tta.stan** + **HDI.stan:tta.stan** + **GDP.stan:tta.stan**	R^2=0.73		Value	Std.Error	t-value	p-value
	P<0.00001	Intercept)	-0.0829123	0.1181526	-0.701739	0.4856
		HDI.stan	-0.0883266	0.1674983	-0.527328	0.6000
		GDP.stan	-0.2877084	0.1857205	-1.549.147	0.1268
		AM.stan	0.6060206	0.1449212	4.181.724	0.0001
		**tta.stan**	0.5006082	0.1341052	3.732.950	0.0004
		**HDI.stan:tta.stan**	-0.4004765	0.1466727	-2.730.410	0.0084
		**GDP.stan:tta.stan**	0.5264194	0.1649152	3.192.060	0.0023

Model	Fit	Term	Value	Std.Error	t-value	p-value
Model: prostate.stan ~ HDI.stan + GDP.stan + AM.stan + **Nta.stan** + **HDI.stan:Nta.stan** + **GDP.stan:Nta.stan**	R^2=0.71<br>P<0.00001	Intercept)	-0.1627252	0.1273259	-1.278.022	0.2063
		HDI.stan	0.0018697	0.1735110	0.010775	0.9914
		GDP.stan	-0.3305496	0.2022316	-1.634.511	0.1076
		AM.stan	0.5777247	0.1559891	3.703.622	0.0005
		**Nta.stan**	0.4741199	0.1452912	3.263.238	0.0018
		**HDI.stan:Nta.stan**	-0.3219326	0.1404122	-2.292.768	0.0255
		**GDP.stan:Nta.stan**	0.5591979	0.1773417	3.153.224	0.0026
Model: prostate.stan ~ HDI.stan + GDP.stan + AM.stan + **Pta.stan** + **HDI.stan:Pta.stan** + **GDP.stan:Pta.stan**	R^2=0.72<br>P<0.00001	Intercept)	-0.0920507	0.1215009	-0.757613	0.4517
		HDI.stan	-0.0883354	0.1697275	-0.520454	0.6047
		GDP.stan	-0.2836394	0.1893848	-1.497.688	0.1396
		AM.stan	0.6051857	0.1488282	4.066.338	0.0001
		**Pta.stan**	0.4927810	0.1398227	3.524.329	0.0008
		**HDI.stan:Pta.stan**	-0.4012564	0.1467622	-2.734.058	0.0083
		**GDP.stan:Pta.stan**	0.5426929	0.1714735	3.164.878	0.0025
Model: prostate.stan ~ GDP.stan + AM.stan + **protta.stan** + GDP.stan:AM.stan	R^2=0.68<br>P<0.00001	Intercept)	-0.2838547	0.1261011	-2.251.009	0.0281
		GDP.stan	-0.2725587	0.1726796	-1.578.407	0.1197
		AM.stan	0.5384492	0.1367790	3.936.635	0.0002
		**protta.stan**	0.5198905	0.1422238	3.655.439	0.0005
		GDP.stan:AM.stan	0.3679346	0.1341252	2.743.217	0.0080
Model: prostate.stan ~ GDP.stan + AM.stan + **kcalta.stan** + GDP.stan:AM.stan	R^2=0.68<br>P<0.00001	Intercept)	-0.2089940	0.1218805	-1.714.746	0.0916
		GDP.stan	-0.1645250	0.1620455	-1.015.302	0.3140
		AM.stan	0.4846969	0.1454250	3.332.969	0.0015
		**kcalta.stan**	0.4774439	0.1400357	3.409.443	0.0012
		GDP.stan:AM.stan	0.2708995	0.1265673	2.140.359	0.0364
Model: prostate.stan ~ HDI.stan + GDP.stan + AM.stan + **NPta.stan** + HDI.stan:AM.stan + GDP.stan:AM.stan + **GDP.stan:NPta.stan**	R^2=0.71<br>P<0.00001	Intercept)	-0.1198522	0.14858918	-0.806601	0.4232
		HDI.stan	0.0389173	0.19260147	0.202061	0.8406
		GDP.stan	-0.2824647	0.21509419	-1.313.214	0.1944
		AM.stan	0.8486318	0.14519890	5.844.616	0.0000
		**NPta.stan**	-0.3704660	0.09681574	-3.826.506	0.0003
		HDI.stan:AM.stan	-0.3397462	0.16596135	-2.047.140	0.0453
		GDP.stan:AM.stan	0.4576101	0.18192440	2.515.386	0.0147

		**GDP.stan:NPta.stan**	-0.2073046	0.10225127	-2.027.404	0.0473
Model: prostate.stan ~ HDI.stan + GDP.stan + LE.stan + AM.stan + **taa.stan** + HDI.stan:LE.stan + GDP.stan:LE.stan	R^2=0.63		Value	Std.Error	t-value	p-value
	P<0.00001	Intercept)	-0.2356411	0.14015166	-1.681.329	0.0982
		HDI.stan	-0.1038460	0.19167355	-0.541786	0.5901
		GDP.stan	-0.2833692	0.23284324	-1.216.995	0.2286
		LE.stan	0.5808254	0.18697711	3.106.398	0.0030
		AM.stan	0.5638955	0.15369749	3.668.866	0.0005
		**taa.stan**	-0.1733088	0.08691055	-1.994.105	0.0509
		HDI.stan:LE.stan	-0.3532438	0.17081262	-2.068.019	0.0432
		GDP.stan:LE.stan	0.7392927	0.26006100	2.842.767	0.0062
Generalized least squares fit by maximum likelihood Model: prostate.stan ~ HDI.stan + GDP.stan + LE.stan + AM.stan + **Naa.stan** + HDI.stan:LE.stan + GDP.stan:LE.stan	R^2=0.69		Value	Std.Error	t-value	p-value
	P<0.00001	Intercept)	-0.2359658	0.13859785	-1.702.521	0.0941
		HDI.stan	-0.1080773	0.19021229	-0.568193	0.5721
		GDP.stan	-0.2908326	0.23111090	-1.258.411	0.2134
		LE.stan	0.5878967	0.18409565	3.193.430	0.0023
		AM.stan	0.5764702	0.15218087	3.788.059	0.0004
		**Naa.stan**	-0.1907680	0.08576451	-2.224.323	0.0301
		HDI.stan:LE.stan	-0.3581286	0.16943625	-2.113.648	0.0389
		GDP.stan:LE.stan	0.7454409	0.25709843	2.899.438	0.0053
Model: prostate.stan ~ HDI.stan + GDP.stan + LE.stan + AM.stan + **Paa.stan** + HDI.stan:LE.stan + GDP.stan:LE.stan	R^2=0.68		Value	Std.Error	t-value	p-value
	P<0.00001	Intercept)	-0.2486559	0.2036168	-12.211.953	0.2279
		HDI.stan	-0.1536443	0.2548461	-0.6028907	0.5494
		GDP.stan	-0.2345984	0.3031239	-0.7739358	0.4427
		LE.stan	0.6244839	0.2472938	25.252.712	0.0148
		AM.stan	0.5330941	0.1923341	27.717.086	0.0079
		**Paa.stan**	-0.1746442	0.1145823	-15.241.807	0.1339
		HDI.stan:GDP.stan	0.0437739	0.3823883	0.1144750	0.9093
		HDI.stan:LE.stan	-0.2737366	0.3397035	-0.8058104	0.4242
		HDI.stan:AM.stan	-0.2875101	0.3620977	-0.7940125	0.4310
		**HDI.stan:Paa.stan**	0.2032549	0.3302871	0.6153885	0.5411
		GDP.stan:LE.stan	0.4558597	0.5059406	0.9010142	0.3720
		GDP.stan:AM.stan	0.0950661	0.3254236	0.2921304	0.7714
		**GDP.stan:Paa.stan**	0.1437945	0.2364866	0.6080453	0.5460
		LE.stan:AM.stan	0.3876597	0.3493764	11.095.761	0.2726
		**LE.stan:Paa.stan**	-0.1348261	0.2852400	-0.4726761	0.6385
		**AM.stan:Paa.stan**	-0.3140348	0.1975644	-15.895.311	0.1184

Model: prostate.stan ~ HDI.stan + GDP.stan + LE.stan + AM.stan + **protaa.stan** + HDI.stan:LE.stan + GDP.stan:LE.stan	R^2=0.69		Value	Std.Error	t-value	p-value
	P<0.00001	Intercept)	-0.2479539	0.14023930	-1.768.077	0.0824
		HDI.stan	-0.0978480	0.19047690	-0.513700	0.6094
		GDP.stan	-0.3052242	0.23272760	-1.311.508	0.1949
		LE.stan	0.5928169	0.18631456	3.181.807	0.0024
		AM.stan	0.5636479	0.15286343	3.687.265	0.0005
		**protaa.stan**	-0.1841924	0.08575988	-2.147.769	0.0360
		HDI.stan:LE.stan	-0.3560967	0.16987708	-2.096.202	0.0405
		GDP.stan:LE.stan	0.7597225	0.26006544	2.921.274	0.0050
Model: prostate.stan ~ GDP.stan + LE.stan + AM.stan + **kcalaa.stan** + **GDP.stan:kcalaa.stan** + **LE.stan:kcalaa.stan** + GDP.stan:LE.stan	R^2=0.70		Value	Std.Error	t-value	p-value
	P<0.00001	Intercept)	-0.2446642	0.13215833	-1.851.296	0.0693
		GDP.stan	-0.1156806	0.18446143	-0.627126	0.5331
		LE.stan	0.3428353	0.19911470	1.721.798	0.0905
		AM.stan	0.5484881	0.14500508	3.782.544	0.0004
		**kcalaa.stan**	-0.1355980	0.08394716	-1.615.278	0.1118
		**GDP.stan:kcalaa.stan**	0.4550747	0.21634470	2.103.470	0.0398
		**LE.stan:kcalaa.stan**	-0.5302630	0.23784831	-2.229.417	0.0297
		GDP.stan:LE.stan	0.3903479	0.15712121	2.484.374	0.0159
Model: prostate.stan ~ HDI.stan + GDP.stan + LE.stan + AM.stan + **Nalc.stan** + HDI.stan:LE.stan + GDP.stan:LE.stan	R^2=0.69		Value	Std.Error	t-value	p-value
	P<0.00001	Intercept)	-0.1854500	0.13511246	-1.372.560	0.1753
		HDI.stan	-0.0678939	0.18795796	-0.361218	0.7193
		GDP.stan	-0.2942754	0.22873538	-1.286.532	0.2035
		LE.stan	0.4339861	0.16942964	2.561.453	0.0131
		AM.stan	0.6980882	0.15741611	4.434.668	0.0000
		**Nalc.stan**	-0.2005077	0.08071421	-2.484.169	0.0159
		HDI.stan:LE.stan	-0.3398437	0.16664083	-2.039.379	0.0461
		GDP.stan:LE.stan	0.6539487	0.24763387	2.640.789	0.0107
Model: prostate.stan ~ HDI.stan + GDP.stan + LE.stan + AM.stan + **NPalc.stan** + HDI.stan:LE.stan + GDP.stan:LE.stan + **AM.stan:NPalc.stan**	R^2=0.72		Value	Std.Error	t-value	p-value
	P<0.00001	Intercept)	-0.2496809	0.13251403	-1.884.185	0.0647
		HDI.stan	0.0284919	0.18586150	0.153297	0.8787
		GDP.stan	-0.3397900	0.22220194	-1.529.195	0.1318
		LE.stan	0.4214767	0.16557801	2.545.487	0.0137
		AM.stan	0.4847625	0.14880195	3.257.770	0.0019
		**NPalc.stan**	-0.2445315	0.07961447	-3.071.445	0.0033
		HDI.stan:LE.stan	-0.3275774	0.16067680	-2.038.735	0.0462
		GDP.stan:LE.stan	0.6382214	0.23922077	2.667.918	0.0100
		**AM.stan:NPalc.stan**	-0.2655282	0.10520803	-2.523.840	0.0145
Model: prostate.stan ~ HDI.stan + GDP.stan + AM.stan + **ttav.stan** + **HDI.stan:ttav.stan** + **GDP.stan:ttav.stan**	R^2=0.70		Value	Std.Error	t-value	p-value
	P<0.00001	Intercept)	-0.0345784	0.1161255	-0.297768	0.7669
		HDI.stan	-0.1432484	0.1720344	-0.832673	0.4084

		GDP.stan	-0.0843775	0.1638145	-0.515080	0.6085
		AM.stan	0.6135735	0.1478172	4.150.894	0.0001
		**ttav.stan**	0.3643572	0.1306774	2.788.219	0.0072
		**HDI.stan:ttav.stan**	-0.4337338	0.1758011	-2.467.184	0.0166
		**GDP.stan:ttav.stan**	0.4866450	0.1794667	2.711.618	0.0088
Model: prostate.stan ~ GDP.stan + AM.stan + **Ntav.stan** + GDP.stan:AM.stan	R^2=0.72		Value	Std.Error	t-value	p-value
	P<0.00001	Intercept)	-0.2438755	0.1131148	-2.156.001	0.0351
		GDP.stan	-0.3134107	0.1572193	-1.993.462	0.0508
		AM.stan	0.6581662	0.1216449	5.410.553	0.0000
		**Ntav.stan**	0.5037890	0.1039250	4.847.620	0.0000
		GDP.stan:AM.stan	0.3161132	0.1175995	2.688.048	0.0093
Model: prostate.stan ~ HDI.stan + AM.stan + **Ptav.stan** + **HDI.stan:Ptav.stan** + **AM.stan:Ptav.stan**	R^2=0.75		Value	Std.Error	t-value	p-value
	P<0.00001	Intercept)	-0.0922035	0.09828973	-0.938078	0.3520
		HDI.stan	-0.1838966	0.13315418	-1.381.080	0.1725
		AM.stan	0.5857907	0.11477901	5.103.639	0.0000
		**Ptav.stan**	0.5193263	0.10918003	4.756.605	0.0000
		**HDI.stan:Ptav.stan**	-0.2924716	0.11055233	-2.645.549	0.0104
		**AM.stan:Ptav.stan**	0.4668617	0.13681892	3.412.260	0.0012
Model: prostate.stan ~ HDI.stan + LE.stan + AM.stan + **prottav.stan** + HDI.stan:LE.stan + **AM.stan:prottav.stan**	R^2=0.74		Value	Std.Error	t-value	p-value
	P<0.00001	Intercept)	-0.1354370	0.1097144	-1.234.451	0.2220
		HDI.stan	-0.1730026	0.1480284	-1.168.712	0.2473
		LE.stan	0.0987695	0.1630824	0.605641	0.5471
		AM.stan	0.6000416	0.1325240	4.527.797	0.0000
		**prottav.stan**	0.3411980	0.1166536	2.924.881	0.0049
		HDI.stan:LE.stan	-0.2024061	0.1007766	-2.008.463	0.0493
		**AM.stan:prottav.stan**	0.4549739	0.1294896	3.513.595	0.0009
Model: prostate.stan ~ HDI.stan + GDP.stan + LE.stan + AM.stan + **kcaltav.stan** + HDI.stan:GDP.stan + **HDI.stan:kcaltav.stan** + **LE.stan:kcaltav.stan** + **AM.stan:kcaltav.stan**	R^2=0.74		Value	Std.Error	t-value	p-value
	P<0.00001	Intercept)	-0.4243895	0.1973259	-2.150.703	0.0359
		HDI.stan	-0.4045208	0.2128411	-1.900.576	0.0626
		GDP.stan	-0.5581502	0.2420878	-2.305.569	0.0249
		LE.stan	0.8178101	0.1972996	4.145.017	0.0001
		AM.stan	0.5838316	0.1619020	3.606.080	0.0007
		**kcaltav.stan**	0.4369703	0.2121826	2.059.407	0.0442
		HDI.stan:GDP.stan	0.6340923	0.2054887	3.085.777	0.0032
		**HDI.stan:kcaltav.stan**	-14.103.464	0.3185589	-4.427.270	0.0000
		**LE.stan:kcaltav.stan**	0.9632001	0.2460550	3.914.573	0.0003
		**AM.stan:kcaltav.stan**	0.4218178	0.1910434	2.207.968	0.0314

Table S9. Country relationships between national leaf expectancy at birth (period 1960-2010) and various traits of annual per capita intake during the same period. The bold type indicates statistical significance (*P*<0.01).

**Per capita national food intake (mean for 1960-2009)**	**National leaf expectance at birth (mean for 1960-2009)**
Total vegetable intake (Tv)	R=0.10<br>*P*=0.30
Total N intake from vegetables (Nv)	R=-0.045<br>*P*=0.65
Total P intake from vegetables (Pv)	R=-0.20<br>*P*=0.043
**Total protein intake from vegetables (Protv)**	**R=-0.34**<br>***P*<0.00001**
**Total kilocalories intake from vegetables (Kcalv)**	R=0.094<br>*P*=0.30
**Total terrestrial animals intake (Tta)**	**R=0.77**<br>***P*<0.00001**
**Total N intake from terrestrial animals (Nta)**	**R=0.77**<br>***P*<0.00001**
**Total P intake from terrestrial animals (Pta)**	**R=0.72**<br>***P*<0.00001**
**Total protein intake from terrestrial animals (Protta)**	**R=0.81**<br>***P*<0.00001**
**Total kilocalories intake from terrestrial animals (Kcalta)**	**R=0.79**<br>***P*<0.00001**
**Total aquatic animals intake (Taa)**	**R=0.36**<br>***P*<0.00001**
**Total N intake from aquatic animals (Naa)**	**R=0.34**<br>***P*<0.00001**
**Total P intake from aquatic animals (Paa)**	**R=0.34**<br>***P*<0.00001**
**Total protein intake from aquatic animals (Protaa)**	**R=0.35**<br>***P*<0.00001**
**Total kilocalories intake from aquatic animals (Kcalaa)**	**R=0.32**<br>***P*<0.00001**
**N:P ratio from terrestrial animals (NPta)**	**R=-0.20**<br>***P*=0.036**
**N:P ratio from aquatic animals** (NPaa)	**R=-0.37**<br>***P*<0.00001**
**N:P ratio from vegetables (NPv)**	**R=0.29**<br>***P*=0.002**
**Ratio of intake of terrestrial animal/vegetable foods (Ttav)**	**R=0.66**<br>***P*<0.00001**
**Ratio of N intake from terrestrial animal/vegetable foods (Ntav)**	**R=0.77**<br>***P*<0.00001**
**Ratio of P intake from terrestrial animal/vegetable foods (Ptav)**	**R=0.76**<br>***P*<0.00001**
**Ratio of protein intake from terrestrial animal/vegetable foods (Prottav)**	**R=0.79**<br>***P*<0.00001**
**Ratio of kilocalories intake from terrestrial animal/vegetable foods (Kcaltav)**	**R=0.75**<br>***P*<0.00001**
**Total intake of alcoholic beverages (Talc)**	***R*=0.39**<br>***P*<0.0001**
**Total N intake from alcoholic beverages (Nalc)**	***R*=-0.30**<br>***P*=0.002**
Total P intake from alcoholic beverages (Palc)	*R*=0.20<br>*P*=0.033
**Total kilocalories from alcoholic beverages (Kcalalc)**	***R*=0.50**<br>***P*<0.0001**
Total protein intake from alcoholic beverages (Protalc)	*R*=0.21<br>*P*=0.033

Table S10. Best linear models accounting for life expectancy at birth (LE) as functions of national per capita wealth (using GDP), the human development index (HDI), mean age of the population (MA) and mean per capita intake of food from different sources during the period 1960-2010. The value provided is the standardized value.

**Leaf expectance at birth (LE) (period 1960-2009)**						
**Model**	**Statistical results of the model**	**Independent factor statistics**				
Model:;LE.stan;~;HDI.stan;+;GDP.stan;+;**tta.stan**;+;HDI.stan:GDP.stan;	R^2=0.79		Value	Std.Error	t-value	p-value
	P<0.0001	Intercept)	0.2256963	0.10216836	2.209.063	0.0294
		HDI.stan	0.5552385	0.11657244	4.763.034	0.0000
		GDP.stan	0.3349724	0.18764531	1.785.136	0.0772
		**tta.stan**	0.2115017	0.07908712	2.674.287	0.0087
		HDI.stan:GDP.stan	-0.2992112	0.12157857	-2.461.052	0.0155
Model:;LE.stan;~;HDI.stan;+;GDP.stan;+;**Nta.stan**;+;HDI.stan:GDP.stan;	R^2=0.80		Value	Std.Error	t-value	p-value
	P<0.0001	Intercept)	0.2202169	0.10055392	2.190.038	0.0308
		HDI.stan	0.5368863	0.11506988	4.665.741	0.0000
		GDP.stan	0.3167931	0.18413414	1.720.448	0.0884
		**Nta.stan**	0.2498355	0.07619773	3.278.779	0.0014
		HDI.stan:GDP.stan	-0.2919470	0.11966162	-2.439.771	0.0164
Model:;LE.stan;~;HDI.stan;+;**protta.stan**;+;**HDI.stan:protta.stan**	R^2=0.78		Value	Std.Error	t-value	p-value
	P<0.0001	Intercept)	0.1097952	0.06693861	1.640.237	0.1040
		HDI.stan	0.6638177	0.08267633	8.029.114	0.0000
		**protta.stan**	0.2935317	0.09421728	3.115.477	0.0024
		**HDI.stan:protta.stan**	-0.1394874	0.06229983	-2.238.969	0.0273
Model:;LE.stan;~;HDI.stan;+;**kcalta.stan**;+;**HDI.stan:kcalta.stan**;	R^2=0.78		Value	Std.Error	t-value	p-value
	P<0.0001	Intercept)	0.1326295	0.06841893	1.938.492	0.0553
		HDI.stan	0.6511326	0.08234596	7.907.282	0.0000
		**kcalta.stan**	0.3277164	0.09885435	3.315.144	0.0013
		HDI.stan:kcalta.stan	-0.1726577	0.06673396	-2.587.255	0.0111
Model:;LE.stan;~;HDI.stan;+;GDP.stan;+;AM.stan;+;**NPta.stan**;+;HDI.stan:GDP.stan;+;**GDP.stan:NPta.stan**;+;**AM.stan:NPta.stan**;	R^2=0.85		Value	Std.Error	t-value	p-value
	P<0.0001	Intercept)	0.2981490	0.08760640	3.403.279	0.0010
		HDI.stan	0.5471325	0.10074876	5.430.663	0.0000
		GDP.stan	0.3958371	0.16120443	2.455.498	0.0158
		AM.stan	0.1788642	0.08108308	2.205.937	0.0297
		**NPta.stan**	-0.0252707	0.05227262	-0.483440	0.6298
		HDI.stan:GDP.stan	-0.3798696	0.10644868	-3.568.570	0.0006

			Value	Std.Error	t-value	p-value
		**GDP.stan:NPta.stan**	-0.6255470	0.10593677	-5.904.909	0.0000
		**AM.stan:NPta.stan**	0.6536716	0.09775100	6.687.109	0.0000
Model:;LE.stan;~;HDI.stan;+;GDP.stan;+;**ttav.stan**;+;HDI.stan:GDP.stan;	R^2=0.79 P<0.0001		Value	Std.Error	t-value	p-value
		Intercept)	0.2329547	0.10257170	2.271.140	0.0252
		HDI.stan	0.5753733	0.11586353	4.965.957	0.0000
		GDP.stan	0.3890065	0.18461925	2.107.075	0.0375
		**ttav.stan**	0.1585965	0.06283982	2.523.822	0.0131
		HDI.stan:GDP.stan	-0.3088338	0.12206963	-2.529.981	0.0129
Model:;LE.stan;~;HDI.stan;+;GDP.stan;+;**Ntav.stan**;+;HDI.stan:GDP.stan;	R^2=0.80 P<0.0001		Value	Std.Error	t-value	p-value
		Intercept)	0.2295161	0.10075304	2.278.006	0.0248
		HDI.stan	0.5248922	0.11627181	4.514.355	0.0000
		GDP.stan	0.3552078	0.18224534	1.949.064	0.0540
		**Ntav.stan**	0.2337514	0.07289504	3.206.685	0.0018
		HDI.stan:GDP.stan	-0.3042752	0.11989623	-2.537.821	0.0127
Model:;LE.stan;~;HDI.stan;+;GDP.stan;+;**Ptav.stan**;+;HDI.stan:GDP.stan;	R^2=0.79 P<0.0001		Value	Std.Error	t-value	p-value
		Intercept)	0.2399330	0.10282219	2.333.475	0.0216
		HDI.stan	0.5400405	0.11895053	4.540.043	0.0000
		GDP.stan	0.3936709	0.18466612	2.131.798	0.0354
		**Ptav.stan**	0.1818734	0.07368959	2.468.102	0.0152
		HDI.stan:GDP.stan	-0.3180851	0.12240207	-2.598.691	0.0107
Model:;LE.stan;~;HDI.stan;+;**prottav.stan**;+;**HDI.stan:prottav.stan**;	R^2=0.79 P<0.0001		Value	Std.Error	t-value	p-value
		Intercept)	0.1327076	0.06639104	1.998.877	0.0482
		HDI.stan	0.6464665	0.08156190	7.926.085	0.0000
		**prottav.stan**	0.3267014	0.09495356	3.440.645	0.0008
		**HDI.stan:prottav.stan**	-0.1712420	0.06287104	-2.723.702	0.0076
Model:;LE.stan;~;HDI.stan;+;**kcaltav.stan**;+;**HDI.stan:kcaltav.stan**;	R^2=0.48 P<0.0001		Value	Std.Error	t-value	p-value
		Intercept)	0.1034175	0.06519802	1.586.207	0.1157
		HDI.stan	0.7175827	0.07438865	9.646.401	0.0000
		**kcaltav.stan**	0.2461162	0.08801098	2.796.426	0.0062
		**HDI.stan:kcaltav.stan**	-0.1407535	0.06300709	-2.233.931	0.0276

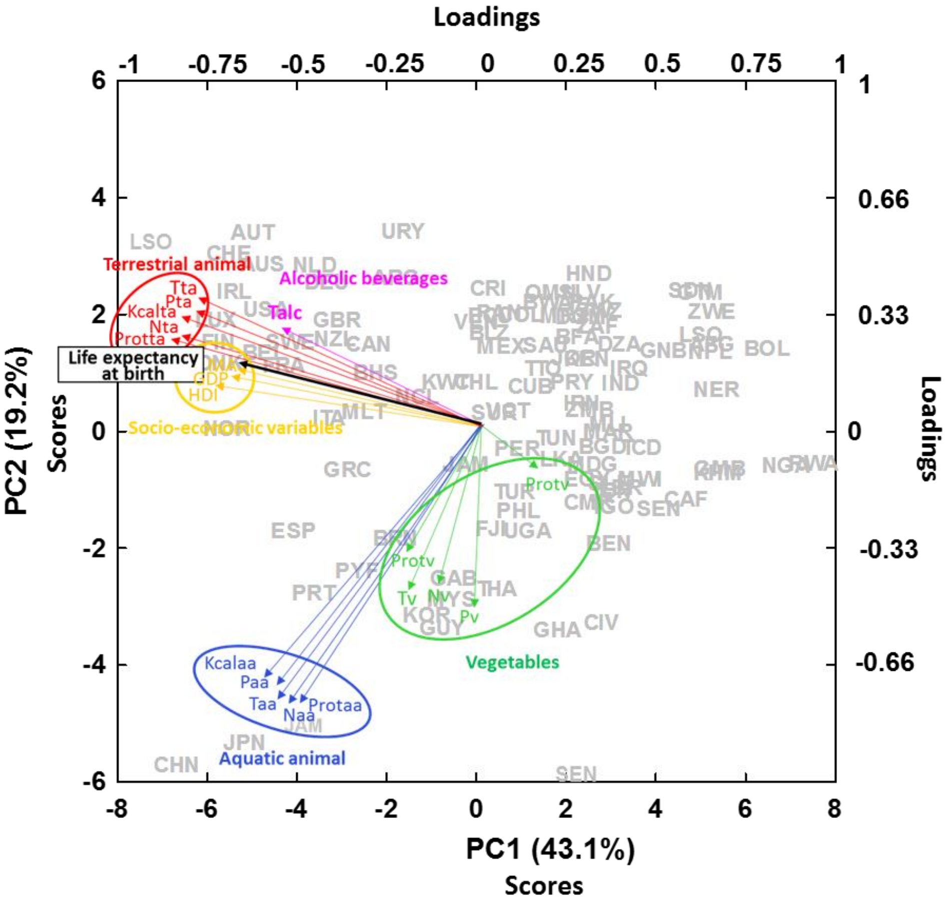

Figure S1. PCA with mean averages of country expectancy at birth (LE) (red), different socioeconomic variables (MA=mean age, HDI=human development index, GDP gross domestic product per capita) (blue) and food sources (t=total, N=nitrogen intake, P=phosphorus intake, prot=protein and kcal=Kilocalories per capita intake) from terrestrial animal sources (ta) (velvet), vegetables (v) (green) and aquatic animal (aa) (black) in the period 1960-2010. AFG=Afghanistan, DZA=Algeria, ARG=Argentina, AUS=Australia, AUT=Austria, BHS=Bahamas, BGD=Bangladesh, BEL=Belgium, BLZ=Belize, BEN=Benin, BOL=Bolivia , BWA=Botswana, BRN=Brunei Darussalam, BFA=Burkina Faso, KHM=Cambodia, CMR=Cameroon, CAN=Canada, CAF=Central African Republic, TCD=Chad, CHL=Chile, CHN=China, COL=Colombia, CRI=Costa Rica, CIV=Cote Ivoire, CUB=Cuba, DNK=Denmark, DOM=Dominican Republic, ECU=Ecuador, EGY=Egypt, SLV=El Salvador, FJI=Fiji, FIN=Finland, FRA=France, PYF=French Polynesia, GAB=Gabon, GMB=Gambia, DEU=Germany, GHA=Ghana, GRC=Greece, GTM=Guatemala, GNB=Guinea-Bissau, GUY=Guyana, HND=Honduras, ISL=Iceland, IND=India, IDN=Indonesia, IRN=Iran , IRQ=Iraq, IRL=Ireland, ITA=Italy, JAM=Jamaica, JPN=Japan, JOR=Jordan, KEN=Kenya, KIR=Kiribati, KOR=Korea, KWT=Kuwait, LSO=Lesotho, LBR=Liberia, LUX=Luxembourg, MDG=Madagascar, MWI=Malawi, MYS=Malaysia, MLI=Mali, MLT=Malta, MRT=Mauritania, MEX=Mexico, MAR=Morocco, NPL=Nepal, NLD=Netherlands, NCL=New Caledonia, NZL=New Zealand, NER=Niger, NGA=Nigeria, NOR=Norway, OMN=Oman, PAK=Pakistan, PAN=Panama, PRY=Paraguay, PER=Peru, PHL=Philippines, PRT=Portugal, RWA=Rwanda, VCT=Saint Vincent and the Grenadines, SAU=Saudi Arabia, SEN=Senegal, SLE=Sierra Leone, SLB=Solomon Islands, ZAF=South Africa, ESP=Spain, LKA=Sri Lanka, SDN=Sudan, SUR=Suriname, SWZ=Swaziland, SWE=Sweden, CHE=Switzerland, THA=Thailand, TGO=Togo, TTO=Trinidad and Tobago, TUN=Tunisia, TUR=Turkey, UGA=Uganda, GBR=United Kingdom, USA=United States of America, URY=Uruguay, VEN=Venezuela , ZMB=Zambia, ZWE=Zimbabwe.
